# Photocatalytic Dehalogenation of Aryl Halides Mediated by the Flexible Metal–Organic Framework MIL‐53(Cr)

**DOI:** 10.1002/anie.202422776

**Published:** 2025-01-13

**Authors:** Tian Luo, Henrik S. Jeppesen, Alexander Schoekel, Nadine Bönisch, Fei Xu, Rong Zhuang, Qiang Huang, Irena Senkovska, Volodymyr Bon, Thomas Heine, Agnieszka Kuc, Stefan Kaskel

**Affiliations:** ^1^ Chair of Inorganic Chemistry I Technische Universität Dresden Bergstr. 66 01069 Dresden Germany; ^2^ Deutsches Elektronensynchrotron DESY Notkestr. 85 22607 Hamburg Germany; ^3^ School of Materials Science and Engineering Northwestern Polytechnical University 710072 Xi'An P. R. China; ^4^ Helmholtz-Zentrum Dresden-Rossendorf, HZDR Bautzner Landstr. 400 01328 Dresden Germany; ^5^ Center for Advanced Systems Understanding, CASUS Conrad-Schiedt-Straße 20 02826 Görlitz Germany; ^6^ Yonsei University ibs-cnm Seodaemun-gu 120-749 Seoul Republic of Korea; ^7^ Chair of Theoretical Chemistry Technische Universität Dresden Bergstr. 66c 01069 Dresden Germany

**Keywords:** Photocatalysis, MIL-53(Cr), flexibility, crystal engineering, DFT calculation

## Abstract

The catalytic potential of flexible metal–organic frameworks (MOFs) remains underexplored, particularly in liquid‐phase reactions. This study employs MIL‐53(Cr), a prototypical “breathing” MOF capable of structural adaptation via pore size modulation, as a photocatalyst for the dehalogenation of aryl halides. Powder X‐ray diffraction and Pair Distribution Function analyses reveal that organic solvents influence pore opening, while substrates and products dynamically adjust the framework configuration during catalysis. This structural flexibility enables precise tuning of photocatalytic efficiency via solvent‐mediated control of the pore aperture. The results demonstrate that the dynamic behavior of MIL‐53(Cr) facilitates enhanced catalytic activity and selectivity, advancing the application of flexible MOFs as tunable, enzyme‐mimicking catalysts. These findings pave the way for the rational design of next‐generation flexible photocatalysts.

## Graphical abstract

MIL‐53(Cr), a flexible “breathing” metal–organic framework (MOF), exhibits exceptional photocatalytic performance in the dehalogenation of aryl halides. Structural flexibility, driven by the interactions of the MOF with the solvent and substrate, tunes the pore apertures and enhances catalytic efficiency. This study unveils MIL‐53(Cr)′s dynamic framework reconfiguration, highlighting its potential as a tunable, enzyme‐mimicking photocatalyst for sustainable chemical processes.

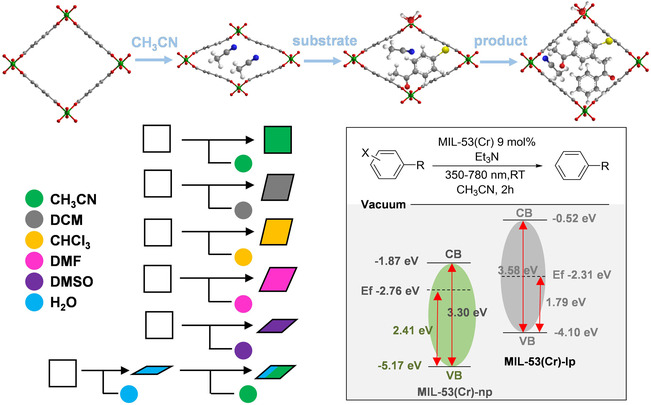

## Introduction

Featuring a new kind of highly porous material with a designable structure attributing to the inorganic‐organic hybrid nature, and consequently, combining the properties of semiconductors and molecular catalysts with tailorable energy level structure, metal–organic frameworks (MOFs) have been widely exploited as photocatalysts for CO_2_ reduction,^[1]^ water splitting,[^2^, ^3^] degradation,^[4]^ and organic redox reactions.[^5^, ^6^, ^7^] Among the huge number of reported MOFs,^[8]^ selected frameworks show the stimuli‐induced dynamics, often induced by external pressure, temperature, and adsorbed guest species.[^9^, ^10^] The implications of MOFs flexibility have been explored in gas storage,^[11]^ and the adaptive pore size change has been recently identified to promote high selectivity for gas separation, sensing, and actuator design.^[12]^ The idea to utilize the MOF switchability to achieve a biomimicking catalyst with superior catalytic performance has been intensively discussed,[^13^, ^14^] but the flexibility has often been neglected, and the dynamic effects have, to the best of our knowledge, not been explored in details experimentally, although some computational studies have been published.[^15^, ^16^] Obviously, the deliberate control over multiple decisive factors in catalysis is hard to realize.[^17^, ^18^] The most recent progress has been made by using flexible frameworks PCN‐700^[19]^ or NU‐1400,^[20]^ either as the fully open phase or the dense closed phase, which demonstrates a switch “on” and “off” effect on catalytic activity. An alternative approach of confinement control involves creating a dynamic microenvironment through the metal clusters^[21]^ or ligand modification.^[22]^

However, the phase transition and structure configuration upon the adsorption of different solvents, substrates, and corresponding product molecules have not even been taken into account so far.^[24]^ The subtle differences in adsorption enthalpy, polarity, shape, and kinetic diameter of different guest molecules may influence the guest‐response properties and stabilize phases with well‐defined pore apertures, resembling the lock‐and‐key principle of enzyme action.[^25^, ^26^] The adaptive response of the framework structure may cause not only the changes in the redox capability of MOF, but also the pore size and volume changes, which also, in turn, affects the catalyst geometry and reactivity. Moreover, when exposed to a reactant, the catalytically active site may change the local structure, leading to a framework deformation and changes in the pore structure.

**Figure 1:**
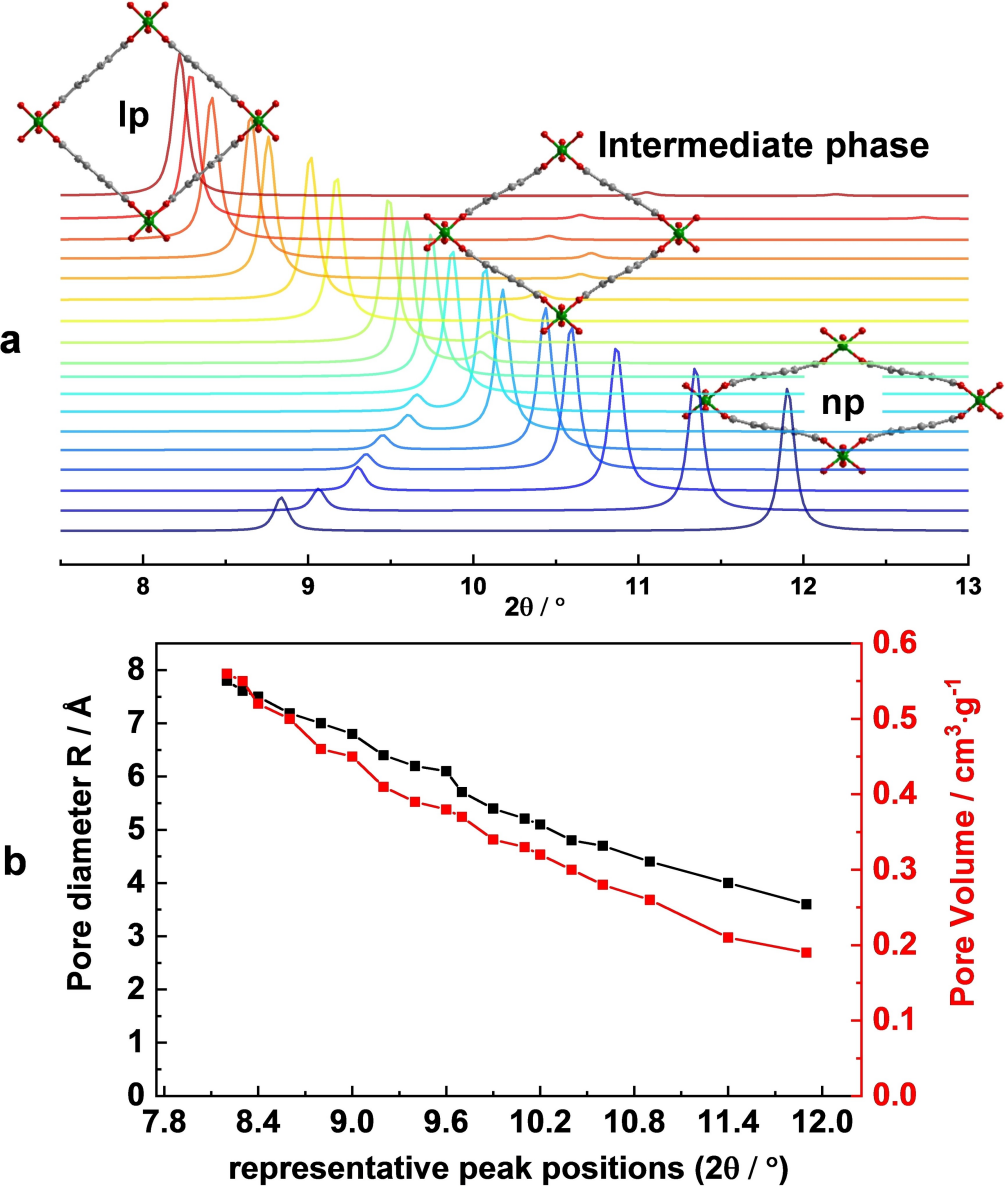
Structure information of simulated MIL‐53(Cr) with varying pore size and pore volume: (**a**) Simulated PXRD patterns for pore aperture diameter R ranging from 3.6 Å to 7.8 Å; (**b**) The plot of 2*θ* values of characteristic peaks from PXRD patterns and corresponding pore diameters/pore volume.

As a prominent and representative example of flexible MOF, [Cr(OH)(bdc)]_n_ (MIL‐53(Cr), bdc‐1,4‐benzenedicarboxylate) has gained attention due to the very large breathing effect coupled to adsorption/desorption of guest molecules.[^27^, ^28^] MIL‐53(Cr) structure consists of Cr−OH−Cr chain‐like secondary building units (SBUs), resulting from Cr^III^ octahedra linked by *μ_2_*‐OH groups, which are further bridged by terephthalate linkers into a 3D framework to form one‐dimensional channels.^[29]^ In terms of crystal structure, the large pore (lp) phase structure of MIL‐53(Cr) crystallizes in the orthorhombic space group *Imma*. Assuming that the cell parameter associated with the [−Cr−OH−Cr−]_n_ chains remains constant and the space group symmetry does not change, a variety of potential intermediate MIL‐53(Cr) structures with pore aperture diameter (*R*) ranging between 7.80 Å to 3.60 Å were simulated (VdW considered, Figure 1 and Table S1). The analysis of pore size distributions of the hypothetical MIL‐53(Cr) structures using Zeo++ software^[30]^ (Figure S1) features pore accessibility with a free aperture (*R*) of 7.80 Å for MIL‐53‐lp (large pore) and *R*=3.60 Å for MIL‐53(Cr)‐np (narrow pore) phase. The pores should be accessible only for small molecules, such as H_2_O or CO_2_.^[31]^ Moreover, MIL‐53 series have also been reported as chemically robust MOFs in various organic solvents, broad pH ranges, and temperatures.[^32^, ^33^] Furthermore, Cr(III)‐based MOFs have a wide optical absorption window encompassing the visible light region, which is beneficial for light utilization, and the energy required for electronic transition is lower than those materials that only absorb in the ultraviolet region.[^34^, ^35^, ^36^, ^37^] These features rank MIL‐53(Cr) as a promising photocatalyst candidate.

**Scheme 1:**
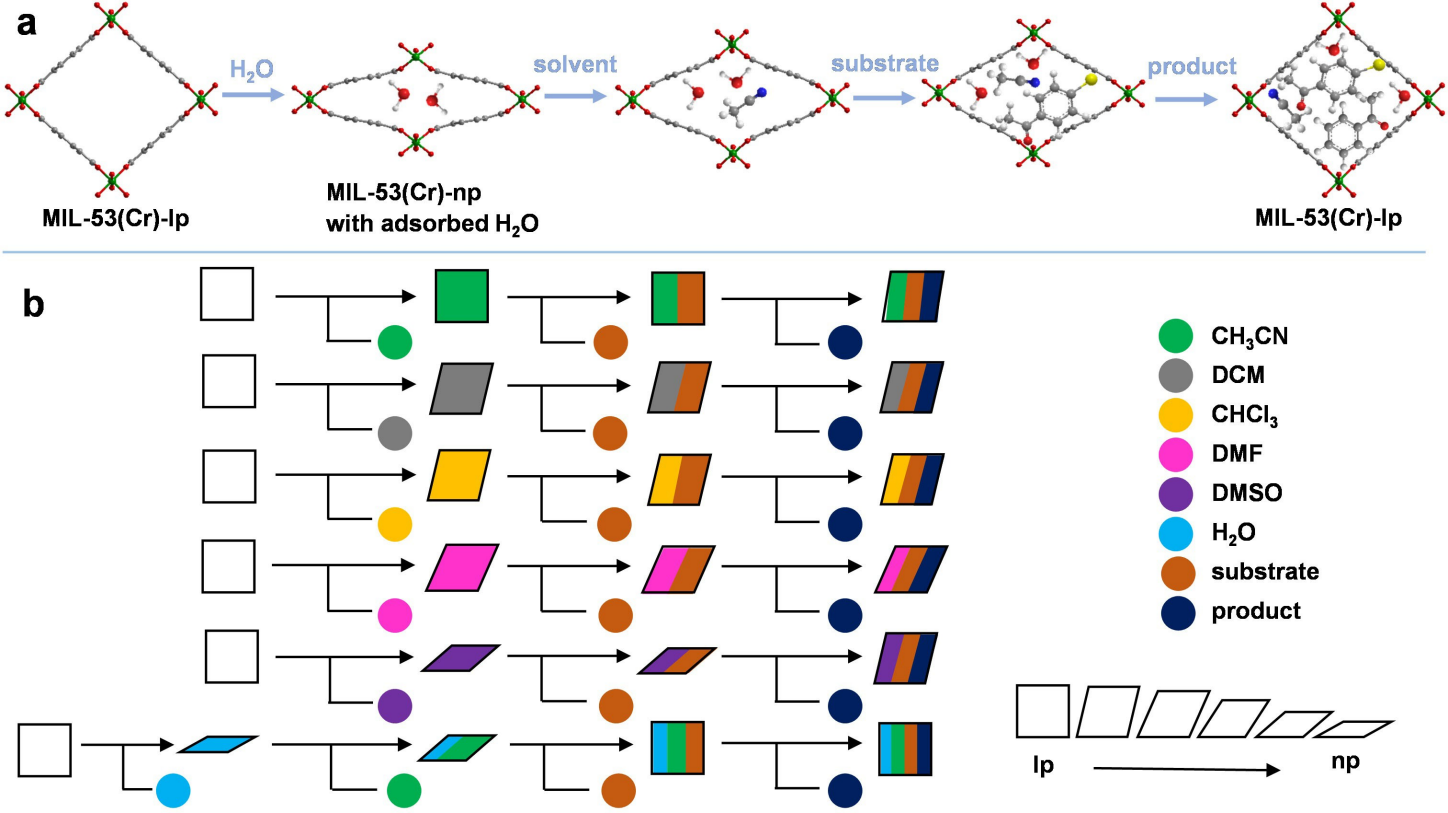
Elementary symbol for illustration of the structure deformations in MIL‐53(Cr) depending on guest molecules adsorbed. (**a**) The view on the crystal structures with different guest molecules adsorbed; (**b**) Elementary step representation for breathing dynamics upon solvent molecules adsorption, the introduction of substrate molecules, and the generated product molecules. The different colors of circles represent different molecular stimuli.^[23]^

Here, we present the photocatalytic performance of flexible MIL‐53(Cr) in a dehalogenation reaction, a model reaction in photocatalysis with promising industrial applications for treating halogen‐containing wastewater. MIL‐53(Cr) exhibits outstanding optical properties and strong visible light absorption, with a band gap of 3.30 eV, making it an excellent photocatalyst. The phase transitions and structural configurations upon the adsorption of selected organic solvents, their mixtures, the substrate and product molecules generated during the reaction process were characterized by powder X‐ray diffraction (PXRD) and pair distribution function (PDF) analysis, revealing a pivotal influence of molecules nature on the pore configuration of MIL‐53(Cr) (Scheme 1). The introduction of the substrate and the formation of the corresponding product in the pores also induce structure reconfiguration, which is one of the main influencing factors on catalytic activity, apart from the solvent's inherent properties and solvent effects. Chemical structures of guest molecules, e.g. solvent, substrate, or product molecules strongly influence the host–guest and guest‐guest interactions in the system, which also affect the catalytic efficiency.

The combined effects described above may result in dynamic adsorption and adaptive aperture sizes, guiding the catalyst toward specific outcomes. Here, we demonstrate for the first time that the photocatalytic activity of MIL‐53(Cr) can be precisely tuned through solvent regulation and structural reconfiguration of the flexible MOF during the photocatalytic reaction, paving the way for the development of flexible catalysts.

## Results and Discussion

MIL‐53(Cr) was characterized by powder X‐ray diffraction and confirmed to be MIL‐53(Cr)‐lp phase (Figure S2a). The porosity of MIL‐53(Cr)‐lp was confirmed by N_2_ physisorption at 77 K. It exhibits a type Ia isotherm (Figure S2b), and the Brunauer–Emmett–Teller (BET) area is calculated as 1050 m^2^ ⋅ g^−1^ (see calculation method in Supporting Information). When exposed to air, the MOF adsorbs water from the atmosphere and transforms to a narrow pore phase (MIL‐53(Cr)‐np), which shows low uptake in nitrogen physisorption experiment at 77 K and the BET area is as low as 20 m^2^ ⋅ g^−1^. Visually, the colour changes from grey to dark green (Figure S3). After desolvation at temperatures above 100 °C under vacuum, the pores open again to achieve MIL‐53(Cr)‐lp (Figure S2a). The thermogravimetric analysis of MIL‐53(Cr)‐np indicates that MIL‐53(Cr) is thermally stable up to 350 °C (Figure S4a). The morphology of synthesized MIL‐53(Cr) was characterized by scanning electron microscopy (Figure S3), MIL‐53(Cr) appears as long rectangular‐shaped crystals in the average length of 3 μm.

**Figure 2:**
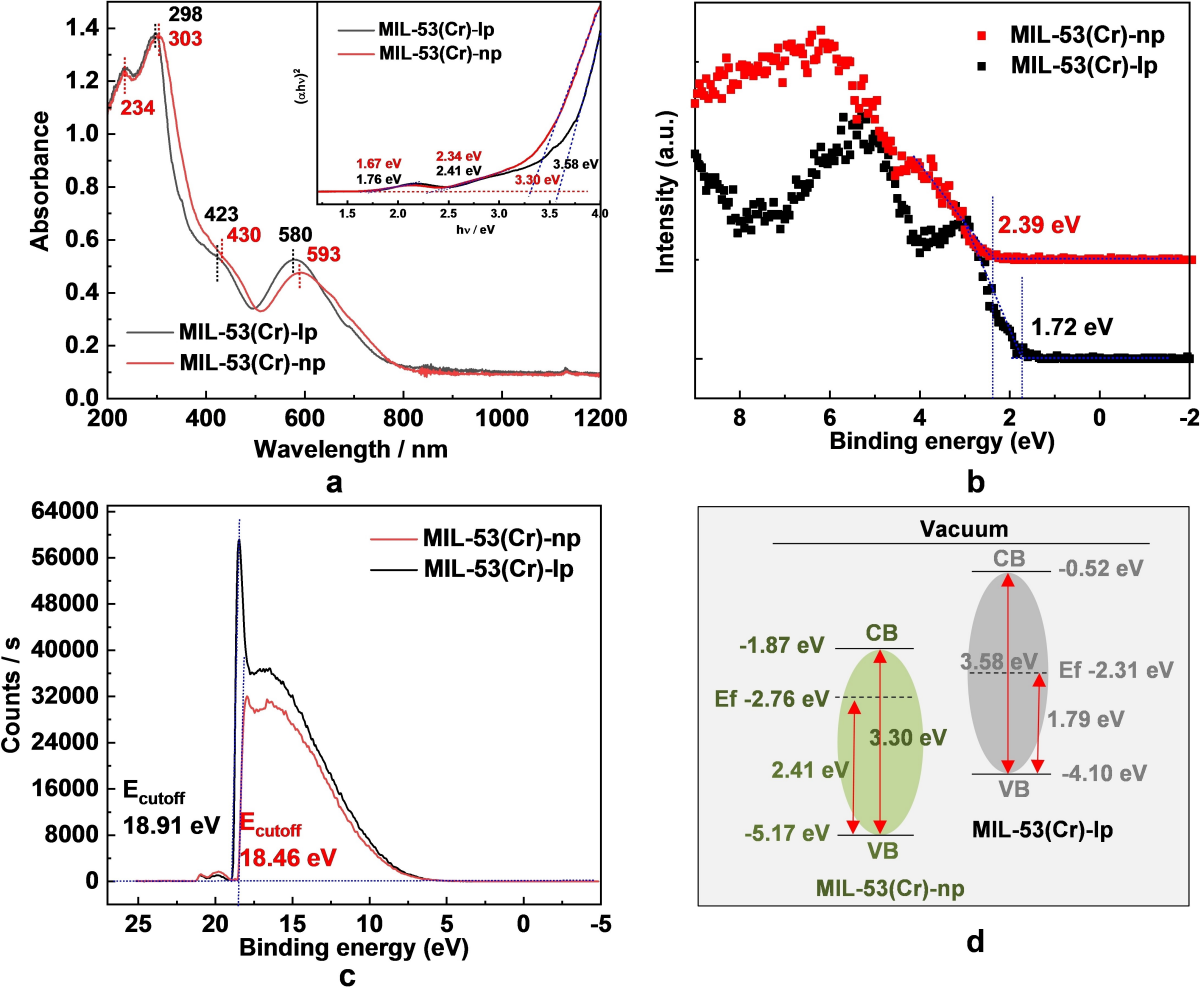
Optical characterization and band gap energetic structure determination of MIL‐53(Cr). (**a**) The UV/Vis diffuse reflectance spectra of MIL‐53(Cr)‐lp and MIL‐53(Cr)‐np, inset figure shows Tauc plots for band gap determination; (**b**) Valence band‐XPS spectra of MIL‐53(Cr)‐lp and MIL‐53(Cr)‐np; (**c**) UPS spectra of MIL‐53(Cr)‐lp and MIL‐53(Cr)‐np measured by He I (*h*ν=21.22 eV) at −5 V; (**d**) the energetic structure of MIL‐53(Cr)‐lp and MIL‐53(Cr)‐np.

The optical properties of MIL‐53(Cr) were characterized using solid‐state UV/Vis diffuse reflectance spectroscopy (UV‐DRS). MIL‐53(Cr) has a wide light absorption window up to 800 nm (Figure 2a), and the two absorption bands centered at around 430 and 590 nm are assigned to two *d‐d* transitions of Cr^3+^. The intensive absorption in the UV region is attributed to the combination of π→π* transition of the phenyl rings of the ligand, the third *d*‐*d* transition of Cr^3+^ and ligand‐to‐metal charge‐transfer (LMCT).[^34^, ^38^] The band gap values of MIL‐53(Cr)‐np and MIL‐53(Cr)‐lp were obtained from Tauc plots as 3.30 and 3.58 eV, respectively,^[39]^ which are compared with a series of known MOF photocatalysts, as listed in Table S2. To better understand the photo‐redox ability of MIL‐53(Cr), valence‐band X‐ray photoelectron spectroscopy (VB‐XPS)^[34]^ was conducted to determine the E_VB_. The VB edges of MIL‐53(Cr)‐np and MIL‐53(Cr)‐lp were observed at 2.39 eV and 1.72 eV under the Fermi level, respectively (Figure 2b and S5). It is interesting to find that different structure configurations of the same material result in different VB positions. To confirm this point, ultraviolet photoelectron spectroscopy (UPS) spectra were conducted to determine the work functions (WF). WF values of MIL‐53(Cr)‐np and MIL‐53(Cr)‐lp determining the Fermi level were calculated to be −2.76 and −2.31 eV from the measured secondary electron cutoff energy (E_cutoff_) (Figure 2c) by subtracting the excitation energy of He I (21.22 eV). The energy differences between the Fermi level and the valence band of the samples (Figure S6) were determined to be 2.41 and 1.79 eV, respectively, which are in consistence with the VB‐XPS analysis. Combined with the band‐gap values, the valence band (VB) and conduction band (CB) values of MIL‐53(Cr)‐np and MIL‐53(Cr)‐lp were calculated and noted in Figure 2d. Upon visible light irradiation at 350 ‐ 780 nm, the photo‐induced electrons from MIL‐53(Cr) in both lp and np phases have high reductive potentials, which can reduce aryl halides to get dehalogenation products accordingly (Scheme in Figure 2d).

**Figure 3:**
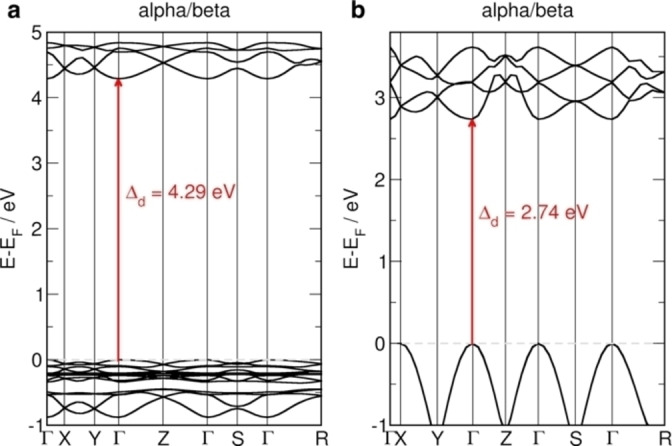
Electronic structure simulations. (**a**) MIL‐53(Cr)‐lp and (**b**) MIL‐53(Cr)‐np band structures obtained at the DFT/HSE06 level of theory. Antiferromagnetic ordering is the ground state for both systems and alpha and beta states are overlaid. Fundamental band gaps (direct, Δ_d_) are marked with red arrows. Dashed grey lines indicate the Fermi level, which was shifted to the valence band maximum and set to zero.

To confirm the experimentally derived values of band gaps for MIL‐53(Cr)‐lp and MIL‐53(Cr)‐np, we conducted DFT calculations to obtain the band structure for MIL‐53(Cr) and highlight the influence of the pore size on the band gap. The band structures for both systems, calculated using the optimized lattice parameters (see ESI) are shown in Figure 3. Both systems exhibit antiferromagnetic ordering of spins on Cr atoms within each chain and between the chains as the ground state. They are both direct band gap materials. Our simulated band gap values are different from the experimental findings, 4.29 eV (exp. 3.58 eV) and 2.74 eV (exp. 3.30 eV) for MIL‐53(Cr)‐lp and MIL‐53(Cr)‐np, respectively. This difference comes from the fact that for insulators, HSE06 functional tends to overestimate the experimental values, while for semiconductors it underestimates them. Our results agree well with other theoretical works^[39]^ and reproduce the observed trend, in which smaller pore sizes result in smaller band gap values. To confirm that, we also calculated MIL‐53(Cr)‐np with varying lattice parameters (Figure S7). The stronger interaction between linkers in the MIL‐53(Cr)‐np results also in stronger dispersion of the band edges, indicating an increase in the mobility of charge carriers. LP and NP phases of MIL‐53(Cr) differentiate in the band gap energy levels and, correspondingly, in the redox capabilities. Thus, the deformation of the MIL‐53(Cr) structure may lead to varying catalytic performance.

### Studies of Guest‐Induced Dynamics of MIL‐53(Cr)

**Figure 4:**
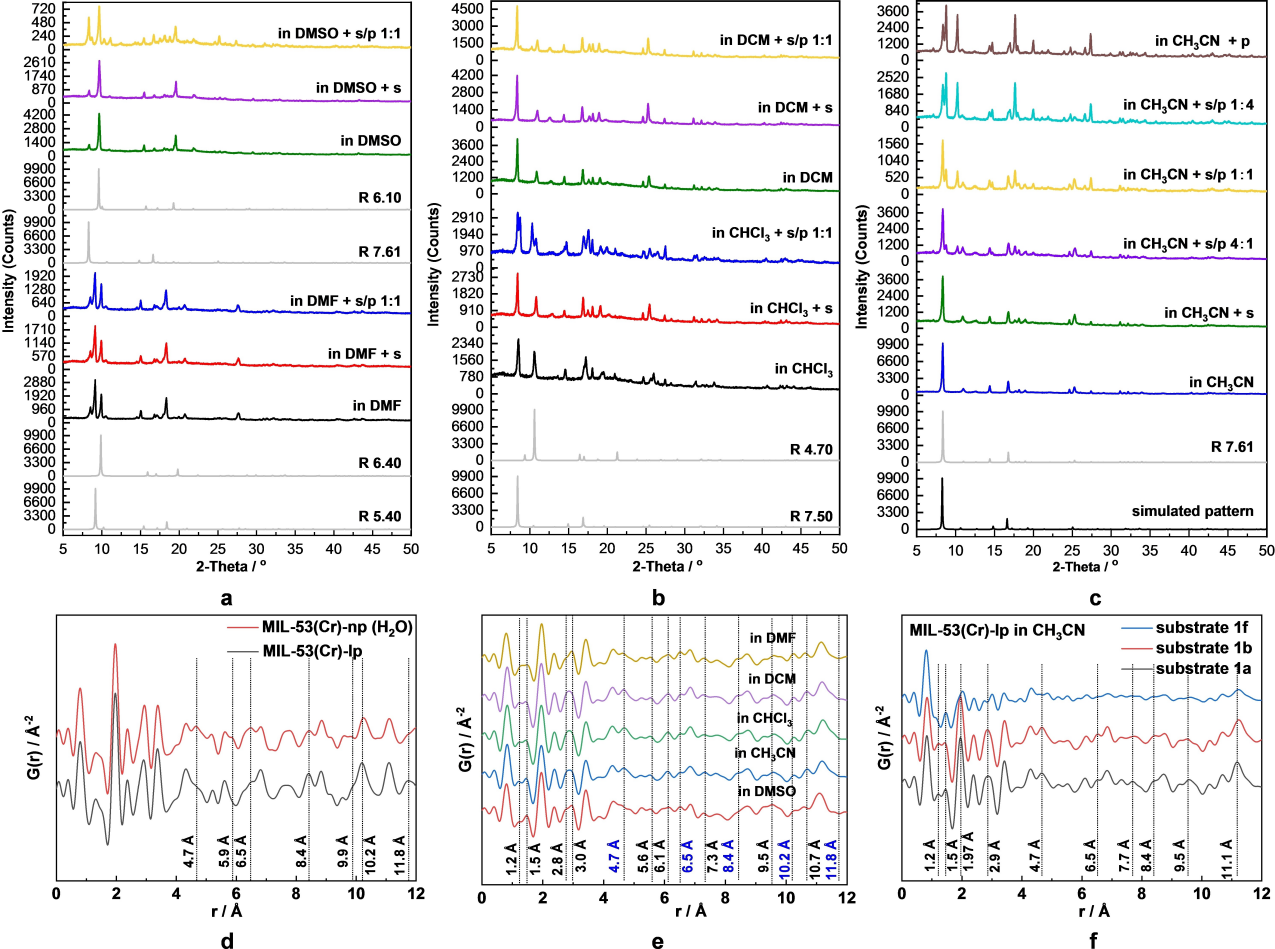
Structure reconfiguration studies of MIL‐53(Cr). (a–c) PXRD analysis: the PXRD patterns of MIL‐53(Cr)‐lp in: (**a**) DMF and DMSO, (**b**) DCM and CHCl_3_ with sole solvent molecules adsorbed, substrate (s) added, and both substrate/product (s/p 1 : 1) added; (**c**) the PXRD patterns of MIL‐53(Cr)‐lp in CH_3_CN with sole solvent molecules adsorbed, and different ratios of substrate/product added (5:0, 4 : 1, 1 : 1, 1 : 4, 0 : 5). (**d**–**f**) PDF analysis: the G(r) curves in the range of 0–12 Å^−1^ (**d**) MIL‐53(Cr) in the lp and np phases, the MIL‐53(Cr)‐np (H_2_O) is obtained by leaving the MIL‐53(Cr)‐lp in air for 12 h; (**e**) MIL‐53(Cr)‐lp loaded with substrate **1 a** in DMSO, CH_3_CN, CHCl_3_, DCM and DMF, respectively; (**f**) MIL‐53(Cr)‐lp in CH_3_CN loaded with substrate **1 a**, **1 b** and **1 f**, respectively (R – pore size).

To study the solvent‐directing role on the pore size of the MOF, the PXRD patterns of MIL‐53(Cr) loaded with five organic solvents, including CH_3_CN, *N,N*‐dimethylformamide (DMF), dimethyl sulfoxide (DMSO), dichloromethane (DCM), and CHCl_3_ (Figure 4) were collected. Since PXRD patterns revealed a mixture of phases, among other until now unknown intermediate phases, the *ab initio* indexing of the PXRD patterns was challenging. To assign the diffraction peaks observed in experimental PXRD patterns to the pore size, a series of hypothetical structural models, possessing different pore opening *R*, were constructed and optimized using Universal Force Field, implemented in Materials Studio 5.0 software. Variation of *b* and *c* unit cell parameters (16<*b*<20 Å, 8<*c*<14.5 Å) influences the pore opening of the 1D channels in MIL‐53(Cr). The *a* direction is defined by Cr−OH−Cr chains and remains constant upon breathing of the structure.

The simulated patterns generated from the structural models of MIL‐53(Cr) are shown in Figure 1 and Figure S1, the representative and most intense (011) peak is gradually shifting from 2*θ*=8.2° (lp phase) to 2*θ*=11.9° (np phase) along the trajectory of structural transition. The (020) peak shifts from 2*θ*=11.1° to 2*θ*=8.8°. Comparison of PXRD patterns calculated from model structures with experimentally measured ones allows to determine the phase composition in the mixture.

For example, MIL‐53(Cr) in CH_3_CN (Figure 4c) represents a pure phase, with *a*=6.83, *b*=16.05, *c*=13.99 Å and pore size of 7.60 Å. When applying the strategy mentioned above and comparing it with simulated patterns, the representative peak at 8.3° indicates that it corresponds to the structure with *a*=6.6, *b*=16.6, *c*=13.9 Å, and pore size *R*=7.61 Å. The similar pore sizes obtained indicate that the proposed strategy is feasible for analysis. To validate the proposed method of evaluation, we conducted Le Bail analysis for most of the PXRD patterns, shown in Figure 4a–c. Extracted unit cell parameters match with predicted values with an uncertainty of ±0.2 Å (Figures S18–S34). For the case of MIL‐53(Cr) in DMF (Figure 4a), two sets of reflections are observed, pointing out on the phase mixture. Since there are two representative peaks at 9.2° and 9.9°, the presence of the following phases is assumed: 1) *a*=6.6, *b*=17.3, *c*=11.6 Å, R=6.40 Å; 2) *a*=6.6, *b*=18.1, *c*=10.3 Å, R=5.40 Å. The cases for MIL‐53(Cr) in DMSO, DCM and CHCl_3_ show the representative peaks at 9.6°, 8.4° and 8.4°/10.6°, which correspond to a structure with a pore size of *R*=6.10, 7.50 and 7.50/4.70 Å, respectively. Herein, the MIL‐53(Cr)‐lp adsorbs the solvent molecules accordingly and the pores contract to some extent, forming so‐called intermediate phase. In summary, CH_3_CN (*R*=7.61 Å) and DCM (*R*=7.50 Å) tend to keep the pores open, while CHCl_3_ (*R*=7.50/4.70 Å), DMSO (*R*=6.10 Å) and DMF (*R*=6.40/5.40 Å) tend to contract the pores slightly.

To further illustrate the impact of the starting material and product of the catalysed reaction on the structural adaption, the PXRD patterns of MIL‐53(Cr)‐lp with pre‐adsorbed solvent containing substrate and/or product were measured. Here, 4’‐bromoacetophenone was selected as a model substrate for the dehalogenation reaction and transformed into acetophenone as the product. As shown in Figures 4a–c, the PXRD patterns of MIL‐53(Cr) in a specific organic solvent with substrate added were identical to the ones with sole solvent molecules adsorbed. The 5 sets of comparison indicate that the introduction of substrate molecules does not alter the framework structure. However, the change in some PXRD patterns is observed when the product molecules are generated in situ in the pores of MIL‐53(Cr). In DMF and DCM, the structures remain unchanged. In DMSO, the intensity of the peak at 8.3° increases significantly, indicating that the fraction of structure with R=7.61 Å increases. In other words, the pores are further enlarged. In CHCl_3_ and CH_3_CN, a new peak appears at 8.7° and 8.8°, respectively, thus, the pores contract in the presence of the product. This unpredictable structure deformation tendency is ascribed to the subtle differences in the host–guest and guest‐guest interactions inside the pores.

Since a catalytic reaction is a dynamic process, the decrease in substrate concentration and the increase in corresponding product concentration may also affect the pore aperture of the MOF. To verify this point, the PXRD patterns of MIL‐53(Cr)‐lp loaded with different ratios of substrate/product in CH_3_CN were also measured (Figure 4c). There are three representative peaks at 2*θ*=8.3°, 8.8° and 10.2°, with remaining positions, changing the intensity gradually with the gradually rising concentration of the product. In summary, the structural variety of the MIL‐53(Cr) is determined by a combined effect from all the molecules adsorbed in the pores, but the type of solvent plays a critical role.

### Pair Distribution Function (PDF) Analysis

**Figure 5:**
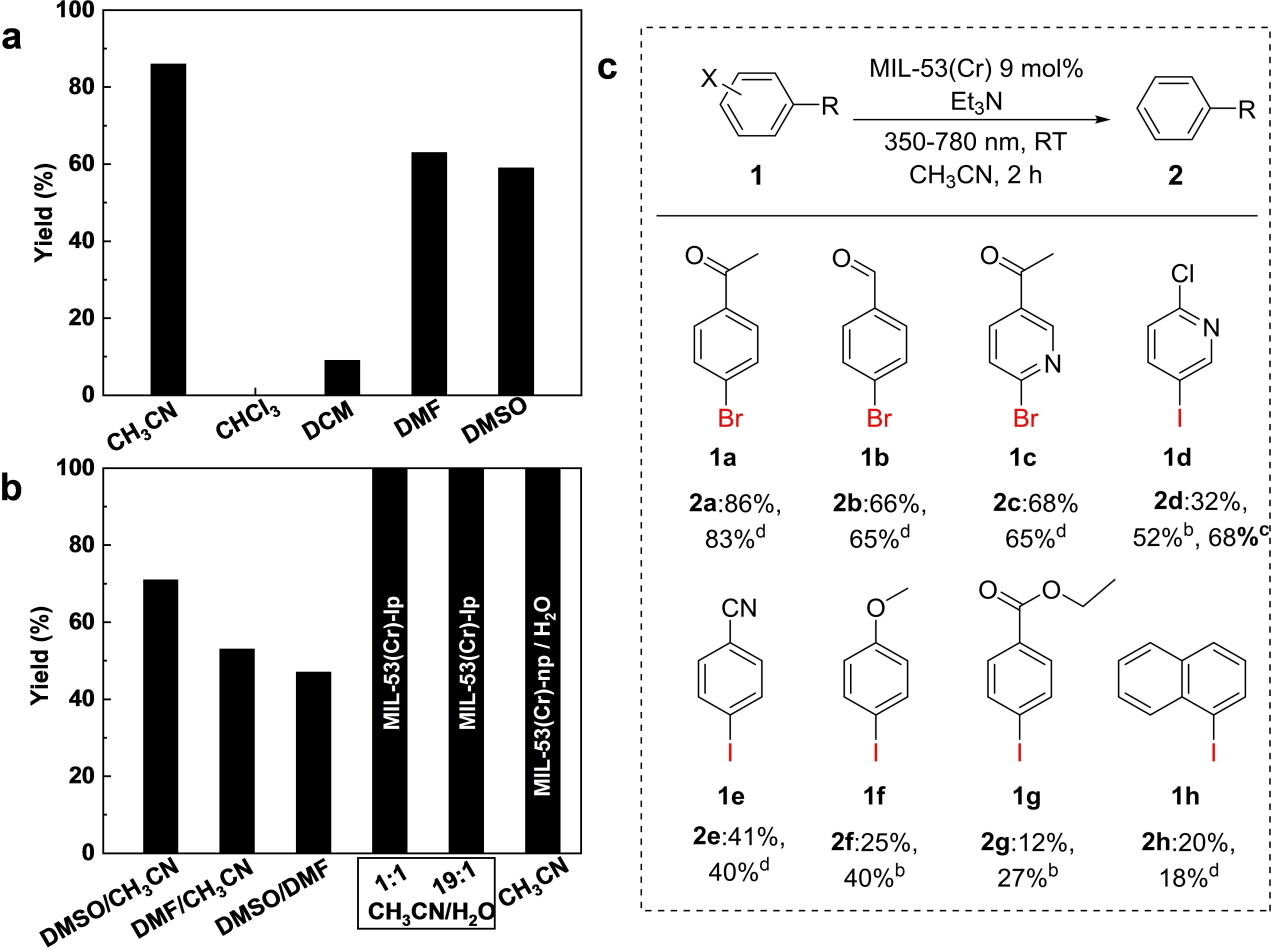
Photocatalytic study. (**a**) Photocatalytic dehalogenation of 4’‐bromoacetophenone in different organic solvents using MIL‐53(Cr)‐lp as a catalyst; (**b**) in 1 : 1 ratio of DMSO/CH_3_CN, DMF/CH_3_CN and DMSO/DMF, in 1 : 1 and 19 : 1 ratio of CH_3_CN/H_2_O using MIL‐53(Cr)‐lp as catalyst, and in CH_3_CN using MIL‐53(Cr)‐np (H_2_O) as catalyst; Yields for photocatalytic dehalogenation of aromatic halides over MIL‐53(Cr) (**c**). The yields of corresponding products are determined by ^1^H NMR spectroscopy using mesitylene as the internal standard and integral method. The spectroscopic data and NMR spectrum of the reaction mixtures and isolated products are given in the Supporting Information. Reaction conditions: substrate (0.50 mmol), MIL‐53(Cr) (9 mol %, 0.01 g), Et_3_N (1.0 mmol), dry CH_3_CN (5 mL), 25 °C, 350–780 nm, ^b^ 12 h or ^c^ 24 h, ^d^ isolated yield.

To gain insights into the local structural information, total scattering experiments[^40^, ^41^] were conducted on 10 samples, including MIL‐53(Cr)‐lp and MIL‐53(Cr)‐np (H_2_O), MIL‐53(Cr)‐lp in different solvents (DMSO, CH_3_CN, CHCl_3_, DCM and DMF) loaded with substrate **1 a** (4‐bromoacetophenone), MIL‐53(Cr)‐lp in CH_3_CN loaded with substrate **1 b** (4‐bromobenzaldehyde) and substrate **1 f** (4‐iodoanisole, see structure **1 a**, **1 b**, **1 f** in Figure 5), and MIL‐53(Cr)‐np in CH_3_CN loaded with substrate **1 a** (Figures 5d–5f, S8 and S9).

We employed PDF analysis to analyze the local order of MIL‐53(Cr) framework soaked in different solvents and reaction substrates (Figures 4d–4 f). The most intense peak at 1.97 Å is characteristic of the Cr−O bond, and the peak at *r* ~1.4 Å represents the C−C and C−O bonds. For comparison, the Cr/Cr, Cr/O and Cr/C distances were extracted from crystal structures of MIL‐53(Cr)‐lp (CCDC 797263) and MIL‐53(Cr)‐np (CCDC 797261) phases. Take the distances between Cr atoms as an example, *r*=3.41, 6.81 and 10.21 Å could be found in all PDF patterns as expected. As shown in Figure 4d, after adsorbing a tiny amount of H_2_O, new peaks, appeared at *r*=4.7, 5.9, and 6.5 Å, can be assigned to Cr/C distances. Similarly, peaks at *r*=8.4, 9.9, 10.2 and 11.8 Å representing Cr/O distances are getting shorter in MIL‐53(Cr)‐np, due to hydrogen bonding formed between the −OH groups in MIL‐53(Cr) and H_2_O molecules. Loading MIL‐53(Cr) with substrate and different solvent molecules (Figure 4e) results in the appearance of some new peaks as dotted line marked and the change of relative intensity of known peaks. All the changes indicate that the host–guest interactions between adsorbed guest molecules within the MOF framework have a critical influence on the structure reconfiguration.

#### Photocatalytic Studies of MIL‐53(Cr)

MIL‐53(Cr) was utilized as a photocatalyst for the dehalogenation reaction, which is a typical reaction for toxic pollutant degradation and water treatment. Compared with conventional semiconductors,^[42]^ MOF‐based materials, such as Zn‐PDI (PDI**=**perylene diimide)^[43]^ and MFM‐300(Cr)^29^ have been demonstrated to be efficient photocatalysts for the visible‐light‐driven reduction of aryl halides. Moreover, it is a simple heterogeneous catalytic reaction with a distinct mechanism and easy post‐processing, which is ideal as a model reaction for the further study of the influence of structure reconfiguration on the catalytic performance of MOF. The solvent effects were studied utilizing different single or mixed organic solvent systems, using 4’‐bromoacetophenone as the model substrate and triethylamine (Et_3_N) as a sacrificial agent for supplying protons. Five organic substances discussed above (CH_3_CN, DMF, DMSO CHCl_3_ and DCM) have been used as solvents.

#### Studying the Relationship between Pore Size and Catalytic Activity

As it is evident from Figure 5a, CH_3_CN shows the best performance in terms of yield (88 %), which is comparable with the reported rigid MOFs Zn‐PDI (85 %)^[43]^ and MFM‐300(Cr) (95 %),^[44]^ regardless of the reaction conditions. The yield achieved in CH_3_CN is higher than in DMF (63 %) and DMSO (59 %), a low yield was obtained for DCM (9 %), and no product was obtained using CHCl_3_ as solvent. The low yield in DCM and CHCl_3_ could be ascribed to the inherent properties of the solvents, such as low polarity solvents and low proton conduction, thus reducing or inhibiting reaction activity. Combined with the pore size of MIL‐53(Cr) in CH_3_CN (*R*=7.61 Å), DMF (*R*=6.40/5.40 Å), and DMSO (*R*=6.10 Å) obtained from PXRD analysis, it is interesting to find that the pore size seems to correlate with catalytic activity, the larger pore size in CH_3_CN results in the best performance.

To confirm this hypothesis, solvent mixtures of CH_3_CN/DMF, CH_3_CN/DMSO, and DMSO/DMF in a 1 : 1 ratio were utilized in the photocatalytic dehalogenation reaction (Figure 5b), controlling the yield and the framework state (Figure S10a). Interestingly, the decrease in yield is consistent with the decreasing pore size: 71 % in CH_3_CN/DMSO (*R*=7.61/5.71 Å) >53 % in CH_3_CN/DMF (*R*=6.65/5.40 Å) >47 % in DMSO/DMF (*R*=6.40/5.40 Å). This tendency confirms that the pore size critically impacts the catalytic activity. For the comparable pore sizes, as in the case of DMF and DMSO/DMF (1 : 1) (*R*=6.40/5.40 Å), the yields obtained are slightly different due to the differences in the chemical nature of the solvents. Furthermore, the photocatalytic activity could thus be adjusted by different combination of solvents, such as combining a favourable and unfavourable solvent (DMF/CHCl_3_, DMSO/DCM, CH_3_CN/DCM, DMF/DCM, and CH_3_CN/CHCl_3_) in a 1 : 1 ratio (Figure S11a), and two pairs of mixed solvent systems, CH_3_CN/CHCl_3_ and CH_3_CN/DCM mixtures in different ratios (Figures S11b and S11c). The corresponding PXRD analysis were given in Figures S12 and S13 (see the “Discussion section”).

#### Variation of Substrates

The performance of MOF in this photocatalytic dehalogenation reaction was further studied for 8 different substrates, including 3 bromides and 5 iodides (Figure 5d), using CH_3_CN as solvent. The diversity of substrates confirms the universality of MIL‐53(Cr) as a photocatalyst and the precision of the proposed mechanism.^[45]^ When comparing the catalytic performance with a non‐porous copper catalyst,^[46]^ the substrates **1 a** (yield of 86 %) and **1 b** (yield of 66 %) give similar dehalogenation yields as reportedin literature (95 % and 63 %, respectively), however, yields for substrates **1 i** (yield of 19 %) and **1 j** (yield of 20 %) (Supplementary spectroscopic data) are far lower than reported values (90 % and 85 %, respectively). Herein, another concern that needs to be addressed is whether the molecular size of the substrates affects the catalytic activity of MIL‐53(Cr). To confirm this, the PXRD patterns of MIL‐53(Cr)‐lp with different substrates (**1 a**, **1 f**, **1 g**, **1 h** in Figure 5) in combination with CH_3_CN (Figure S14) were measured. The patterns slightly differ from each other, resulting from different pore apertures with varying degrees of shrinkage. This finding indicates that different substrates impact the pore size of MIL‐53(Cr), which may result in differences in catalytic activity. The PDFs of MIL‐53(Cr)‐lp loaded with different substrates in CH_3_CN were also analysed (Figure 4f). Since the chemical structures of **1 a** and **1 b** are similar, the host–guest interaction with the MOF skeleton should be similar, and indeed, the PDFs are thus almost the same. When **1 f** was loaded with the substrate, not only the intensity differences were observed, but the most intense peak at *r*=1.97 Å (Cr−O bond) decreased dramatically, which is probably ascribed to the electron‐rich iodine‐containing substrate, further indicating that different substrates cause specific host–guest interactions with the MOF framework.

Furthermore, control experiments were conducted to gain additional insights (Table S3). As listed in entry 2, light plays a critical role, and no product was detected under dark conditions, as expected. When a powdered mixture of Cr(NO_3_)_3_ ⋅ 9H_2_O and H_2_BDC, the building blocks for synthesizing MIL‐53(Cr), were used as the photocatalyst, a low yield of product was observed (2 %, entry 3), which indicates that the framework structure of MIL‐53(Cr) is crucial. Low yields were obtained when Et_3_N was not present (17 %, entry 5) or replaced by Na_2_SO_3_ (15 %, entry 4). In addition, the reaction rate decreases as a function of time (Figure S11d), which was ascribed to the kinetic process of adsorption of substrate molecules and desorption of product molecules within the catalyst, indicating that it is a dynamic process. Moreover, the stability and robustness of MIL‐53(Cr) were confirmed by PXRD and adsorption analysis on used/recycled MIL‐53(Cr) after photocatalytic reactions (Figure S15).

#### Studying the Role of H_2_O in Photocatalysis

Another reference experiment is done using the narrow pore phase of MIL‐53(Cr) with a small amount of H_2_O adsorbed in the pores. Interestingly, the MIL‐53(Cr)‐np (H_2_O) as catalyst results in a boosted catalytic efficiency in CH_3_CN (≥99 %, Figure 5b), which is even higher than using MIL‐53(Cr)‐lp as catalyst in pure CH_3_CN (86 %). Moreover, photocatalytic reactions using MIL‐53(Cr)‐lp as a catalyst in 1 : 1 and 19 : 1 CH_3_CN/H_2_O solvent mixtures were conducted and ≥99 % yields were achieved in both cases, which indicates that a tiny amount of H_2_O plays a critical role here, either adsorbed in the pores of MOF or as impurity in the solvent. Moreover, MIL‐53(Cr)‐np could be recycled and retains excellent catalytic activity over at least five cycles (Figure S16). To clarify the role of H_2_O in the photocatalytic reaction, the PXRD patterns of MIL‐53(Cr)‐np (H_2_O) in CH_3_CN without and with substrate added were measured (Figure S17). By comparing with MIL‐53(Cr)‐lp in CH_3_CN (*R*=7.61 Å), MIL‐53(Cr)‐np (H_2_O) in CH_3_CN has 2 representative peaks at 2*θ*=9.4° and 10.9° with corresponding apertures of *R*=6.20 and *R*=4.40 Å. However, when the substrate is added, MIL‐53(Cr)‐np (H_2_O) shows only one phase with a larger aperture (*R*=7.61 Å), which indicates that in the case of water, the substrate molecules determine the framework state and not the solvent as demonstrated above. The so enlarged pore size contributes to the promoted catalytic efficiency. Also, the strong solvent‐substrate‐framework interaction via hydrogen bonds through H_2_O obviously plays a crucial role here. H_2_O could also act as an excellent proton donor together with Et_3_N^[47]^ positively influencing the yield.

To confirm the influence of a trace amount of H_2_O in CH_3_CN on the MOF structure, the total scattering data on MIL‐53(Cr)‐lp and MIL‐53(Cr)‐np (H_2_O) in CH_3_CN with substrate were recorded. After loading the solvent and substrate molecules, the characteristic host–guest interactions were confirmed by comparing them with the PDF analyses in Figure S9. With the trace amount of H_2_O inside the pores, the intensity of peaks at *r*=1.2, 5.9, 6.5, 7.7 Å is stronger, the peaks at *r*=5.5, 6.1, 10.6 Å are weaker, and the peak at 2.9 Å splits into two peaks at 2.8 Å and 3.0 Å. These differences were ascribed to the stronger interaction between H_2_O and MOF, further confirming the preferred adsorption of H_2_O over CH_3_CN molecules in MIL‐53(Cr). In summary, the structures of the adsorbed molecules, either solvent, substrate molecules or tiny amount of water molecules, determine the type and strength of the host–guest interaction with the MOF skeleton, which results in different structure deformations of the flexible MOF.

## Conclusion

The inherent cavities and dynamic behavior of a flexible MOF make it an excellent candidate for the development of artificial switchable catalysts, while the complexity, when applied to a solution catalytic reaction, leads to a fundamental intellectual challenge clarifying the complex interplay of factors affecting the catalyst activity and selectivity. Herein, the breathable MIL‐53(Cr) framework is demonstrated to be a promising photocatalyst with high reducing potentials, which is active in photocatalytic dehalogenation reaction. The adaptive structural flexibility and dynamic pore architecture of MIL‐53(Cr) in photocatalysis were elucidated by PXRD and PDF analysis and upon guest molecules adsorption, specifically accounting for the impact of solvent versus substrate and product molecules, respectively. The catalytic efficiency is influenced jointly by a number of factors with varying impacts: 1) the dynamic structure deformation of MIL‐53(Cr), to be specific, the aperture size/volume of MIL‐53(Cr), 2) the electronic structure of np/lp configurations of MIL‐53(Cr), 3) the inherent properties of the solvents and solvent effects, 4) the water content of the solvent, 5) substrate effects (steric, electronic, functional groups) and 6) the resulting solvent‐substrate‐framework host–guest and guest‐guest interactions.

Future research should focus on developing advanced frameworks with tailored breathing mechanisms for specific reactions, enhancing stability under operational conditions, and exploring multi‐stimuli responsive systems. Integrating computational modeling with experimental studies could further enable the precise prediction of framework behavior, facilitating the design of “smart” catalysts with tunable selectivity and activity. Ultimately, flexible MOFs could play a pivotal role in sustainable chemical processes, bridging the gap between homogeneous and heterogeneous catalysis while contributing to the advancement of artificial enzyme technologies.

## Supporting Information

Materials, instrumentation, characterization in details, supporting Figures and Tables, NMR spectra (PDF). Raw data related to the DFT simulations is available from NOMAD repository. The authors have cited additional references within the Supporting Information.

## Conflict of Interests

The authors declare no conflict of interest.

1

## Supporting information

As a service to our authors and readers, this journal provides supporting information supplied by the authors. Such materials are peer reviewed and may be re‐organized for online delivery, but are not copy‐edited or typeset. Technical support issues arising from supporting information (other than missing files) should be addressed to the authors.

Supporting Information

## Data Availability

The data that support the findings of this study are available from the corresponding author upon reasonable request.
