# Supplementary material for: Photocatalytic Dehalogenation of Aryl Halides Mediated by the Flexible Metal–Organic Framework MIL‐53(Cr)
Source: Angew Chem Int Ed Engl. 2025 Jan 13;64(13):e202422776. doi: 10.1002/anie.202422776 (PMC11933537; doi:10.1002/anie.202422776)
Supplement: Supplementary file 1 — Supporting Information [file ANIE-64-e202422776-s001.pdf]

Angewandte Chemie
Eine Zeitschrift der Gesellschaft Deutscher Chemiker

Supporting Information

Photocatalytic Dehalogenation of Aryl Halides Mediated by the Flexible Metal–Organic Framework MIL-53(Cr)

T. Luo, H. S. Jeppesen, A. Schoekel, N. Bönisch, F. Xu, R. Zhuang, Q. Huang, I. Senkovska, V. Bon, T. Heine, A. Kuc, S. Kaskel**

Supplementary Information

Photocatalytic Dehalogenation of Aryl Halides Mediated by the Flexible Metal–Organic Framework MIL-53(Cr)

Tian Luo[1], Henrik S. Jeppesen[2], Alexander Schoekel[2], Nadine Bönisch[1], Fei Xu[3], Rong Zhuang[3], Qiang Huang[1], Irena Senkovska[1], Volodymyr Bon[1]* Thomas Heine[4,5,6], Agnieszka Kuc[4,5], and Stefan Kaskel[1]*

1. Department of Inorganic Chemistry, Dresden University of Technology, Bergstr. 66, 01069 Dresden, Germany

2. DESY (Deutsches Elektronensynchrotron), FS-PETRA-D, P02.1, Notkestr. 85, 22607 Hamburg, Germany.

3. School of Materials, Northwestern Polytechnical University, 710060, Xi'An, China.

4. Helmholtz-Zentrum Dresden-Rossendorf, HZDR, Bautzner Landstr. 400, 01328 Dresden, Germany

5. Center for Advanced Systems Understanding, CASUS, Conrad-Schiedt-Straße 20, 02826 Görlitz, Germany

6. Yonsei University and ibs-cnm, Seodaemun-gu, Seoul 120-749, Republic of Korea

Supplementary information

1. Materials.

$Cr(NO_3)_3 \cdot 9H_2O$ (>98%), terephthalic acid (H_2bdc) and *N,N*-dimethylformamide (DMF, 99.8%, extra dry over molecular sieve), and dimethyl sulfoxide (dry DMSO, 99.7+%) were purchased from Acros Organics. Acetonitrile (CH_3CN, 99.9%, extra dry over molecular sieve), anhydrous chloroform ($CHCl_3$, ≥99%), dichloromethane (DCM, inhibitor-free, dried over molecular sieve), hydrofluoric acid (HF, 40%) were purchased from Sigma-Aldrich. Sodium sulphite (Na_2SO_3) was purchased from Grüssing GmbH and triethylamine (Et_3N, ≥99.5 %) from Carl Roth GmbH, and dried with molecular sieves before usage. All other chemicals and reagents were used as received without further purification.

2. Methods and instrumentation.

Powder X-ray Diffraction

Powder X-ray diffraction (PXRD) patterns were collected on an X-ray powder diffractometer (STOE transmission diffractometer system) using Cu-Kα1 radiation (λ = 1.54059 Å), operated at 40 kV and 30 mA in 2θ range of 2-50° at a scan speed of 2.4 °/min. Each sample was measured three times to get a sum pattern.

TGA

Thermogravimetric analysis (TGA) was performed at a heating rate of 10 °C/min from 25 to 800 °C under air flow using STA 409 PC (NETZSCH).

CHNS

Elemental analysis for C, H and N content of MIL-53(Cr) were carried out using CHNS elemental analysers equipped with a vario MICRO cube elementar.

FTIR

Fourier-Transform Infrared Spectroscopy (FTIR) spectra were recorded using a Bruker VERTEX 70 spectrophotometer using the ATR technique in the range of 4000-600 cm^{-1}.

SEM

Scanning electron microscope (SEM) images were taken with a HITACHI SU8020 microscope at an acceleration voltage of 2.0 kV and a working distance of 8.1 mm. The samples were placed on a carbon sample holder and coated with Au to enhance the conductivity.

Gas adsorption isotherms

Nitrogen physisorption was measured using a Quadrasorb (SI, 3P instruments) instrument in a pressure range of 10^{-3} to 1 bar at 77 K. MIL-53(Cr) desolvated samples (around 40-60 mg) were previously activated at 120 °C under vacuum (up to 10^{-3} bar) for 24 h. MIL-53(Cr)-np sample with H_2O adsorbed was measured directly without activation. Brunauer-Emmett-Teller (BET) surface areas were calculated using AsiQwin software from the linearized BET plot within the p/p_0 pressure range of 0.05–0.25 so that the linear model fit had an R2 value greater than 0.999.

UV-Vis DRS

Solid-state UV-Vis diffuse reflectance spectroscopy (DRS) data was collected on a UV-Vis spectrophotometer (VARIAN, 4000) over a wavelength range of 200-800 nm equipped with an integrating sphere using $BaSO_4$ as reference. The optical band gaps were calculated under the hypothesis of Kubelka-Munk equation and Tauc plot.

VB-XPS

Valence band X-ray photoelectron spectroscopy (VB-XPS) spectra of MIL-53(Cr)-lp and MIL-53(Cr)-np were recorded with Shimadzu AXIS $SUPRA^+$ spectrometer using Al-Kα radiation (hν = 1486.6 eV) at 14.6 kV and 13.5 mA.

NMR

^{1}H nuclear magnetic resonance (NMR) and ^{13}C NMR spectra of the reaction solutions were acquired on a Bruker Avance III HD 300 MHz spectrometer using DMSO-d_6 or $CDCl_3$ as the solvent at 25 °C. The data were processed with the software MestReNova.

Qualitative phase analysis

PXRD patterns, measured on MIL-53(Cr) soaked in acetonitrile, dichloromethane, chloroform, DMF, DMSO, and corresponding solutions of starting materials, product of the reaction, and their mixture were analysed by Le Bail method. The unit cell parameters for PXRD patterns containing single phase were obtained using indexing procedure of DICVOL91 and refined using Fullprof software. In cases where two phases are observed, the reflections of the known phase were omitted from the indexing and the Le Bail fit was conducted using Fullprof software. The resulted plots and unit cell parameters are provided in **Figures S18 - S34**.

Crystal engineering

Structural models were generated using Materials Studio 5.0 software (Accelrys, 2008). PXRD patterns containing single **lp** and **np** phases were indexed using the Reflex tool, and the structures were optimized by the Forcite Geometry optimization algorithm using Universal Force Field (UFF). Intermediate structures were simulated using **lp** or **np** phases as initial models by incrementally increasing ***b*** unit cell length within the range of 16 – 20 Å and simultaneously decreasing ***c*** unit cell length in the range of 14.5 – 8 Å. The length of ***a*** axis was fixed in all structures at 6.6 Å. Subsequently, all models were subjected to geometry optimization using UFF. The unit cell parameters of all simulated structures are given in **Table S1**.

Pore size distribution

The pore size distribution was calculated using Zeo++ software[1]. The structural models obtained from the PXRD analysis were used for the calculations. The hypothetical spherical probe molecule with an atomic radius of 1.2 Å (equivalent to a helium atom) was used. Default parameters with 100 bins, each 0.1 Å in size, were used for calculation in the range between 0 and 100 Å. The results are summarized in the **Figure S1**.

Density functional theory simulations. All systems were fully relaxed (atomic positions and lattice parameters; unless otherwise stated) employing density functional theory with hybrid PBE0 functions[2] and Grimme's D3 dispersion interaction correction[3] as implemented in Crystal23[4]. The fully optimized lattice parameters are as follows: a = 17.120 Å, b = 12.418 Å, c = 6.816 Å, α = 89.99°, β = 91.44°, γ = 90.00° for MIL-53(Cr)-lp and a = 21.441 Å, b = 5.626 Å, c = 6.842 Å, α = 89.86°, β = 117.65°, γ = 90.00° for MIL-53(Cr)-np. The hybrid functional is necessary to properly distributes electrons in these spin-polarized simulations, with spin magnetic moment on Ni atoms and their surroundings. We considered antiferromagnetic ordering of Ni atoms within each chain and between

chains, which is the lowest energy state. We used POB-TZVP basis sets[5] and Monkhorst-Pack[6] Γ-centered grid with 6×6×12 and 4×12×12 dimensions for lp and np crystal structures, respectively. The electronic band structures and band gaps were calculated using HSE06 hybrid functional[7],[8], which describes these properties better than PBE0.

3. Experimental section.

Synthesis of MIL-53(Cr), [Cr(OH)(bdc)]. MIL-53(Cr) was synthesized by hydrothermal reaction of chromium salt and terephthalic acid (H_2bdc) in acidic (HF) water in the molar ratio of 1:1:2.4:284 at 220 ºC.[9] To be specific, $Cr(NO_3)_3 \cdot 9H_2O$ (1.59 g, 4 mmol) and H_2bdc (0.66 g, 4 mmol) were added to H_2O (20 mL) in a 50 mL Teflon liner of an autoclave. After being acidified by HF (40%, 0.15 mL), the suspension was placed in an ultra-sonicator for 10 minutes and then transferred into an oven and heated at 220 °C for 3 days. After natural cooling down and filtration, the green solid was collected and transferred into fresh DMF (100 mL) under stirring at 80 ºC for 6 h to dissolve the unreacted linker. After filtration, the obtained solid was further washed with fresh DMF three times and transferred into ethanol (100 mL) under stirring at 70 ºC for 6 h to replace free DMF molecules in the pores. Being filtered off, washed with fresh ethanol, and dried at 80 ºC overnight, the resulting solid was calcined at 320 ºC in air for 24 h to further remove the residual DMF molecules in the MOF pores. MIL-53(Cr)-lp was collected and identified by powder X-ray diffraction (PXRD) analysis. For storage and further experiments, MIL-53(Cr)-lp was kept in the glovebox to avoid contact with moisture in the air. CHN analysis (% cal./found): $[Cr(OH)(O_2C\text{-}C_6H_4\text{-}CO_2)](H_2O)_n$ (C 38.3/38.5, H 2.79/2.25, N 0.0/0.0). Selected ATR-IR (**Figure S4b**): ν /cm^{-1}: 3613(w), 3365(w), 1621(m), 1500(s), 1525(s), 1435(m), 1379(vs), 1318(m), 1290(m), 1254(w), 1156(w), 1103(w), 1070(w), 1015(m), 917(w), 885(w), 831(m), 785(w), 747(s).

Sample preparation for PXRD analysis. MIL-53(Cr)-lp sample was prepared with the aid of a specific self-made sealed sample holder in the glovebox to prevent the adsorption of water from the air[9]. For the MOF samples loaded with different solvent/substrate/product molecules, MIL-53(Cr)-lp (0.01g), substrate (0.5 mmol, if in solid) and product (at a varied amount as noted) were first weighed, added in vials, and sealed in the glovebox. Dry solvent (5 mL), substrate (if in liquid) and product (if in liquid) were injected into the vials, which were further sonicated for 5 minutes. The resulting suspension was left for 24 h to reach adsorption equilibrium. The supernatant was removed with a syringe, and the wet samples were dabbed with filter paper and sealed in the self-made sample holder instantly before PXRD measurement.

Photocatalytic reaction. In a typical photocatalytic reaction, MIL-53(Cr)-lp powder (0.01g, 10 mol%) and substrate (0.5 mmol, if in solid) were added into a round bottom flask (50 mL) in the glovebox and sealed with a rubber septum. Dry solvent (5 mL), dry Et_3N (1 mmol, 0.15 mL), and substrate (if in liquid) were added into the flask with the aid of Schlenk line. After sonication for 5 minutes, the above suspension was heated to 25.0 °C with the aid of a water bath. The 300 W Xe lamp (Zhongjiaojinyuan Co., Ltd) in the wavelength range of 350-780nm was turned on to trigger the photoreaction for a defined period of time, and the distance between the lamp and reaction solution was fixed to around 5.0 cm. Upon completion, the MOF catalyst was recycled by centrifugal separation, washed with ethanol (10 mL × 3), and then dried at 120 ºC under vacuum for 24 h. The reaction mixture (0.1 mL) was mixed in DMSO-*d6* or $CDCl_3$ (0.5 mL) and analyzed by ^{1}H NMR spectroscopy using 1,3,5-trimethylbenzene as the internal standard. For the workup of the comparison experiment using CH_3CN and H_2O mixture as solvent, the reaction mixture was transferred into a separating funnel and extracted with dichloromethane (DCM, 3 ×10 mL). The combined organic layers were analyzed by ^{1}H NMR spectroscopy.

PDF analysis. X-ray total scattering data were obtained at beamline P02.1 of PETRA III, operated by DESY (Deutsches Elektronen Synchrotron) in Hamburg, Germany[10] using a Varex XRD 4343CT detector (2880 × 2880 pixels, 150 × 150 μm^2 pixel size), using synchrotron radiation with an energy of E = 60 keV and wavelength of λ = 0.20735 Å at room temperature. All samples were measured for 30 minutes at a sample detector distance of 300 mm, resulting in a quarter-ring configuration. MIL-53(Cr)-lp was obtained under dynamic vacuum at 120 ºC overnight and stored in a glovebox, MIL-53(Cr)-np formed after leaving MIL-53(Cr)-lp in air overnight. The solvent and substrate-loaded samples were prepared for PXRD in the same way as described above. To be specific, MIL-53(Cr)-lp (0.01g) and substrate (0.5 mmol, if in solid) were first weighed, placed into vials, and sealed in the glovebox. Dry solvent (5 mL) and substrate (if in liquid) were injected into the vials, which were further sonicated for 5 minutes. The resulting suspension was left for 24 h to reach diffusion equalization. The supernatant was then removed with a syringe, dried under a weak vacuum and transferred to the glovebox. The solid powder was ground to a fine powder and added into a special glass capillary 0.8 mm in diameter and then sealed with flame. The PXRD patterns were first obtained from integrated data using the DAWN software[11] (**Figure S8**). xPDFsuite based on PDFgetX3 was used to do Fourier-transformation of the raw data to generate the G(r).[12] A Q_{max} of 20.7 Å^{-1} was used for the transformation (**Figures 4d-4f** and **S9**).

Supplementary Figures

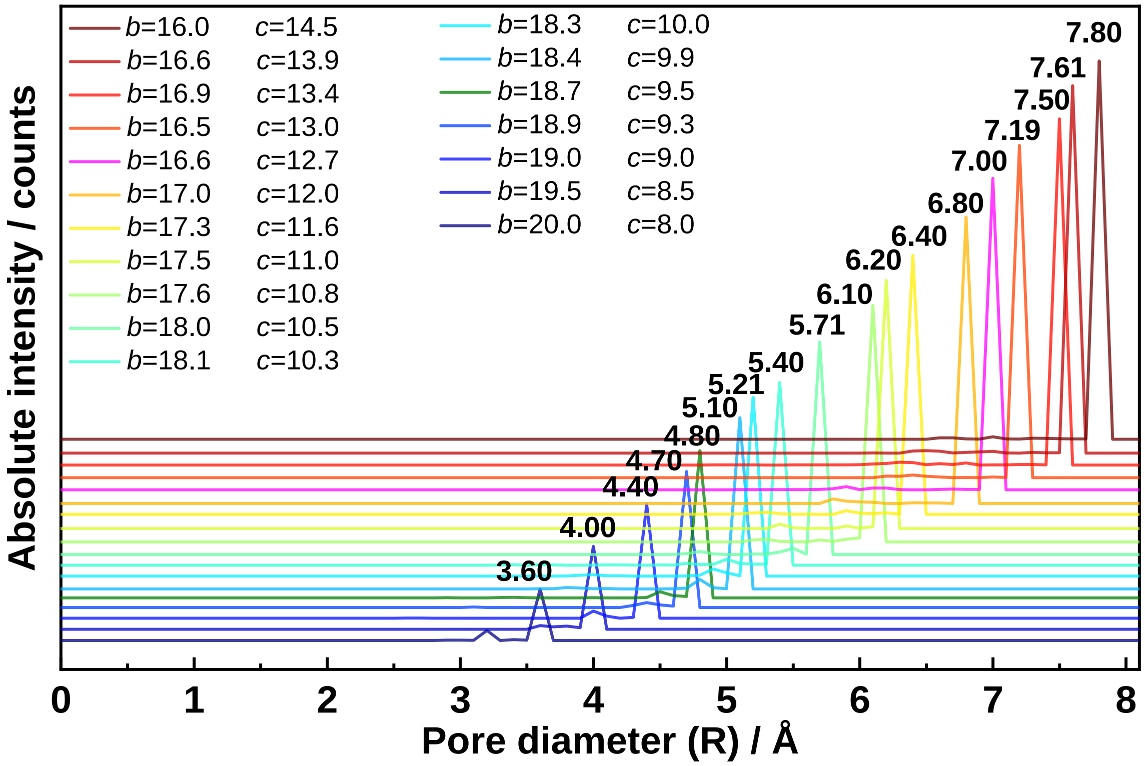

Figure S1. The pore size distribution of the simulated models of MIL-53(Cr) with cell parameter noted.

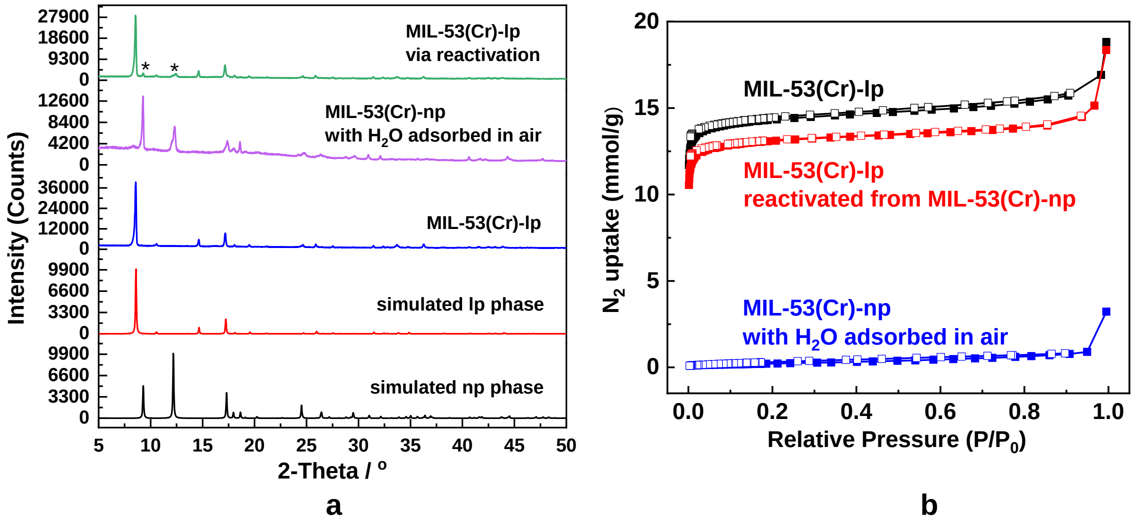

Figure S2. Physical characterizations of MIL-53(Cr). (**a**) The PXRD patterns of MIL-53(Cr): MIL-53(Cr)-lp (blue curve), MIL-53(Cr)-np obtained after exposure of MIL-53(Cr)-lp to air for 10 min (purple curve) and reactivated at 100 °C under vacuum overnight (green curve). The structure information for simulated large pore phase: space group *Imma*, *a* = 6.812 Å, *b* = 16.733 Å, *c* = 13.038 Å and *α* = β = *γ* = 90 °, for simulated narrow pore phase: space group *Cc*, *a* = 6.782 Å, *b* = 19.685 Å,

c = 7.849 Å and $\alpha = \gamma$ = 90 °, β = 104.90 (the reflections assign to the impurities are labelled with asterisks); (**b**) N_2 adsorption/desorption isotherms of MIL-53(Cr)-lp (black), MIL-53(Cr)-np obtained by exposing MIL-53(Cr)-lp sample to air for 10 min (blue) and after reactivaion at 100 ^{o}C under vacuum overnight (red).

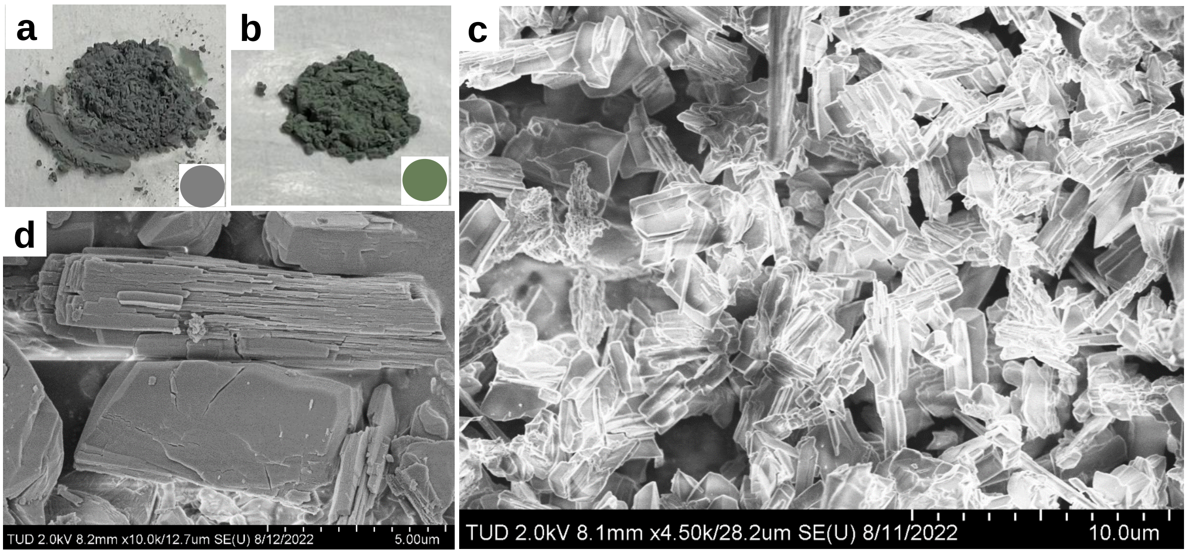

Figure S3. The morphology of MIL-53(Cr). (**a**) The image of MIL-53(Cr)-lp; (**b**) The image of MIL-53(Cr)-np with H_2O adsorbed in air; (**c** and **d**) The SEM images of MIL-53(Cr)-np in 10 and 5 um scale, respectively.

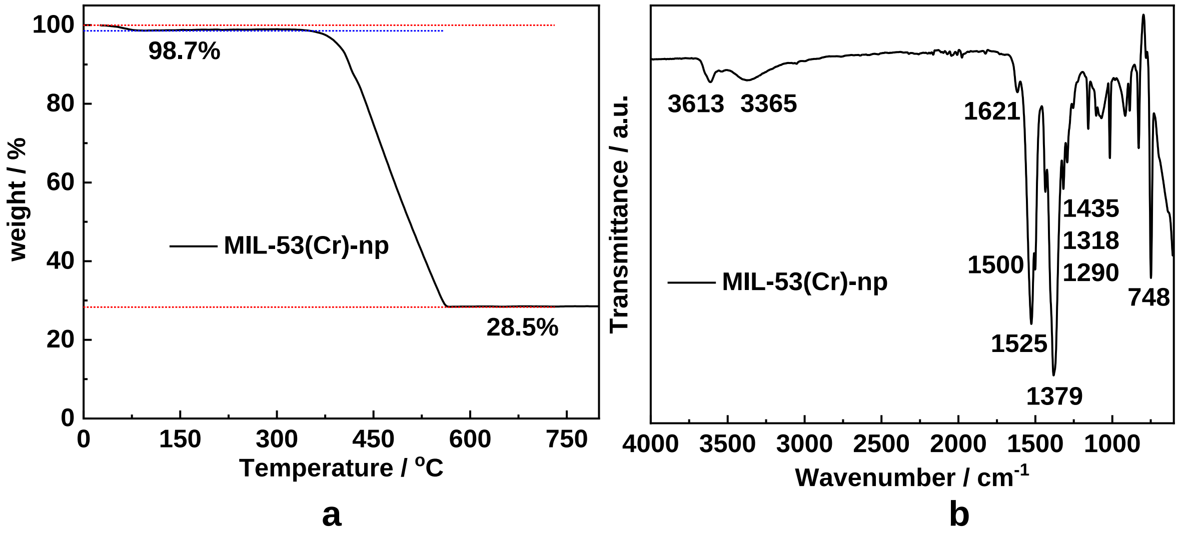

Figure S4. (**a**)The TGA plot of MIL-53(Cr)-np with H_2O adsorbed from air; (**b**) the IR spectrum of MIL-53(Cr)-np with H_2O adsorbed from air.

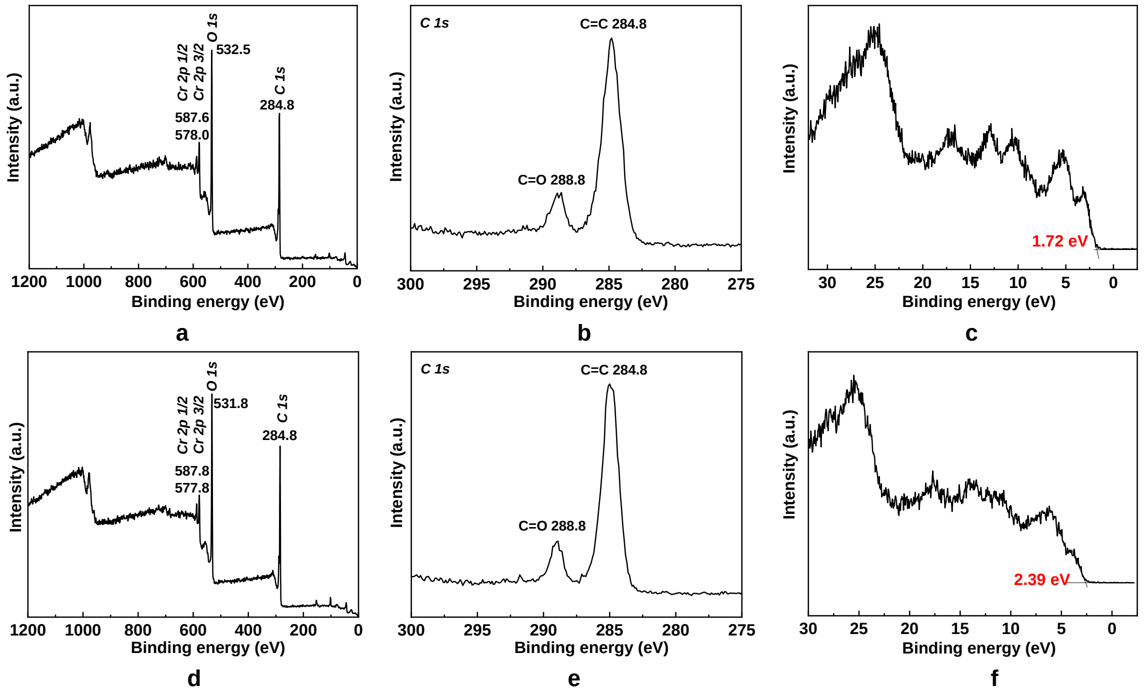

Figure S5. XPS wide spectra, high-resolution C 1s and VB-XPS of MIL-53(Cr)-lp (**a**, **b**, **c**) and MIL-53(Cr)-np (**d**, **e**, **f**).

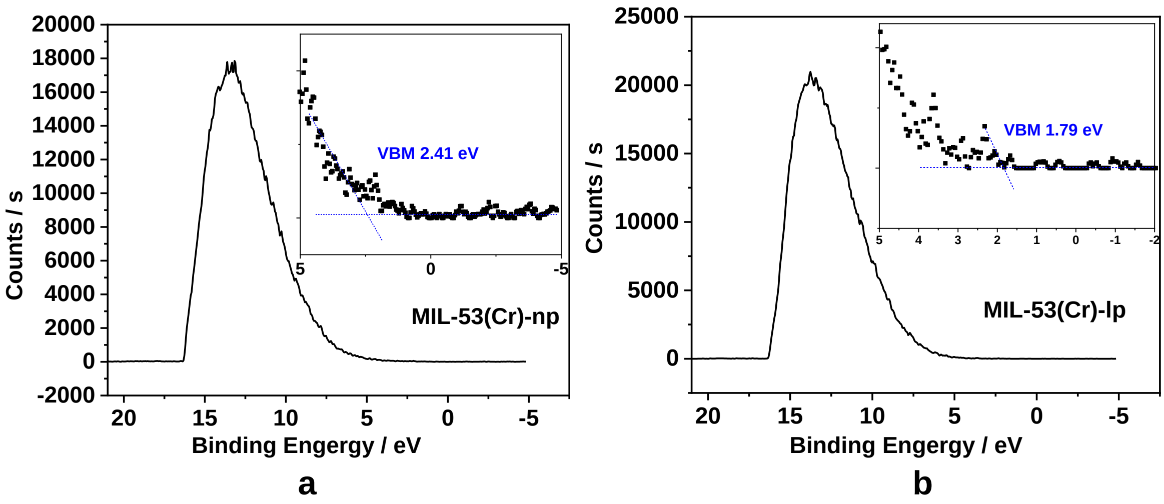

Figure S6. UPS spectra of MIL-53(Cr)-lp and MIL-53(Cr)-np measured by He I ($h\nu$ = 21.22 eV) at 0 V.

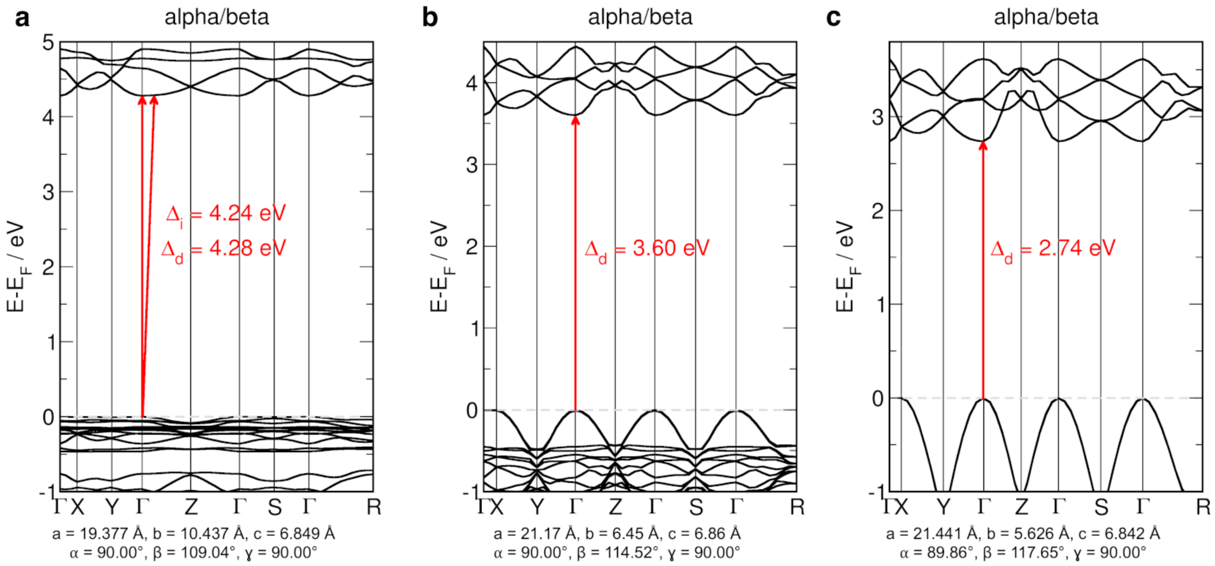

Figure S7. Band structures of MIL-53(Cr)-np. (**a**) MIL-53(Cr)-np phase experimentally observed by soaking in DMSO (**Figure** S33) with optimized atomic positions, but without optimization of the unit cell; (**b**) MIL-53(Cr)-np phase[2] with optimized atomic positions, but without optimization of the unit cell; (**c**) MIL-53(Cr)-np phase[2] with optimized atomic positions and with optimization of unit cell.

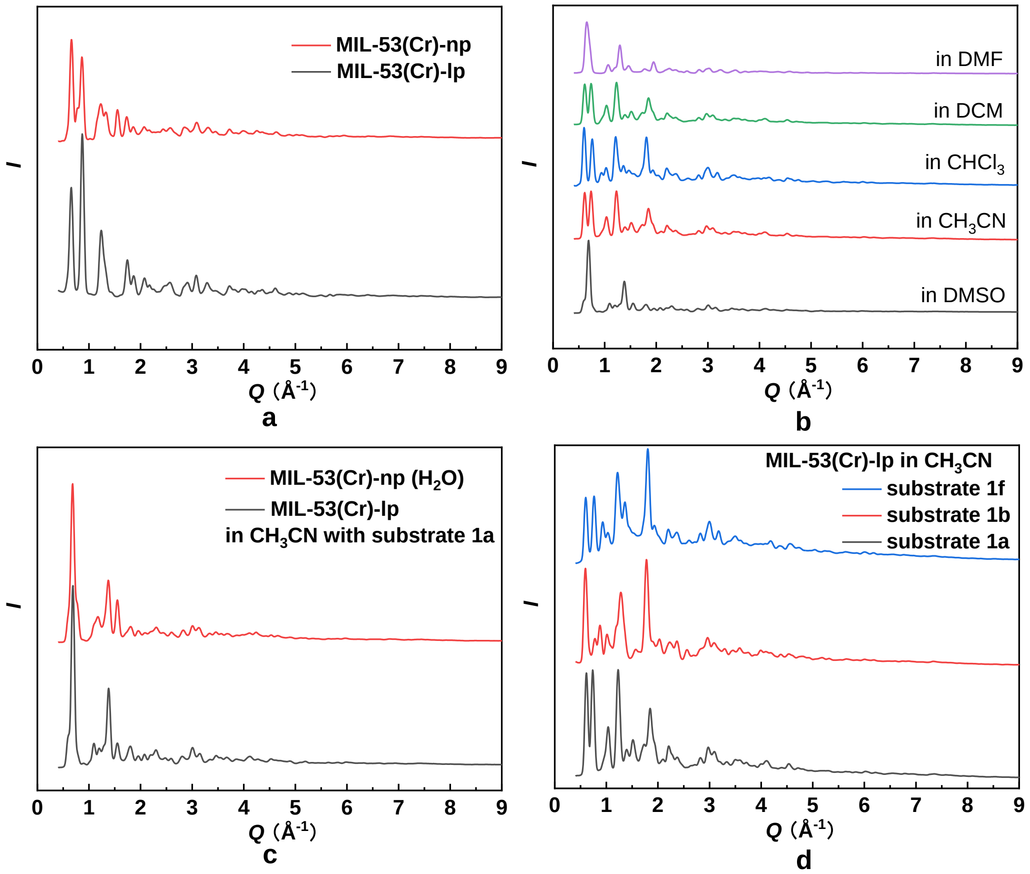

Figure S8. PXRD patterns transformed from synchrotron data obtained at DESY P02.1 (λ = 0.20735 Å). The PXRD patterns of (**a**) MIL-53(Cr) at the large pore and narrow pore phases; (**b**) MIL-53(Cr)-lp loaded with substrate **1a** in DMSO, CH_3CH, $CHCl_3$, DCM and DMF, respectively; (**c**) MIL-53(Cr)-

lp and MIL-53(Cr)-np (H_2O) loaded with substrate **1a** in CH_3CN; (**d**) MIL-53(Cr)-lp in CH_3CN loaded with substrate **1a**, **1b** and **1f**, respectively.

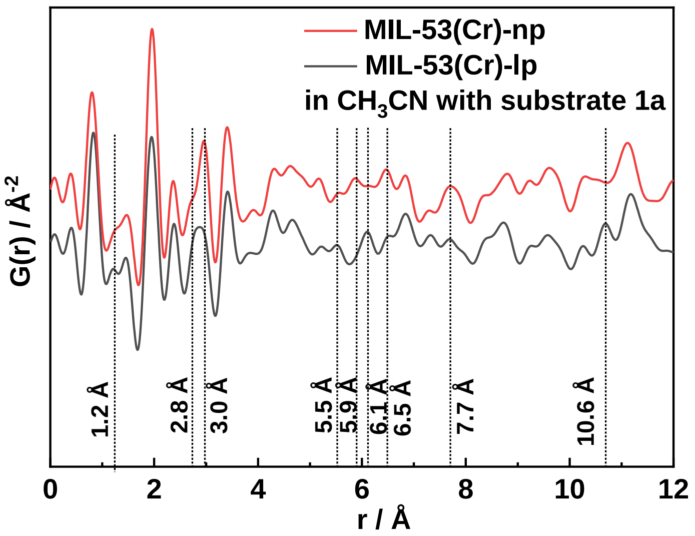

Figure S9. PDF analysis: the G(r) curves of MIL-53(Cr)-lp and MIL-53(Cr)-np loaded with substrate **1a** in CH_3CN.

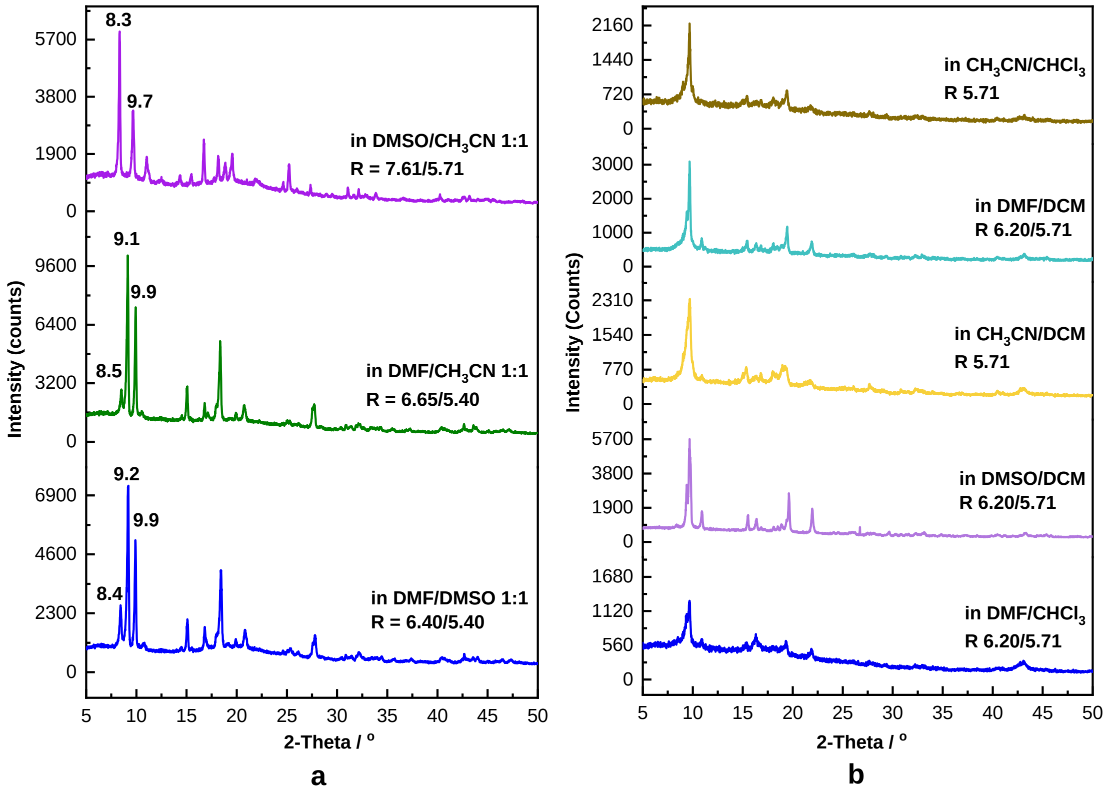

Figure S10. The PXRD patterns of MIL-53(Cr)-lp in 1:1 ratio of different organic solvents.

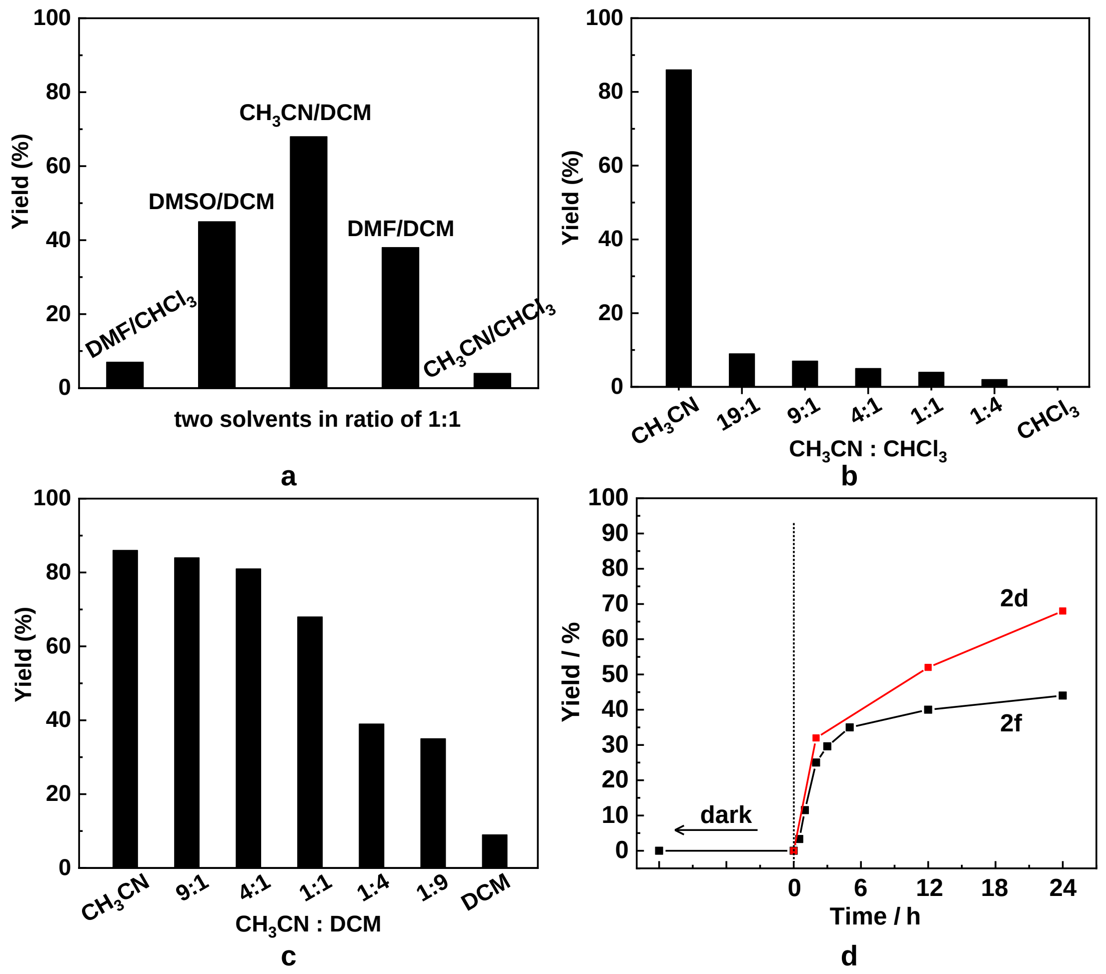

Figure S11. Photocatalytic study. Photocatalytic dehalogenation of 4'-bromoacetophenone in 1:1 ratio of solvent mixtures (**a**); in different ratios of $CH_3CN/CHCl_3$ mixtures (**b**); in different ratios of CH_3CN/DCM mixtures (**c**); Yields of product 2d and 2f with time increasing (**d**). Reaction conditions for a typical experiment: substrate (0.50 mmol), MIL-53(Cr) (9 mol%, 0.01 g), Et_3N (1.0 mmol), organic solvent (5 mL in total), 25 ºC, 350-780 nm, light irradiation for 2 h.

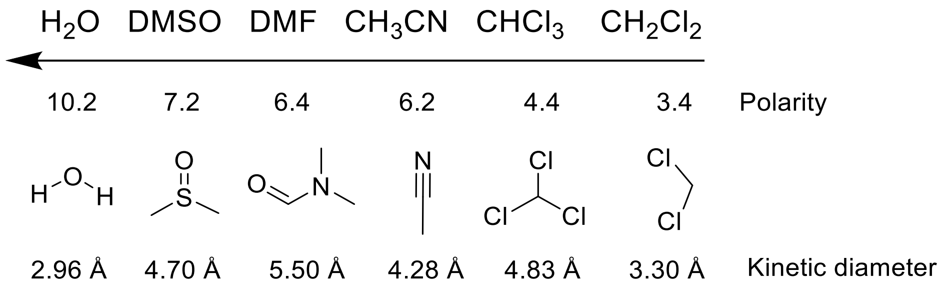

Figure S12. The polarity of different solvents (relative values).[13], [14]

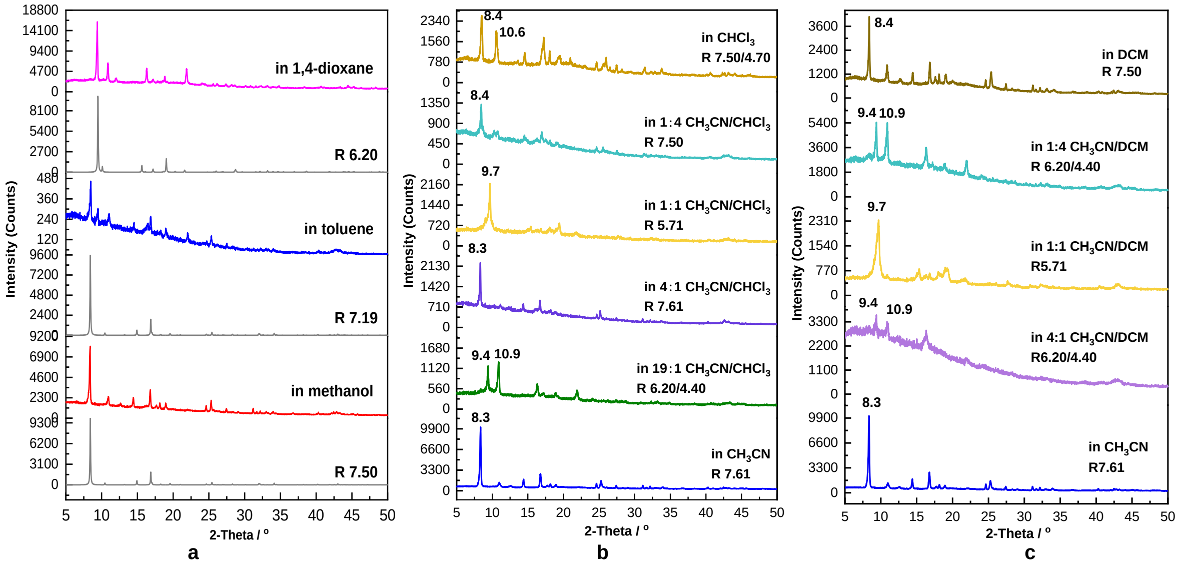

Figure S13. The PXRD patterns of MIL-53(Cr)-lp in more common solvents including methanol, toluene and 1,4-dioxane (**a**); in different ratios of $CH_3CN/CHCl_3$ mixtures (**b**) and CH_3CN/DCM mixtures (**c**) with model substrate 4'-bromoacetophenone added.

Discussion section for adjusting photocatalytic activity.

Since the catalytic activity is related to the pore size and solvent properties, as demonstrated above, it can be finely controlled by solvent. In order to clarify which factor is predominant, a range of solvent mixtures were chosen for the photocatalytic reaction. First, five solvent mixtures (DMF/$CHCl_3$, DMSO/DCM, CH_3CN/DCM, DMF/DCM, and $CH_3CN/CHCl_3$) combining a favourable and unfavourable solvent in a 1:1 ratio were selected (**Figure S11a**). The corresponding PXRD patterns of MIL-53(Cr) in the above five solvent mixtures were also measured (**Figure S10b**). According to PXRD analysis, the most intense peaks of these five PXRD patterns are all positioned at 2θ = 9.4° and 9.7°, representing an aperture with R = 6.20/5.71 Å with a slight difference in the ratio of the two phases. This interesting phenomenon results not only from the host-guest interaction between the solvent molecules with the MOF framework but also from the solvent-solvent interactions. Featuring similar pore structures, the yields achieved in the above five solvent mixtures are obviously different, which is ascribed to the solvent properties.

To reduce the impact of inherent solvent properties (such as the polarity, kinetic diameter (**Figure S12**) or solvation effect) and for a deeper understanding of the factors influencing catalytic activity, two pairs of mixed solvent systems, $CH_3CN/CHCl_3$, and CH_3CN/DCM mixtures in different ratios were selected for further study. It is interesting to find that only a tiny amount of $CHCl_3$ added to CH_3CN, even in a ratio of just 1:19, will result in a dramatically lowered catalytic efficiency (yield of 9%) compared to the high yield of 86% obtained in sole CH_3CN (**Figure S11b**). The usage of $CHCl_3$ quenches the catalysis, regardless of the pore size of MOF (**Figure S13a**). For the case of CH_3CN/DCM (**Figure S13b**), MIL-53(Cr) in pure CH_3CN or DCM both exhibits fully open pore apertures (R = 7.61 and 7.50 Å, respectively), it shows a tendency that the pores slightly reduced in size when CH_3CN and DCM are mixed in any ratio, and 1:1 ratio presents the smallest value (R = 5.71 Å), which results probably from both host-solvent and solvent-solvent interactions. Combined with the catalytic results (**Figure S11c**) for analysis, it is reasonable to conclude that pore confinement and solvent effects interplay determines the catalytic activity, and the final photocatalytic performance could be thus finely tuned via solvent control.

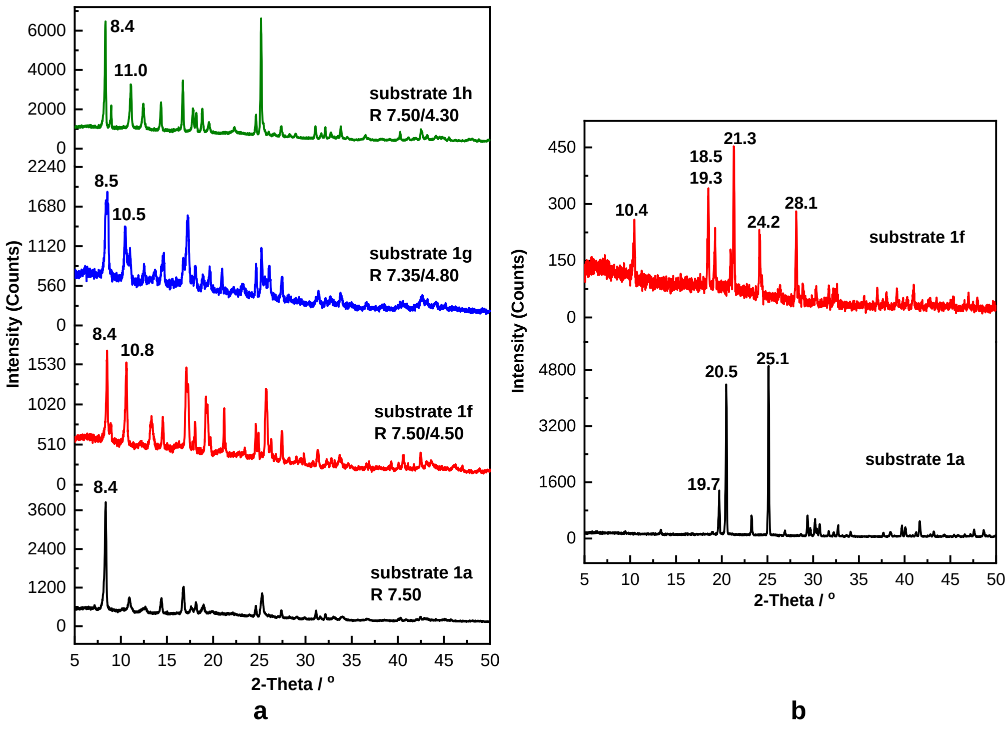

Figure S14. (**a**) The PXRD patterns of MIL-53(Cr)-lp in CH_3CN loaded with different substrates (**1a**, **1f**, **1g**, and **1h**); (**b**) The PXRD pattern of the solid 4'-bromoacetophenone (**1a**) and 4-iodoanisole (**1f**) substrates.

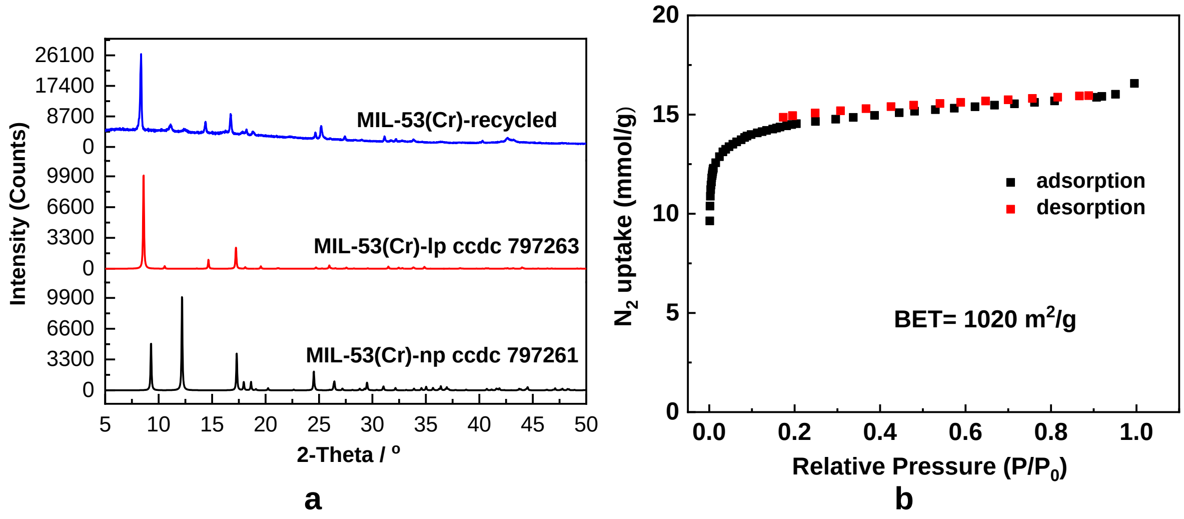

Figure S15. The PXRD pattern and N_2 adsorption/desorption isotherm (77 K) of used/recycled MIL-53(Cr) from photocatalytic reactions.

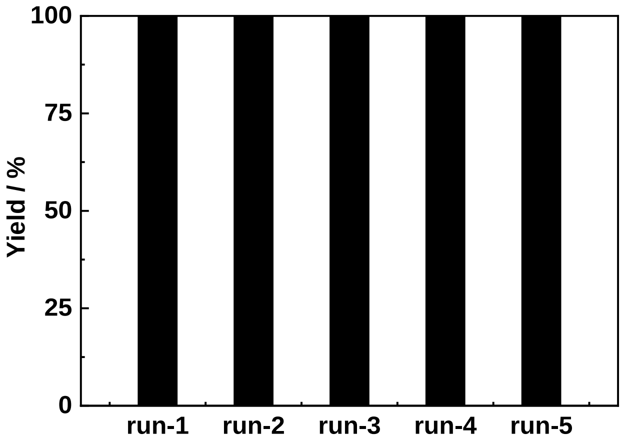

Figure S16. The recycling test and yield of product in each cycle.

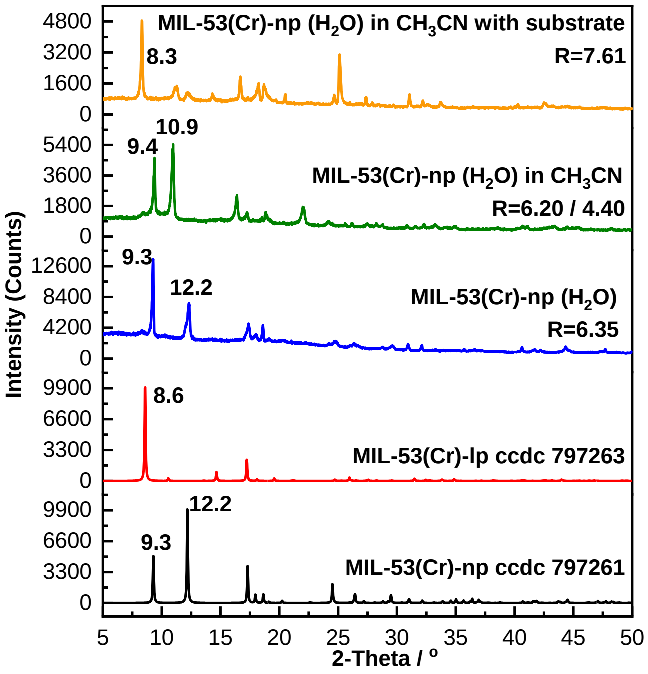

Figure S17. The PXRD patterns of MIL-53(Cr)-np (H_2O), MIL-53(Cr)-np in CH_3CN and MIL-53(Cr)-np in CH_3CN with substrate (4'-bromoacetophenone) added. The structure information for simulated large pore phase (CCDC 797263) is a = 6.812, b = 16.733, c = 13.038 and $\alpha = \beta = \gamma = 90°$, for simulated narrow pore phase (CCDC 797261)[9] is a = 6.782, b = 19.685, c = 7.849 and $\alpha = \gamma = 90°$, β = 104.90°.

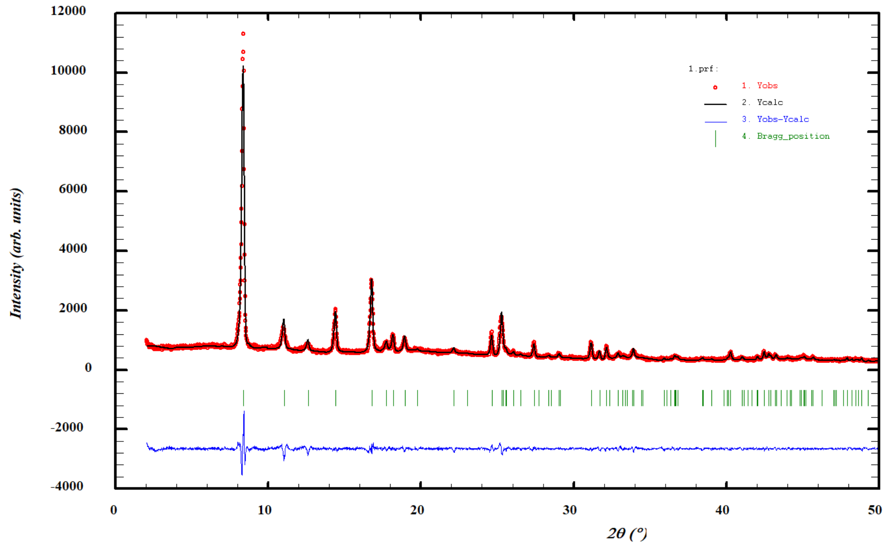

Figure S18. Le Bail fit of MIL-53(Cr), soaked in acetonitrile (*Imma*, a = 6.8329(2) Å, b = 16.0365(7) Å, c = 14.0293(6) Å, V = 1537.2(1) Å^3, R_{wp} = 0.0623, R_p = 0.0471).

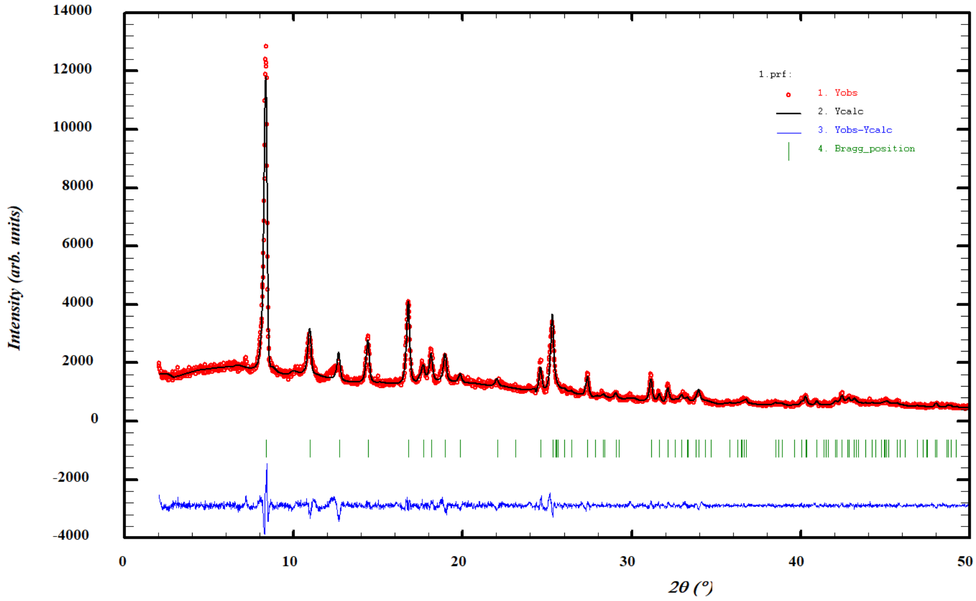

Figure S19. Le Bail fit of MIL-53(Cr), soaked in acetonitrile containing reaction adduct (*Imma*, a = 6.8376(3) Å, b = 16.1162(12) Å, c = 13.9417(10) Å, V = 1536.3(2) Å^3, R_{wp} = 0.0595, R_p = 0.0458).

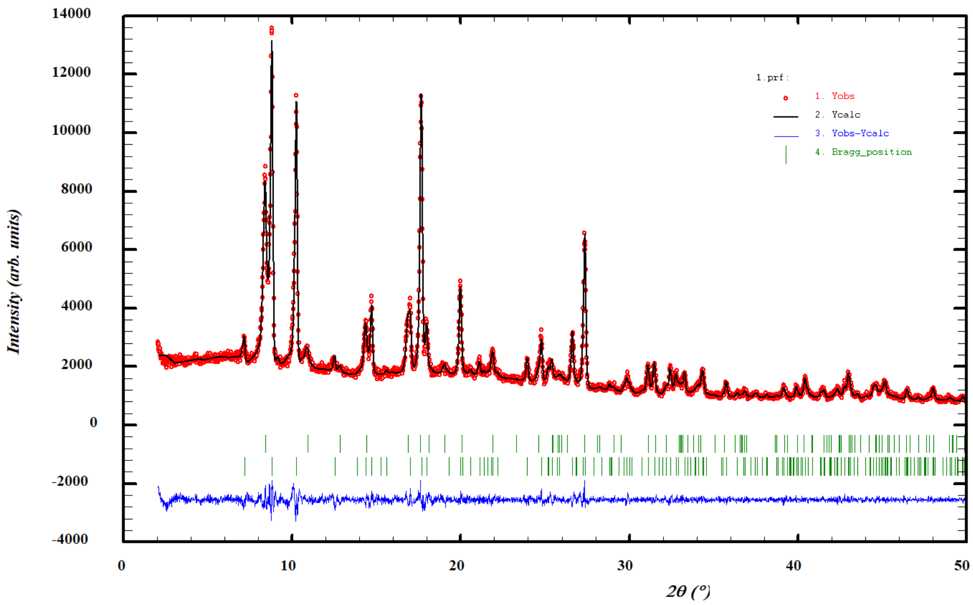

Figure S20. Le Bail fit of MIL-53(Cr), soaked in acetonitrile containing reaction product (**phase 1**: *Imma*, a = 6.8556(10) Å, b = 16.2241(23) Å, c = 13.7771(18) Å, V = 1532.3(3) Å^3; **phase 2**: *Pmmn*, a = 6.8844(1) Å, b = 17.2371(5) Å, c = 12.3370(3) Å, V = 1464.00(6) Å^3; R_{wp} = 0.0464, R_p = 0.0365).

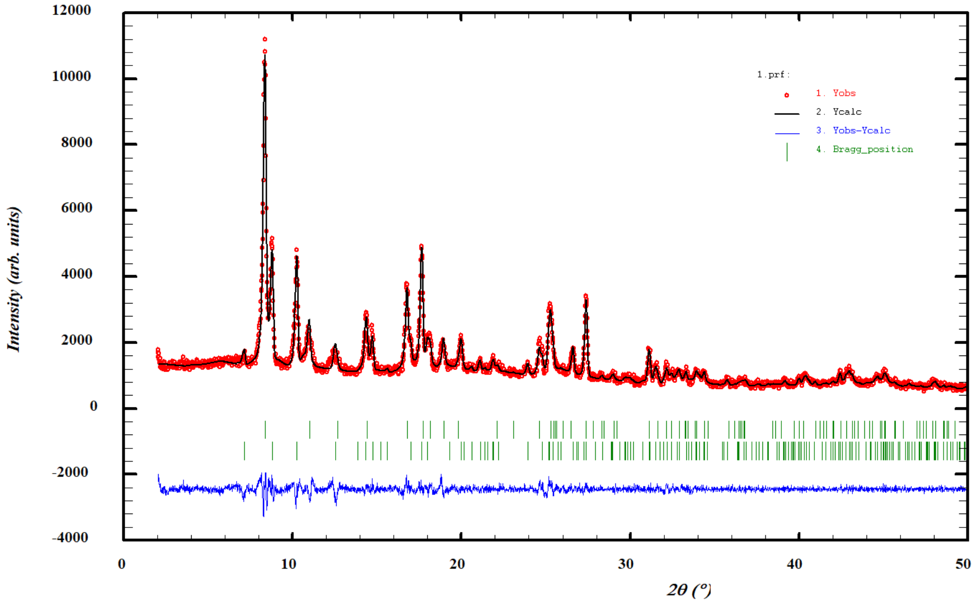

Figure S21. Le Bail fit of MIL-53(Cr), soaked in acetonitrile containing reaction adduct and product in ratio 1:1 (**phase 1**: *Imma*, a = 6.8413(6) Å, b = 16.0802(20) Å, c = 13.9846(16) Å, V = 1538.4(3) Å^3; **phase 2**: *Pmmn*, a = 6.8751(2) Å, b = 17.2302(11) Å, c = 12.3486(6) Å, V = 1462.8(1) Å^3; R_{wp} = 0.0656, R_p = 0.0507).

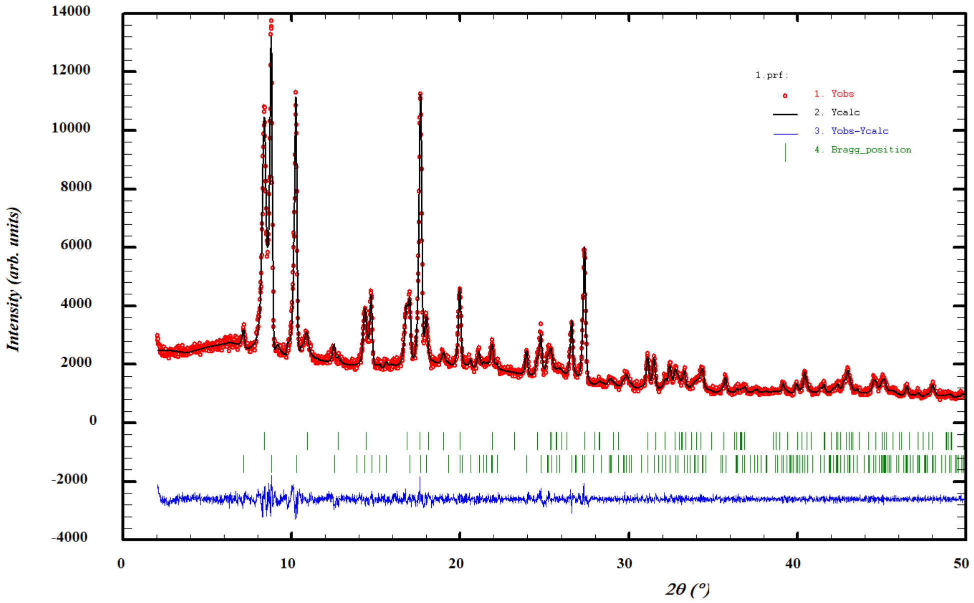

Figure S22. Le Bail fit of MIL-53(Cr), soaked in acetonitrile containing reaction adduct and product in ratio 1:4 (**phase 1**: *Imma*, a = 6.8470(9) Å, b = 16.2171(24) Å, c = 13.8550(22) Å, V = 1538.4(4) Å^3; **phase 2**: *Pmmn*, a = 6.8798(1) Å, b = 17.2284(5) Å, c = 12.3475(3) Å, V = 1463.53(7) Å^3; R_{wp} = 0.0462, R_p = 0.0364).

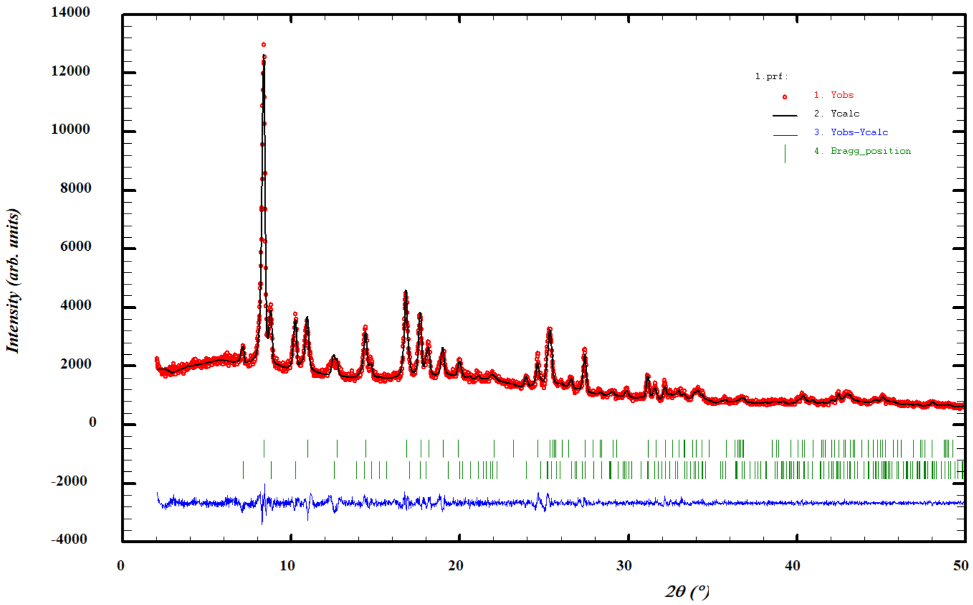

Figure S23. Le Bail fit of MIL-53(Cr), soaked in acetonitrile containing reaction adduct and product in ratio 4:1 (**phase 1**: *Imma*, a = 6.8363(6) Å, b = 16.1259(16) Å, c = 13.9148 (13) Å, V = 1534.0(2) Å^3; **phase 2**: *Pmmn*, a = 6.8675(3) Å, b = 17.2341(15) Å, c = 12.3477(8) Å, V = 1461.4(2) Å^3; R_{wp} = 0.0502, R_p = 0.0392).

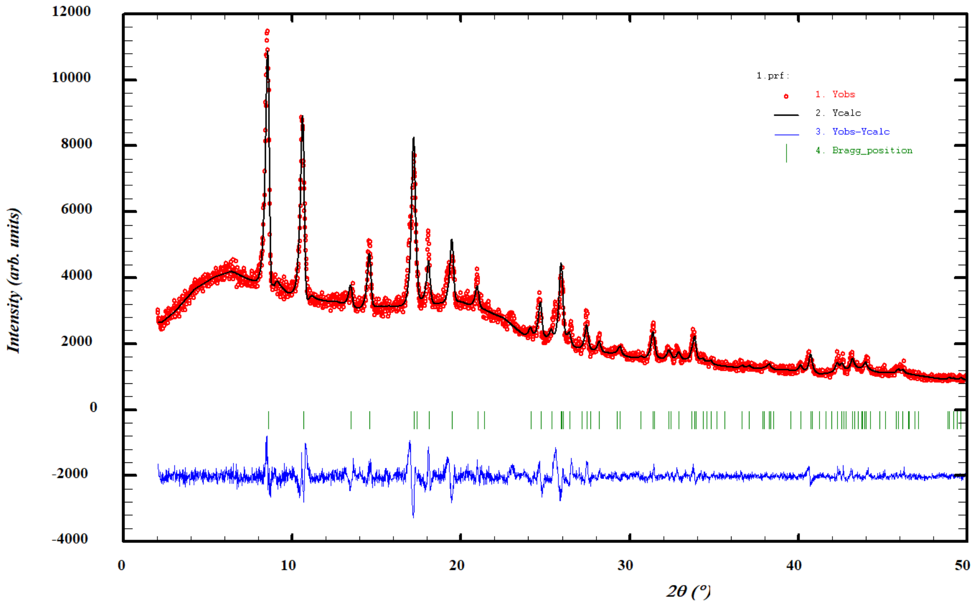

Figure S24. Le Bail fit of MIL-53(Cr), soaked in chloroform (*Imma*, a = 6.8360(6) Å, b = 16.6056(13) Å, c = 13.1232(12) Å, V = 1489.7(2) Å^3; R_{wp} = 0.0603, R_p = 0.0445).

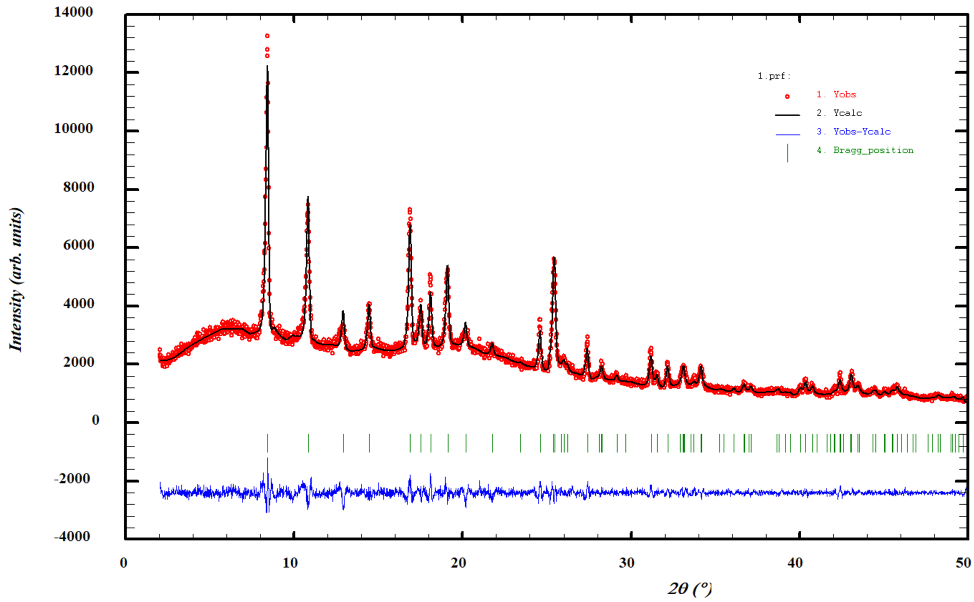

Figure S25. Le Bail fit of MIL-53(Cr), soaked in chloroform and reaction adduct (*Imma*, a = 6.8408(3) Å, b = 16.3173(8) Å, c = 13.6924(6) Å, V = 1528.4(1) Å^3; R_{wp} = 0.0491, R_p = 0.0385).

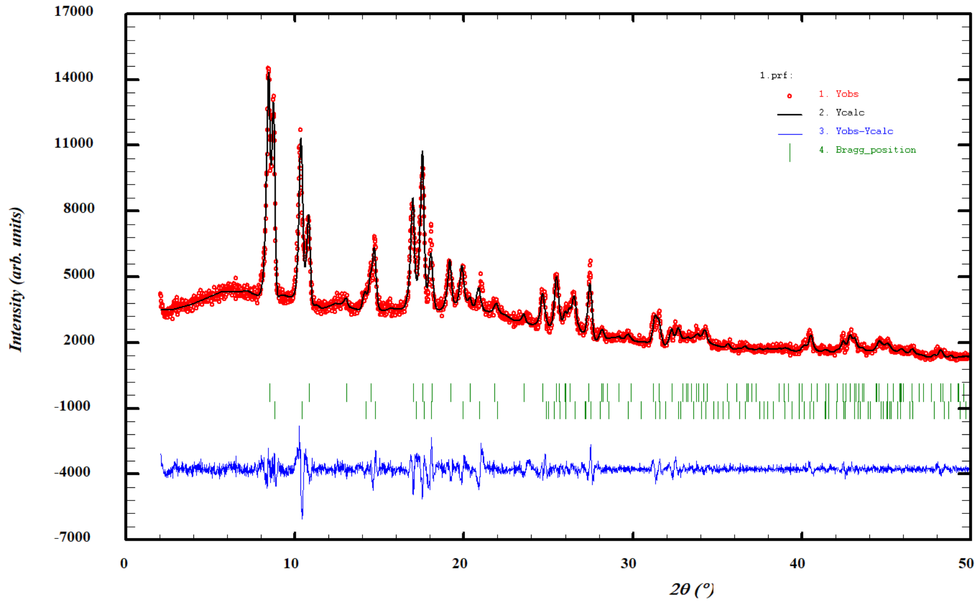

Figure S26. Le Bail fit of MIL-53(Cr), soaked in chloroform containing reaction adduct and product (**phase 1**: *Imma*, a = 6.8497(1) Å, b = 16.2987(10) Å, c = 13.5561(14) Å, V = 1513.4(3) Å^3; **phase 2**: *Imma*, a = 6.8469(4) Å, b = 16.9768(18) Å, c = 12.4862(16) Å, V = 1451.3(2) Å^3; R_{wp} = 0.0633, R_p = 0.0478).

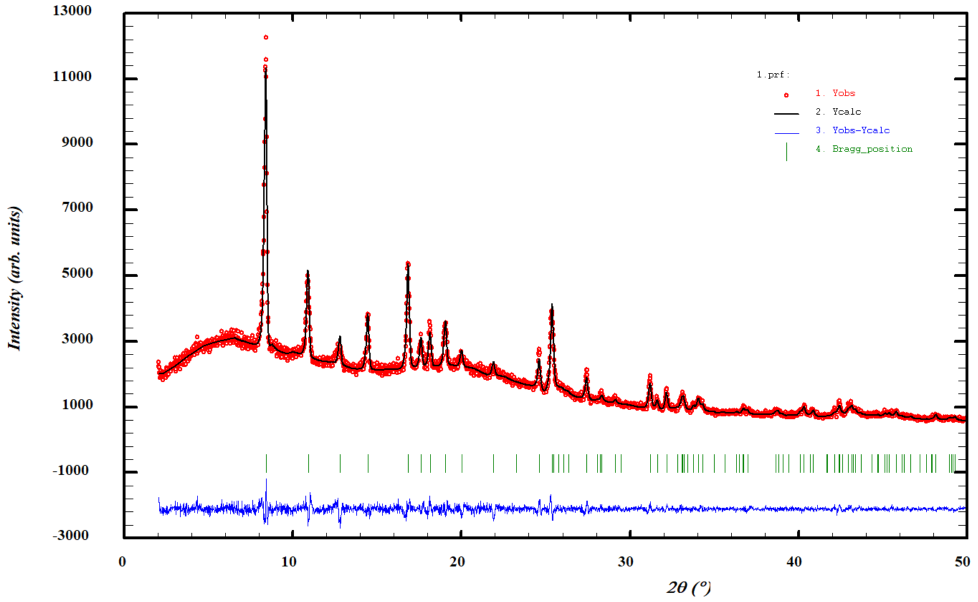

Figure S27. Le Bail fit of MIL-53(Cr), soaked in dichloromethane (*Imma*, a = 6.8300(3) Å, b = 16.2126(8) Å, c = 13.8221(7) Å, V = 1530.5(1) Å^3; R_{wp} = 0.0453, R_p = 0.0349).

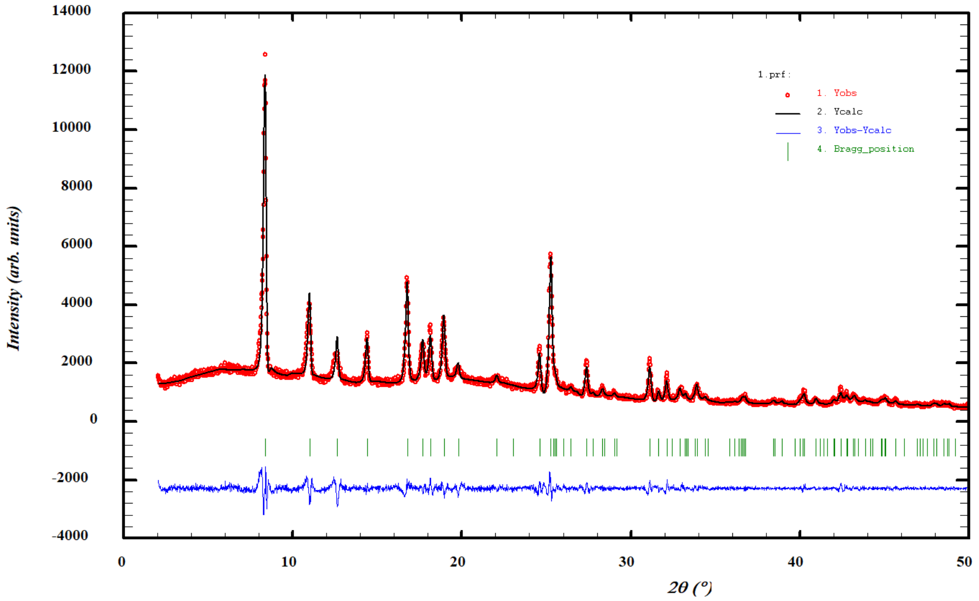

Figure S28. Le Bail fit of MIL-53(Cr), soaked in dichloromethane containing reaction adduct (*Imma*, *a* = 6.8392(2) Å, *b* = 16.0891(8) Å, *c* = 14.0023(6) Å, *V* = 1540.8(1) Å^3; R_{wp} = 0.0616, R_p = 0.0466).

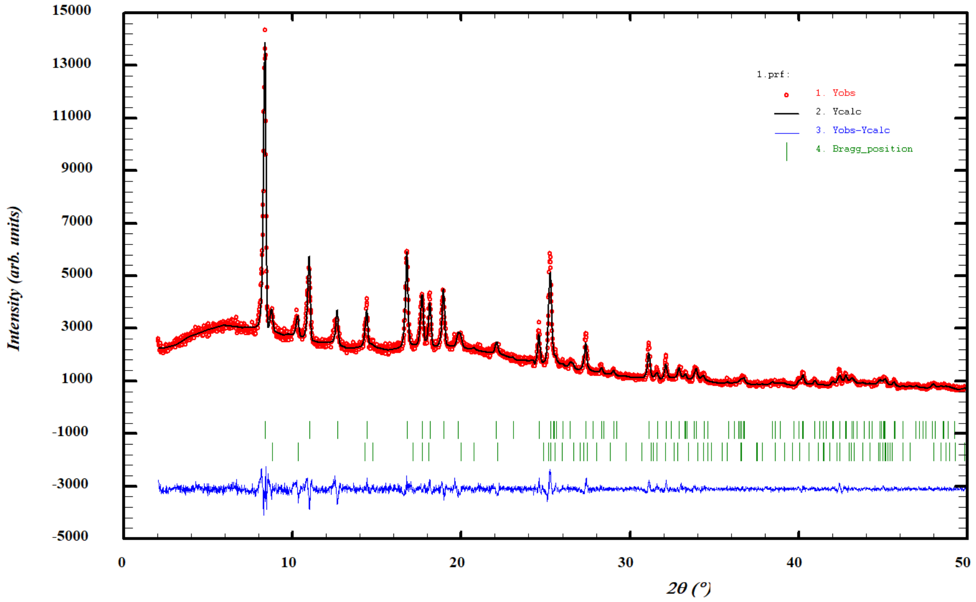

Figure S29. Le Bail fit of MIL-53(Cr), soaked in dichloromethane containing reaction adduct and product (**phase 1**: *Imma*, *a* = 6.8409(1) Å, *b* = 16.0899(8) Å, *c* = 13.9851(7) Å, *V* = 1539.3(1) Å^3; **phase 2**: *Imma*, *a* = 6.8582(8) Å, *b* = 17.0957(12) Å, *c* = 12.3856(15) Å, *V* = 1452.1(3) Å^3; R_{wp} = 0.0499, R_p = 0.0383).

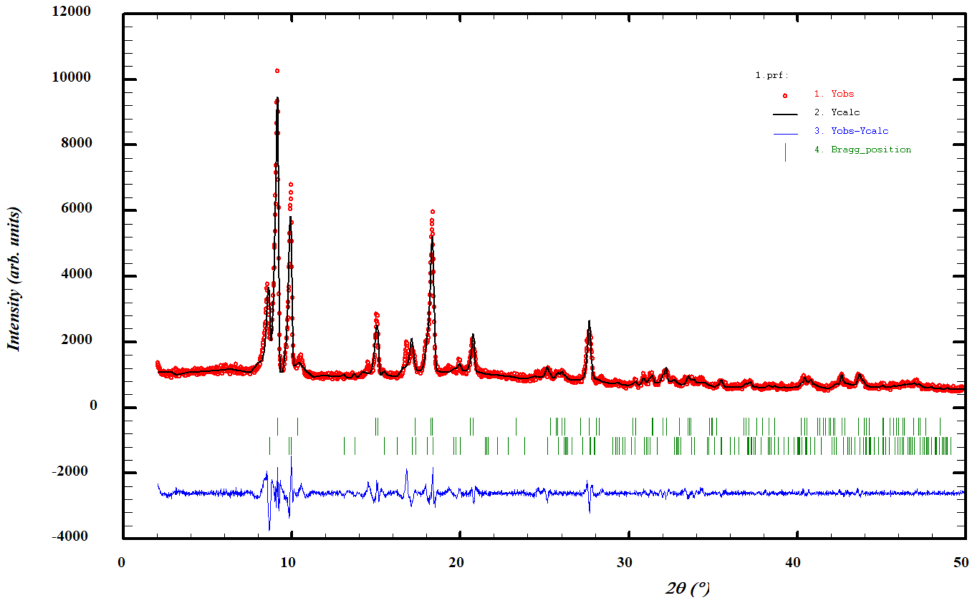

Figure S30. Le Bail fit of MIL-53(Cr), soaked in DMF (**phase 1**: *Imma*, a = 6.8420(6) Å, b = 17.0996(15) Å, c = 11.7425(7) Å, V = 1373.8(2) Å^3; **phase 2**: *Pmmn*, a = 6.8997(6) Å, b = 17.9934(25) Å, c = 10.2175(9) Å, V = 1268.4 (2) Å^3; R_{wp} = 0.0841, R_p = 0.0611).

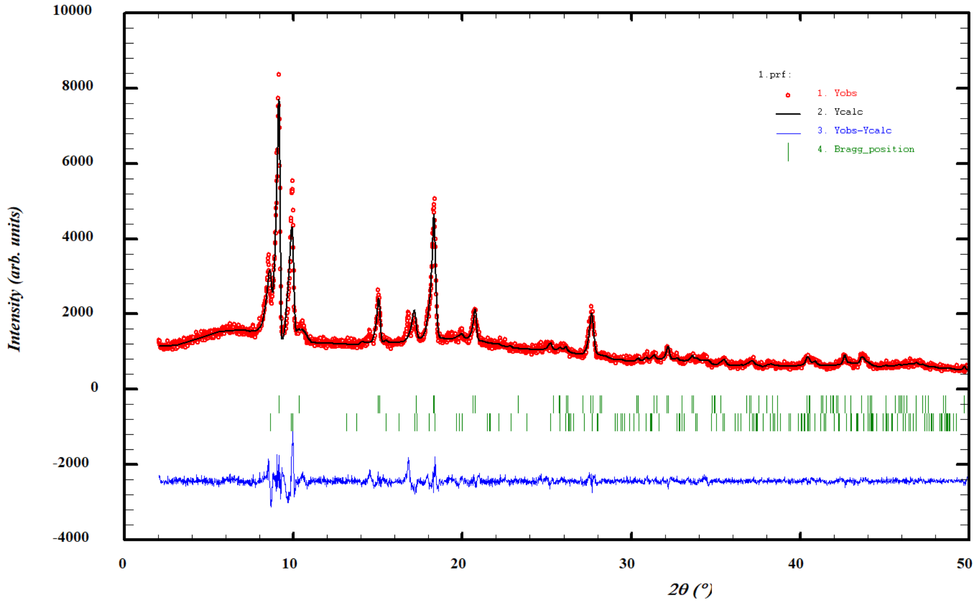

Figure S31. Le Bail fit of MIL-53(Cr), soaked in DMF containing reaction adduct (**phase 1**: *Imma*, a = 6.8144(11) Å, b = 17.1061(18) Å, c = 11.7486(9) Å, V = 1369.5(3) Å^3; **phase 2**: *Pmmn*, a = 6.9070(10) Å, b = 17.9140(38) Å, c = 10.2352(15) Å, V = 1266.4 (4) Å^3; R_{wp} = 0.0686, R_p = 0.0512).

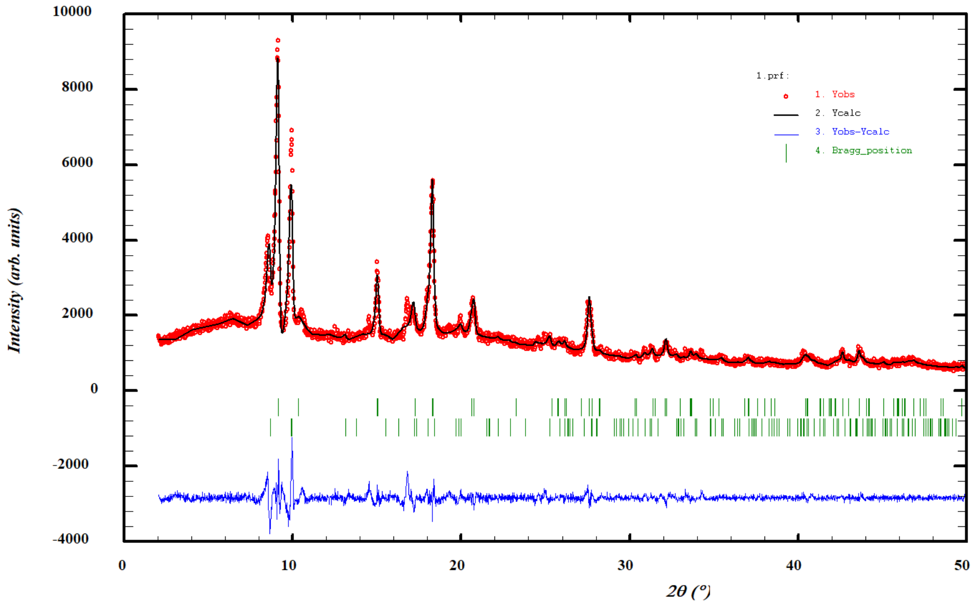

Figure S32. Le Bail fit of MIL-53(Cr), soaked in *DMF* containing reaction adduct and product (**phase 1**: *Imma*, a = 6.8094(8) Å, b = 17.1002(16) Å, c = 11.7536(10) Å, V = 1368.6(3) Å^3; **phase 2**: *Pmmn*, a = 6.8930(3) Å, b = 17.8816(50) Å, c = 10.2229(26) Å, V = 1260.0(7) Å^3; R_{wp} = 0.0678, R_p = 0.0508).

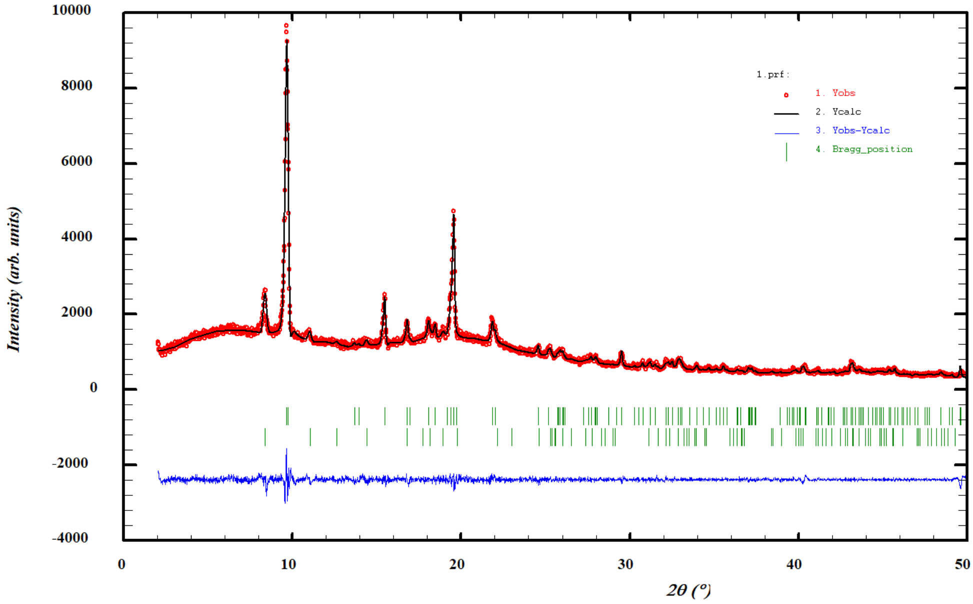

Figure S33. Le Bail fit of MIL-53(Cr), soaked in DMSO (**phase 1**: $C2$, a = 19.3770(9) Å, b = 10.4373(12) Å, c = 6.8486(8) Å, β = 109.04(5)°, V = 1309.3(6) Å^3; **phase 2**: *Imma*, a = 6.8433(8) Å, b = 16.0352(12) Å, c = 14.0261(16) Å, V = 1539.1(4) Å^3; R_{wp} = 0.0482, R_p = 0.0355).

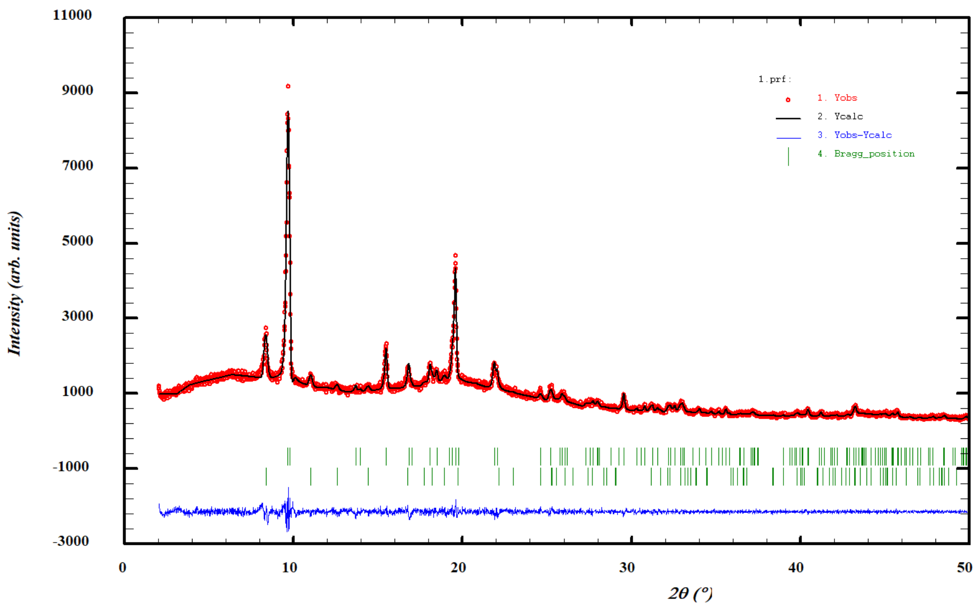

Figure S34. Le Bail fit of MIL-53(Cr), soaked in DMSO containing adduct (**phase 1**: *C*2, a = 19.3518(9) Å, b = 10.4236(3) Å, c = 6.8400(4) Å, β = 109.03(6)°, V = 1304.3(1) Å^3; **phase 2**: *Imma*, a = 6.8297(8) Å, b = 16.0422(22) Å, c = 14.0695(16) Å, V = 1541.5(3) Å^3; R_{wp} = 0.0502, R_p = 0.0394).

Supplementary Tables

Supplementary Table 1. Parameters of simulated crystal structures including unit cell parameters, unit cell volumes and 2θ values of characteristic peaks in PXRD patterns.

Entry	b / Å	c / Å	Pore size / Å	Volume cm^3/g	2θ*(011) reflection*
1	16.0	14.5	7.80	0.56	8.2
2	16.6	13.9	7.61	0.55	8.3
3	16.9	13.4	7.50	0.52	8.4
4	16.5	13.0	7.19	0.50	8.6
5	16.6	12.7	7.00	0.46	8.8
6	17.0	12.0	6.80	0.45	9.0
7	17.3	11.6	6.40	0.41	9.2
8	17.5	11.0	6.20	0.39	9.4
9	17.6	10.8	6.10	0.38	9.6
10	18.0	10.5	5.71	0.37	9.7
11	18.1	10.3	5.40	0.34	9.9
12	18.3	10.0	5.21	0.33	10.1
13	18.4	9.90	5.10	0.32	10.2
14	18.7	9.50	4.80	0.30	10.5
15	18.9	9.30	4.70	0.28	10.6
16	19.0	9.00	4.40	0.26	10.9
17	19.5	8.50	4.00	0.21	11.4
18	20.0	8.00	3.60	0.19	11.9

*The a axis is defined by Cr-O-Cr chains and remains constant (a = 6.61 Å) upon breathing of the structure.

Supplementary Table 2. Selected MOFs used for photocatalysts.

Entry	Photocatalyst	metal	ligand	Bandgap eV	Ref.
1	MIL-125(Ti)<br>NH_2-MIL-125(Ti)	Ti(IV)	1,4-benzenedicarboxylate (bdc)<br>2-amino-bdc	3.65*<br>2.60	[15]<br>[16]
2	MUV11	Ti(IV)	benzene-1,4-dihydroxamate	2.50	[17]
3	NTU-9	Ti(IV)	2,5-dihydroxyterephthalate	1.74	[18]
4	ZSTU-1	Ti(IV)	4,4′,4″- nitrilotribenzoate	2.30	[19]
5	ZSTU-2	Ti(IV)	1,3,5-tris(4-carboxyphenyl) benzene	3.10	[19]
6	UiO-66(Zr)<br>NH_2-UiO-66(Zr)	Zr(IV)	bdc<br>2-amino-bdc	3.65<br>2.75	[20]<br>[21]
7	NU-1000	Zr(IV)	1,3,6,8-tetrakis(p-benzoic acid)pyrene	2.72	[22]
8	MIL-53(Fe)	Fe(III)	bdc	2.62	[23]
9	MIL-101(Fe)	Fe(III)	bdc	2.77	[24]
10	MIL-88A(Fe)	Fe(III)	fumarate	2.63	[25]
11	NH_2-MIL-88B(Fe)	Fe(III)	2-amino-bdc	1.97	[26]
12	MOF-5	Zn(II)	bdc	3.4	[27]
13	ZIF-8	Zn(II)	imidazolate	3.3	[28]
14	Zn_2TTFB	Zn(II)	tetrathiafulvalene(TTF)-tetrabenzoate	--	[29]
15	MIL-68(In)	In(III)	bdc	3.94	[30]
16	MFM-300(Ga)	Ga(III)	biphenyl-3,3′,5,5′- tetracarboxylic acid(H_4L)	3.30	[31]
17	Al-PMOF	Al(III)	porphyrin	1.70*	[32]

18	BIT-66	V(III)	1,3,5-tris(4-carboxyphenyl) benzene	2.17	[33]
19	ZIF-67	Co(II)	2-methylimidazole	1.98	[34]
20	MUV-10(Ti/Ca)	Ca(II)	benzene-1,3,5-tricarboxylic acid	3.10	[35]
21	MUV-10(Ti/Mn)	Mn(II)	benzene-1,3,5-tricarboxylic acid	2.60	[35]
22	Cd-TBAPy	Cd(II)	1,3,6,8-tetrakis(p-benzoic acid)pyrene	2.15	[36]
23	Mg-MOF-74	Mg(II)	2,5-dihydroxyterephthalic acid	2.63	[37]
24	Cu-MOF-74	Cu(II)	2,5-dihydroxyterephthalic acid	1.67	[37]
25	Ni-MOF	Ni(II)	bdc	--	[38]
26	MIL-101(Cr)	Cr(III)	bdc	2.28	[39]
27	MFM-300(Cr)		biphenyl-3,3′,5,5′- tetracarboxylic acid	1.76	[40]
28	Cr-PCN-600		tetrakis(4-carboxyphenyl)porphyrin	--	[41]
29	Cr-MOF		polyethylene terephthalate	3.49	[42]

*band gap value calculated from the UV-DRS spectrum

Supplementary Table 3. Summary of catalysis results for control experiments.

Entry	Catalyst	sacrificial agent	Solvent	Light / nm	Product yield / %
**1**	**MIL-53(Cr)-lp**	**TEA**	**CH_3CN**	**350-780**	**86%**
2	MIL-53(Cr)-lp	TEA	CH_3CN	dark	n.a.
3	$Cr(NO_3)_3·9H_2O$-H_2BDC	TEA	CH_3CN/H_2O 1:1	350-780	2%
4	MIL-53(Cr)-lp	Na_2SO_3	CH_3CN/H_2O 1:1	350-780	15%
5	MIL-53(Cr)-lp	--	CH_3CN	350-780	17%
6	MIL-53(Cr)-np (H_2O)	TEA	CH_3CN	350-780	≥99%
7	MIL-53(Cr)-lp	TEA	CH_3CN/H_2O 1:1	350-780	≥99%

Reaction conditions: model substrate (4’-bromoacetophenone, 0.50 mmol), MIL-53(Cr) (9 mol%, 0.01 g), TEA (1.0 mmol), solvent (5 mL), 25 ºC, 350-780 nm, reaction time 2 h. For entry 3, a powdered mixture of $Cr(NO_3)_3·9H_2O$ (0.1 mmol, 0.040g) and H_2BDC (0.1 mmol, 0.033g) were used (H_2BDC = terephthalic acid). For entry 4, the sacrificial agent TEA was replaced by Na_2SO_3 (0.5 M), H_2O was added to dissolve it.

Supplementary spectroscopic data and NMR spectrum

4-Bromoacetophenone (**1a**) [43], [44] and acetophenone (**2a**, Figure 4) [43], [44]

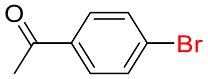 and 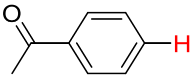

1a **2a**

According to the general procedure, the reaction of 4-bromoacetophenone (99.5 mg, 0.500 mmol) and Et_3N (101 mg, 1.00 mmol) in CH_3CN (5 mL) was catalysed by MIL-53(Cr) (9 mol %, 0.01 g). **2a** was obtained in 86% after 2 h. After chromatography using $CHCl_3$, **2a** was isolated as a colourless liquid with 49.9 mg in 83% yield.

^{1}H NMR of **1a** (300 MHz, DMSO-*d6*) δ 7.89 (d, 2H), 7.73 (d, 2H), 2.51 (s, 3H);
^{1}H NMR of **2a** (300 MHz, DMSO-*d6*) δ 7.97 (d, 2H), 7.64 (t, 1H), 7.53 (t, 2H), 2.58 (s, 3H);

4-Bromo-benzaldehyde (**1b**) [45] and benzaldehyde (**2b**, Figure 4) [43], [45]

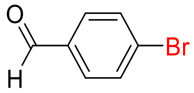 and 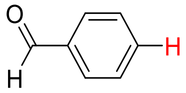

1b **2b**

According to the general procedure, the reaction of 4-bromo-benzaldehyde (92.5 mg, 0.500 mmol) and Et_3N (101 mg, 1.00 mmol) in CH_3CN (5 mL) was catalysed by MIL-53(Cr) (9 mol %, 0.01 g). **2b** was obtained in 66% after 2 h. After chromatography using $CHCl_3$, **2b** was isolated as a light-yellow liquid with 34.5 mg in 65% yield.
^{1}H NMR of **1b** (300 MHz, DMSO-*d6*) δ 10.00 (s, 1H), 7.83 (m, 4H);
^{1}H NMR of **2b** (300 MHz, DMSO-*d6*) δ 10.02 (s, 1H), 7.92 (d, 2H), 7.72 (t, 1H), 7.61 (t, 2H);

1-(6-Bromopyridin-3-yl) ethanone (**1c**) [44] and methyl-3-pyridylketone (**2c**, Figure 4) [46]

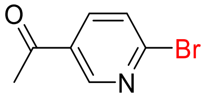 and 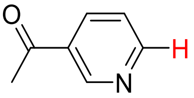

1c **2c**

According to the general procedure, the reaction of 1-(6-bromopyridin-3-yl) ethanone (100 mg, 0.500 mmol) and Et_3N (101 mg, 1.00 mmol) in CH_3CN (5 mL) was catalysed by MIL-53(Cr) (9 mol %, 0.01 g). **2c** was obtained in 68% after 2 h. After chromatography using $CHCl_3$, **2c** was isolated as a yellow liquid with 39.4 mg in 65% yield.
^{1}H NMR of **1c** (300 MHz, DMSO-*d6*) δ 8.92 (d, 1H), 8.19 (m, 1H), 7.80 (d, 1H), 2.61 (s, 3H);
^{1}H NMR of **2c** (300 MHz, DMSO-*d6*) δ 9.14 (d, 1H), 8.79 (m, 1H), 8.28 (m, 1H), 7.55 (m, 1H), 2.63 (s, 3H);
^{1}H NMR of **2c** (300 MHz, $CDCl_3$) δ 9.17 (s, 1H), 8.79 (s, 1H), 8.24 (m, 1H), 7.43 (m, 1H), 2.64 (s, 3H);
^{13}C NMR of **2c** (100 MHz, $CDCl_3$) δ 196.8, 153.6, 150.0, 135.7, 132.5, 123.9, 26.9;

2-Choro-5-iodopyridine (**1d**) [44] and 2-chloropyridine (**2d**, Figure 4) [47]

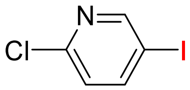 and 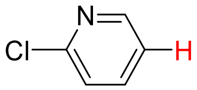

1d **2d**

According to the general procedure, the reaction of 2-choro-5-iodopyridine (120 mg, 0.500 mmol) and Et_3N (101 mg, 1.00 mmol) in CH_3CN (5 mL) was catalysed by MIL-53(Cr) (9 mol %, 0.01 g). **2d** was obtained in 32% after 2 h, 52% after 12 h and 68% after 24 h.
^{1}H NMR of **1d** (300 MHz, DMSO-*d6*) δ 8.66 (d, 1H), 8.19 (m, 1H), 7.37 (m, 1H);
^{1}H NMR of **2d** (300 MHz, DMSO-*d6*) δ 8.41 (m, 1H), 7.85 (m, 1H), 7.49 (m, 1H), 7.39 (m, 1H);

4-Iodobenzonitrile (**1e**) [44], [48] and benzonitrile (**2e**, Figure 4) [49]

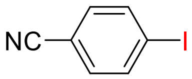

and
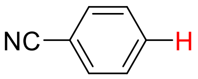

1e **2e**

According to the general procedure, the reaction of 4-iodobenzonitrile (115 mg, 0.500 mmol) and Et_3N (101 mg, 1.00 mmol) in CH_3CN (5 mL) was catalysed by MIL-53(Cr) (9 mol %, 0.01 g). **2e** was obtained in 41% after 2 h. After chromatography using $CHCl_3$, **2e** was isolated as a colourless liquid with 20.6 mg in 40% yield.
^{1}H NMR of **1e** (300 MHz, DMSO-*d6*) δ 7.98 (d, 2H), 7.58 (m, 2H);
^{1}H NMR of **2e** (300 MHz, DMSO-*d6*) δ 7.82 (d, 2H), 7.72 (t, 1H), 7.58 (m, 2H);

4-Iodoanisole (**1f**) [44] and anisole (**2f**, Figure 4) [50]

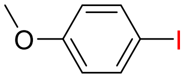

and
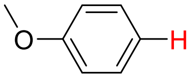

1f **2f**

According to the general procedure, the reaction of 4-iodoanisole (117 mg, 0.500 mmol) and Et_3N (101 mg, 1.00 mmol) in CH_3CN (5 mL) was catalysed by MIL-53(Cr) (9 mol %, 0.01 g). **2f** was obtained in 25% and 40% after 2 h and 12 h, respectively.
^{1}H NMR of **1f** (300 MHz, DMSO-*d6*) δ 7.61 (d, 2H), 6.79 (d, 2H), 3.75 (s, 3H);
^{1}H NMR of **2f** (300 MHz, DMSO-*d6*) δ 7.31 (t, 2H), 6.95 (m, 3H), 3.77 (s, 3H);

Ethyl 4-iodobenzoate (**1g**) [51] and ethyl benzoate (**2g**, Figure 4) [52]

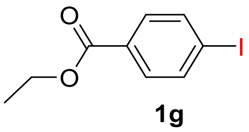

and
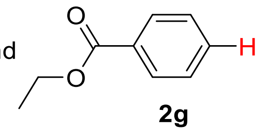

1g **2g**

According to the general procedure, the reaction of ethyl 4-iodobenzoate (138 mg, 0.500 mmol) and Et_3N (101 mg, 1.00 mmol) in CH_3CN (5 mL) was catalysed by MIL-53(Cr) (9 mol %, 0.01 g). **2g** was obtained in 12% and 27% after 2 h and 12 h, respectively.
^{1}H NMR of **1g** (300 MHz, DMSO-*d6*) δ 7.91 (d, 2H), 7.72 (d, 2H), 4.3 (q, 2H), 1.3 (t, 3H);
^{1}H NMR of **2g** (300 MHz, DMSO-*d6*) δ 7.98 (d, 2H), 7.65 (t, 1H), 7.52 (m, 2H), 4.3 (q, 2H), 1.3 (t, 3H);

1-Iodonaphthalene (**1h**) [48] and naphthalene (**2h**, Figure 4) [53]

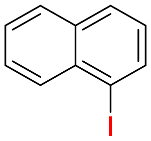

and
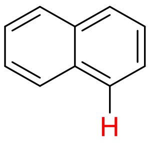

1h **2h**

According to the general procedure, the reaction of 1-Iodonaphthalene (127 mg, 0.500 mmol) and Et_3N (101 mg, 1.00 mmol) in CH_3CN (5 mL) was catalysed by MIL-53(Cr) (9 mol %, 0.01 g). **2h** was

obtained in 20% after 2 h. After chromatography, **2h** was isolated as a white solid with 11.5 mg in 18% yield.

^{1}H NMR of **1h** (300 MHz, DMSO-*d6*) δ 8.00 (m, 2H), 7.92 (m, 2H), 7.63 (m, 2H), 7.51 (m, 1H);

^{1}H NMR of **2h** (300 MHz, DMSO-*d6*) δ 8.15 (m, 4H), 7.51 (m, 4H);

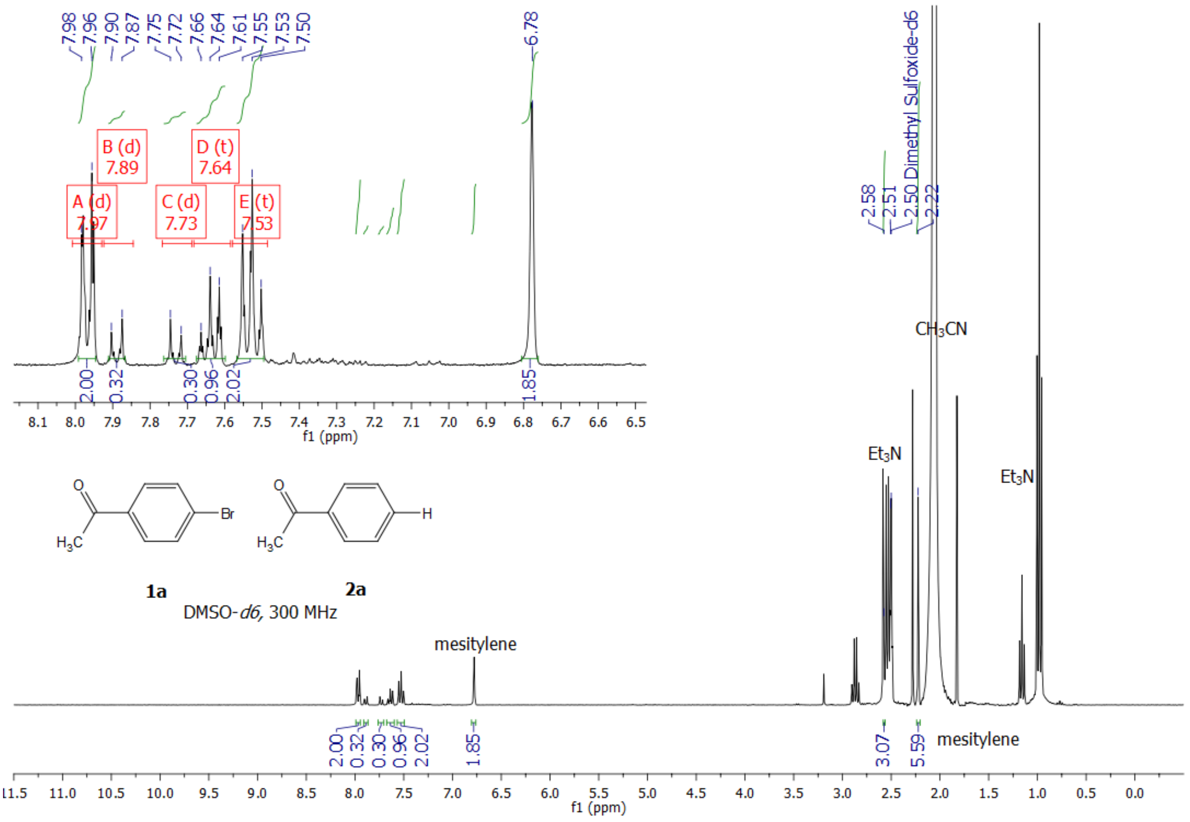

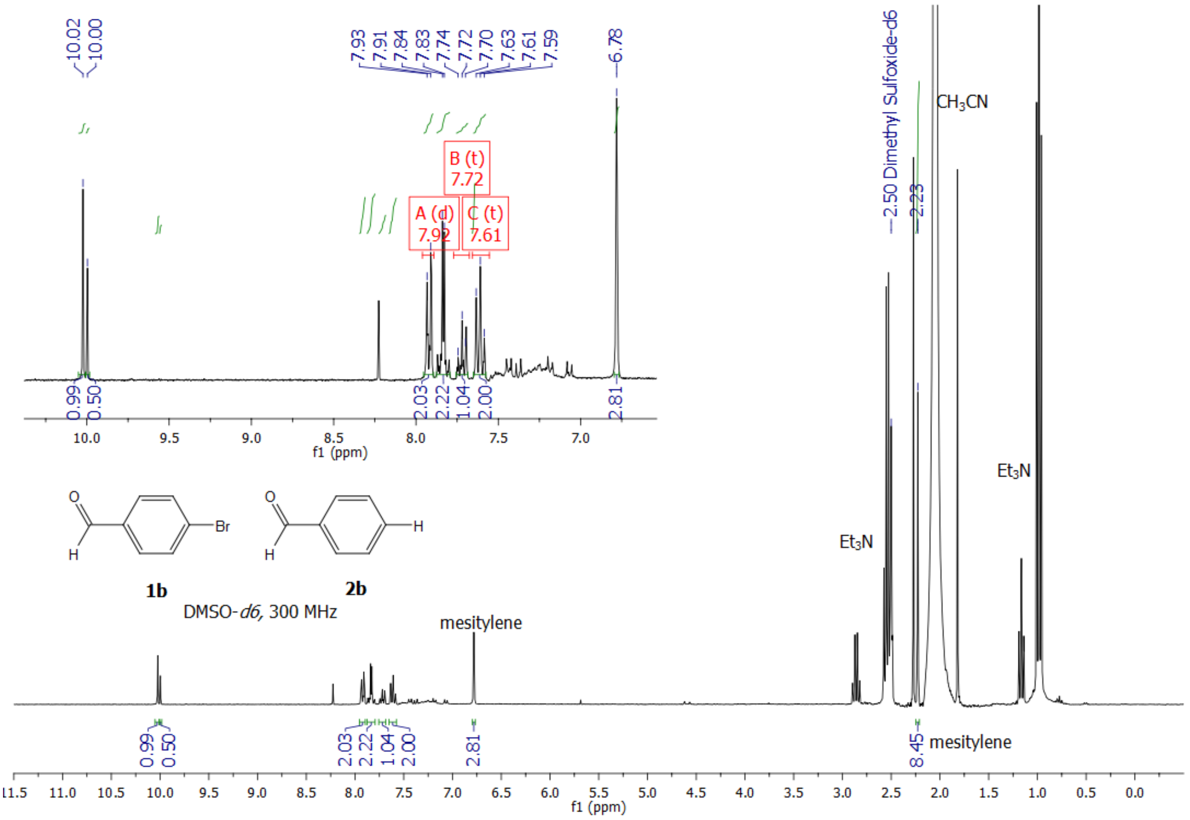
1b
2b
DMSO-d6, 300 MHz
mesitylene
Et3N
CH3CN
2.50 Dimethyl Sulfoxide-d6
A (d) 7.92
B (t) 7.72
C (t) 7.61
f1 (ppm)

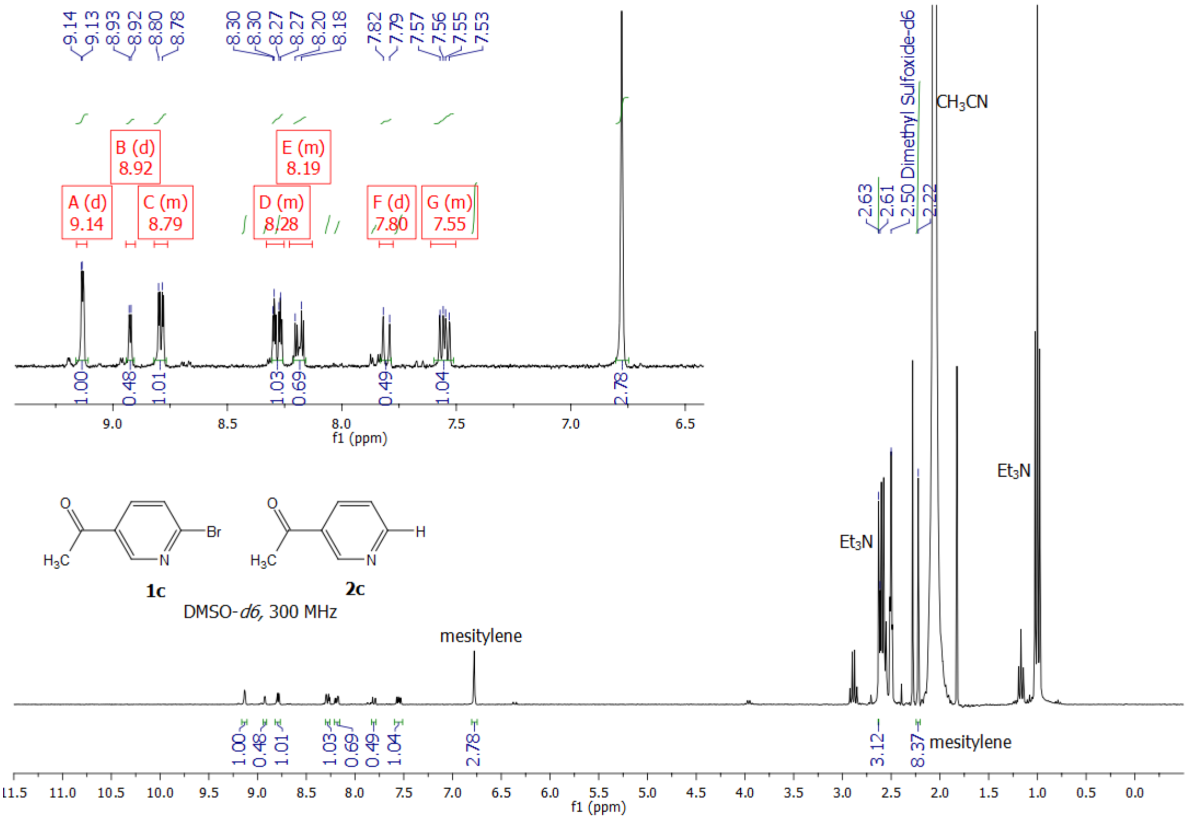
1c
2c
DMSO-d6, 300 MHz
mesitylene
Et3N
CH3CN
2.50 Dimethyl Sulfoxide-d6
A (d) 9.14
B (d) 8.92
C (m) 8.79
D (m) 8.28
E (m) 8.19
F (d) 7.80
G (m) 7.55
f1 (ppm)

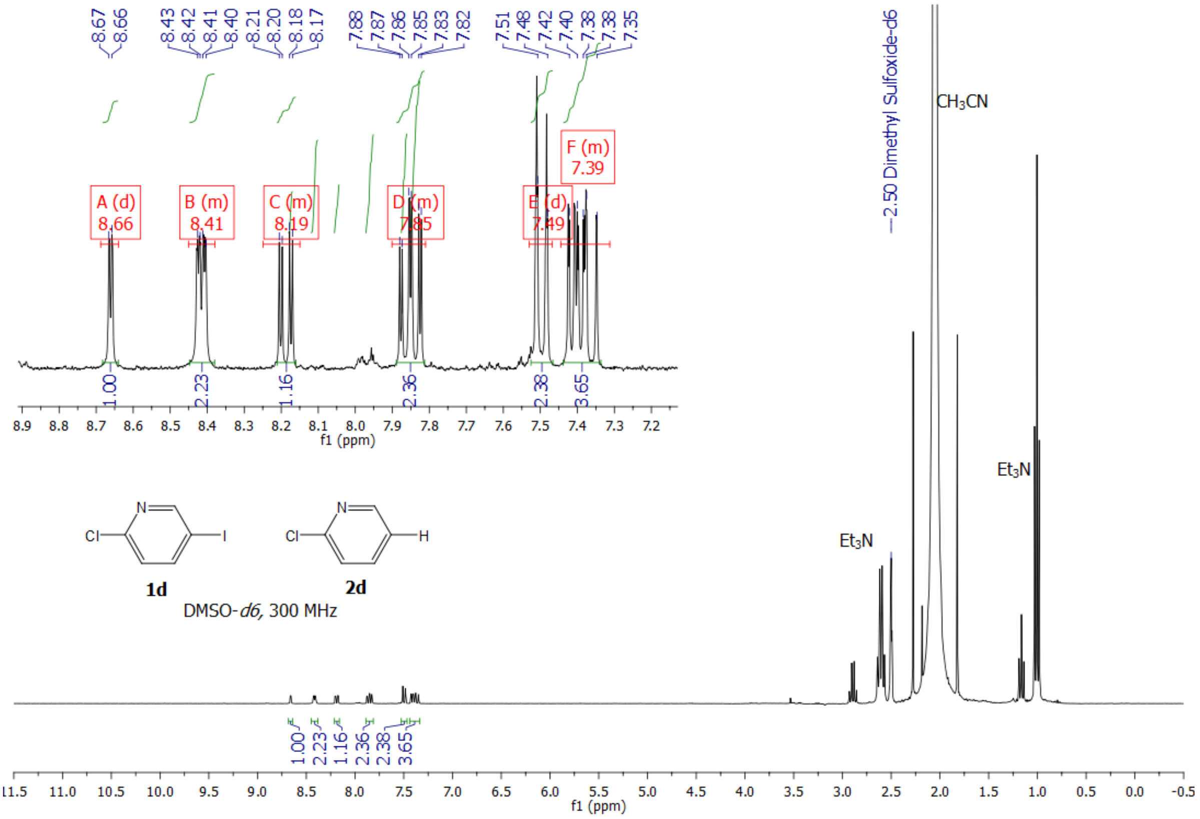

DMSO-*d6*, 300 MHz

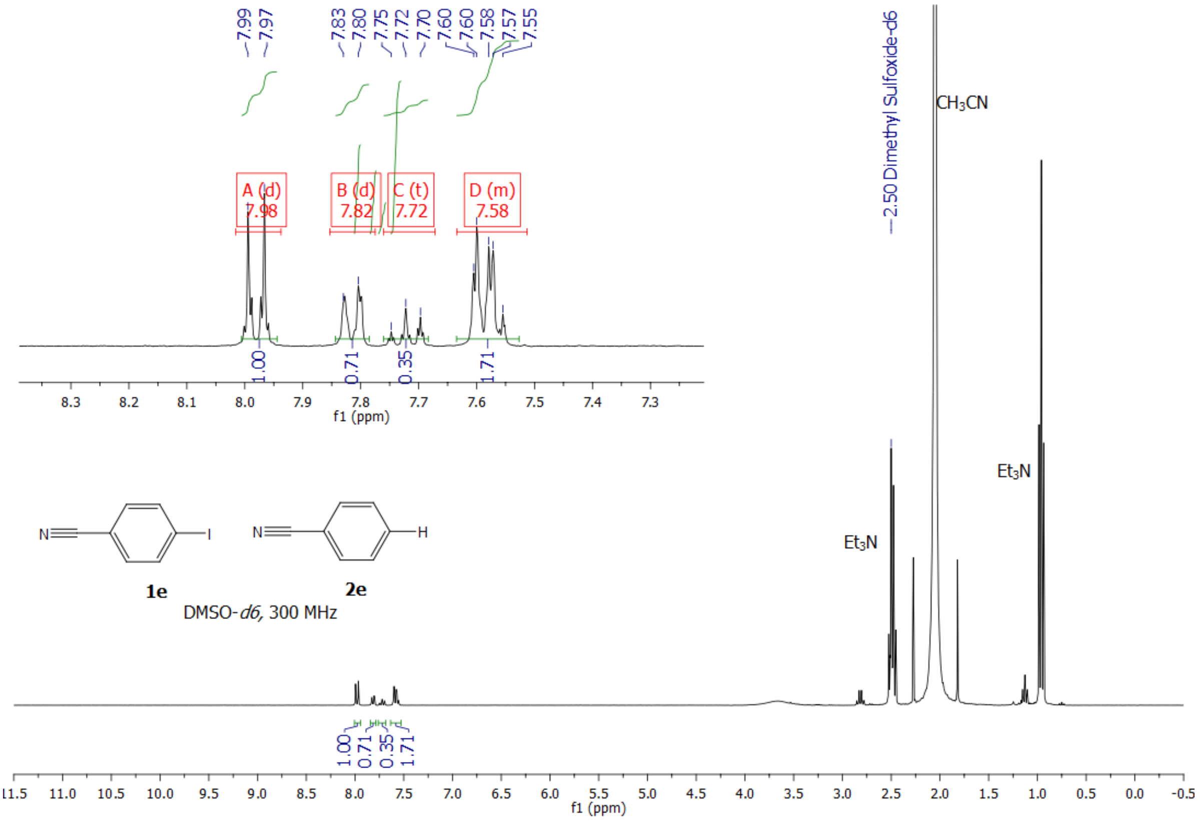

DMSO-*d6*, 300 MHz

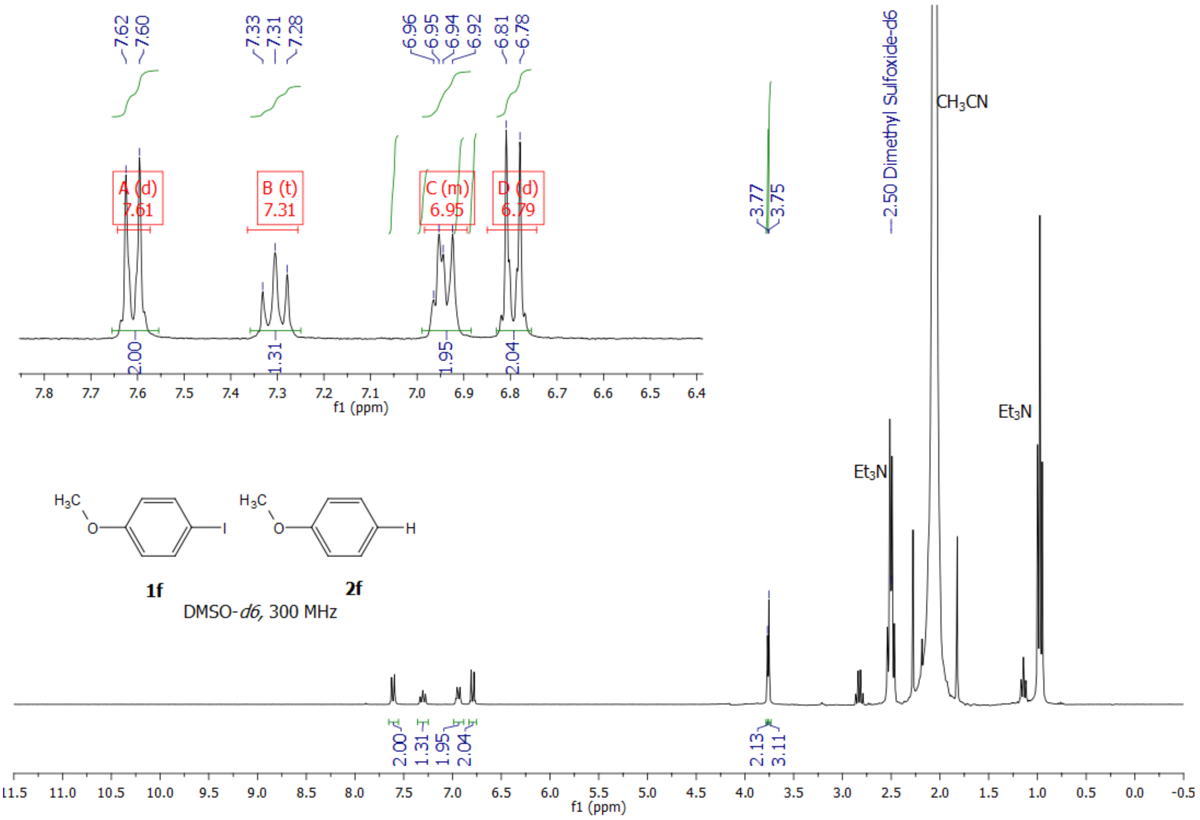

A (d) 7.61
B (t) 7.31
C (m) 6.95
D (d) 6.79
2.50 Dimethyl Sulfoxide-d6
CH3CN
Et3N
Et3N
1f
2f
DMSO-d6, 300 MHz
f1 (ppm)

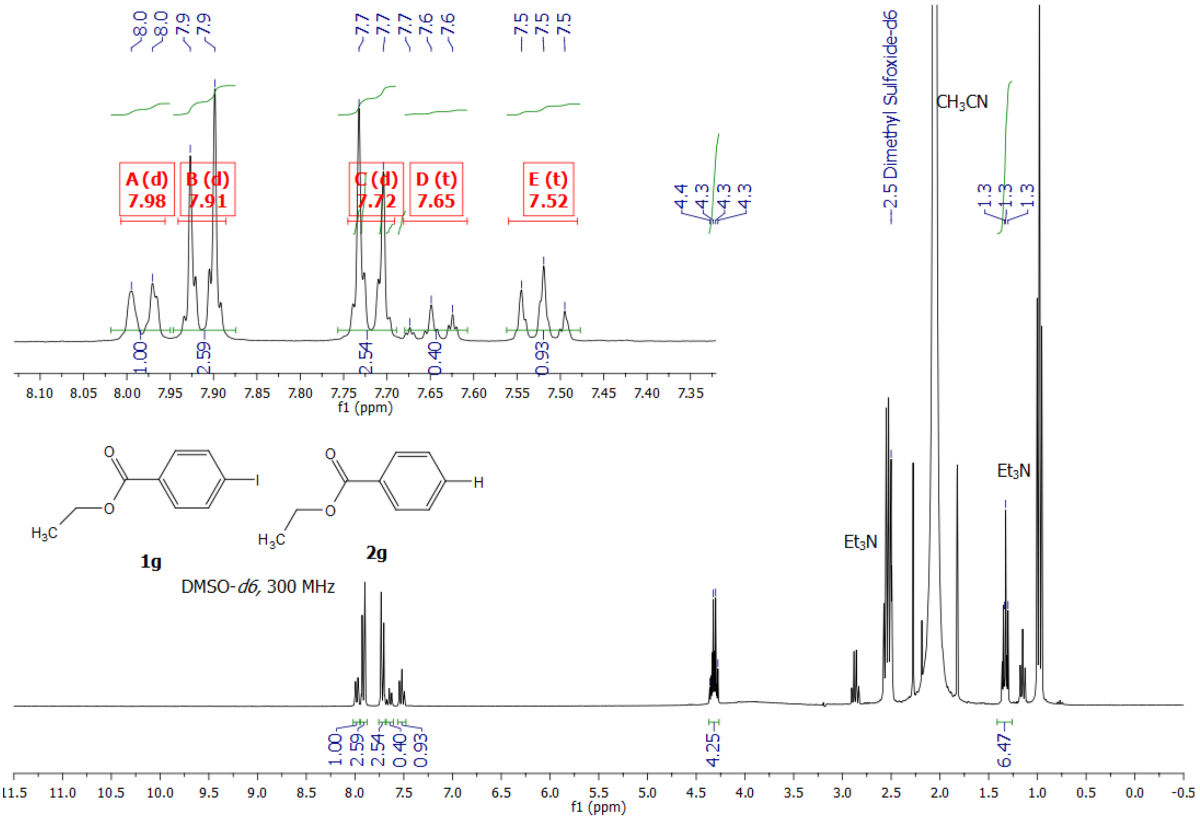

A (d) 7.98
B (d) 7.91
C (d) 7.72
D (t) 7.65
E (t) 7.52
2.5 Dimethyl Sulfoxide-d6
CH3CN
Et3N
Et3N
1g
2g
DMSO-d6, 300 MHz
f1 (ppm)

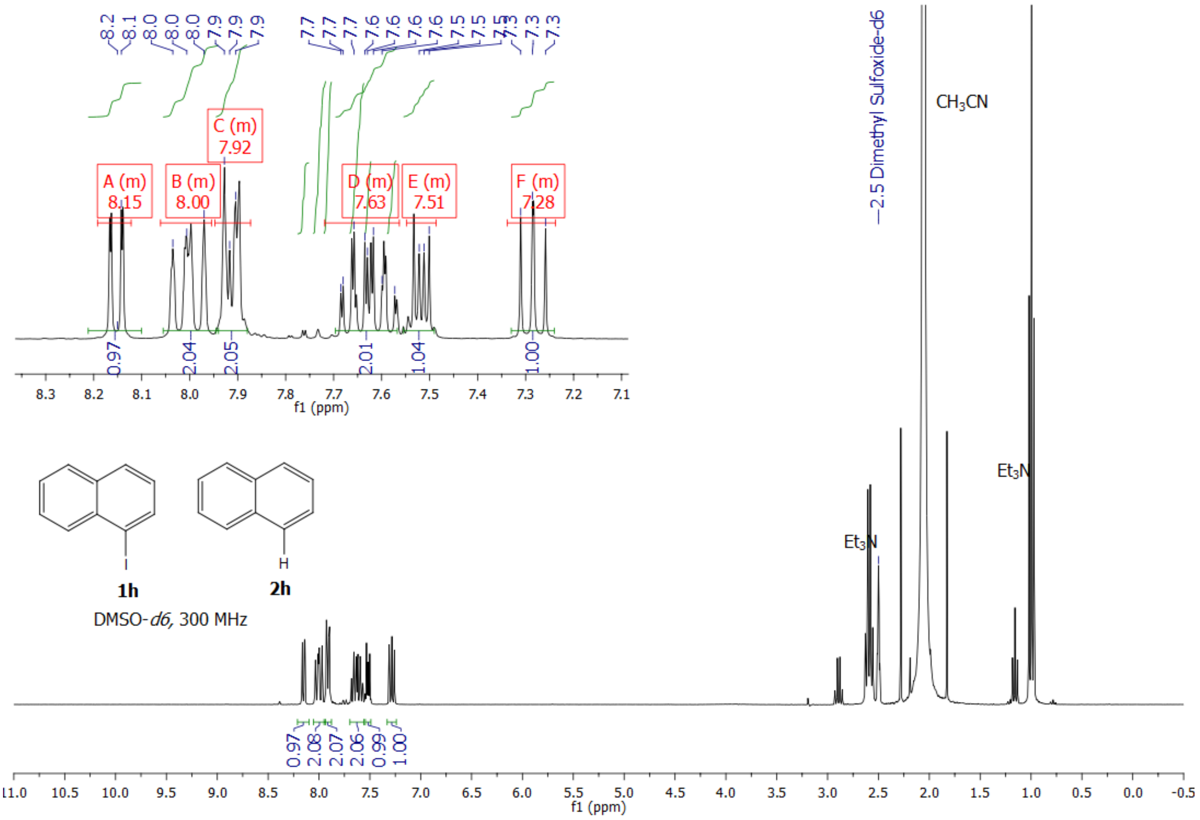
1h
2h
DMSO-d6, 300 MHz
2.5 Dimethyl Sulfoxide-d6
CH3CN
Et3N
Et3N
A (m) 8.15
B (m) 8.00
C (m) 7.92
D (m) 7.63
E (m) 7.51
F (m) 7.28
f1 (ppm)

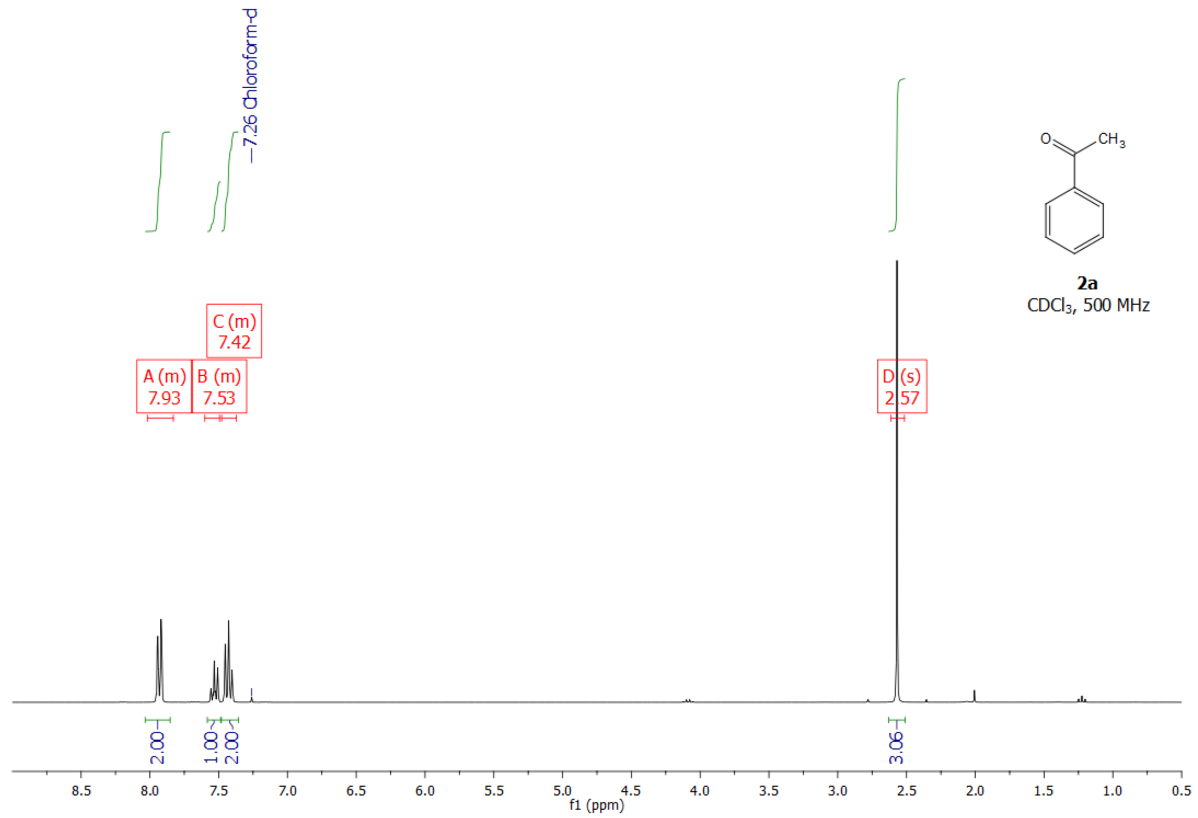
2a
CDCl3, 500 MHz
7.26 Chloroform-d
A (m) 7.93
B (m) 7.53
C (m) 7.42
D (s) 2.57
f1 (ppm)

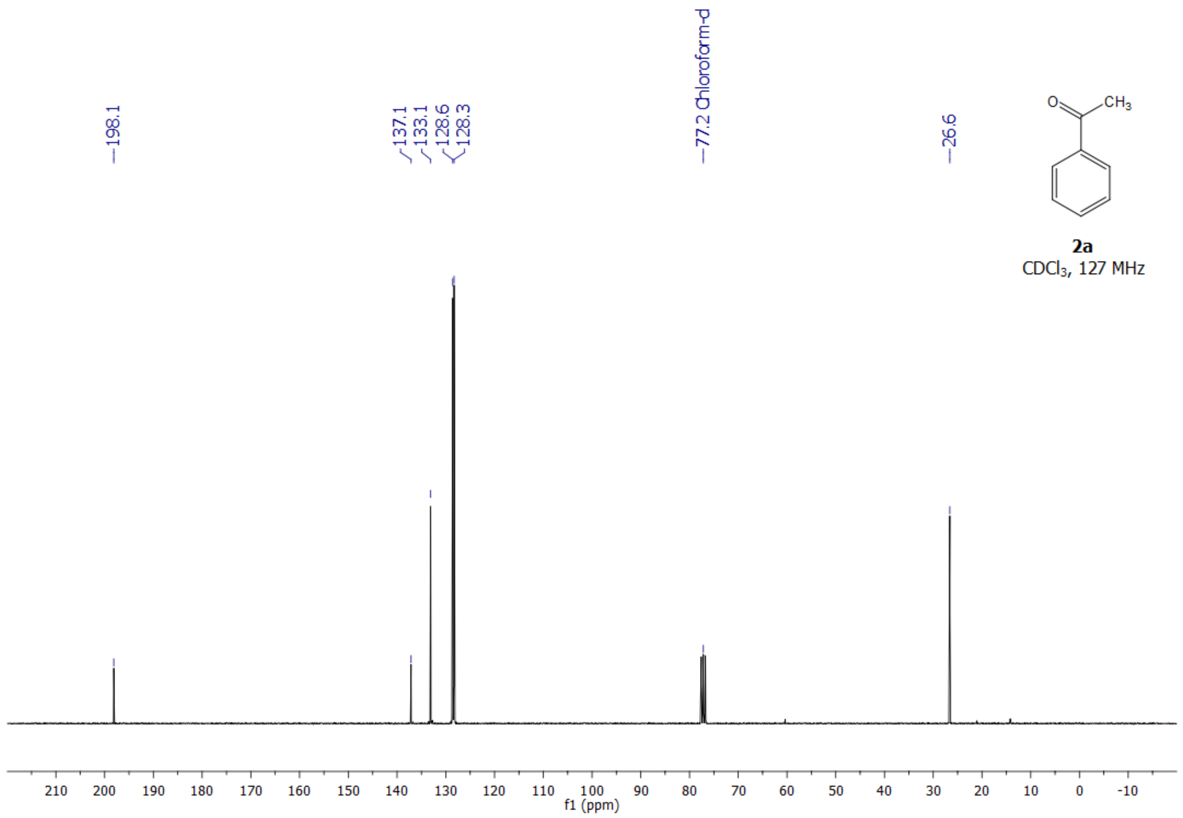
198.1
137.1
133.1
128.6
128.3
77.2 Chloroform-d
26.6
O
CH3
2a
CDCl3, 127 MHz
f1 (ppm)

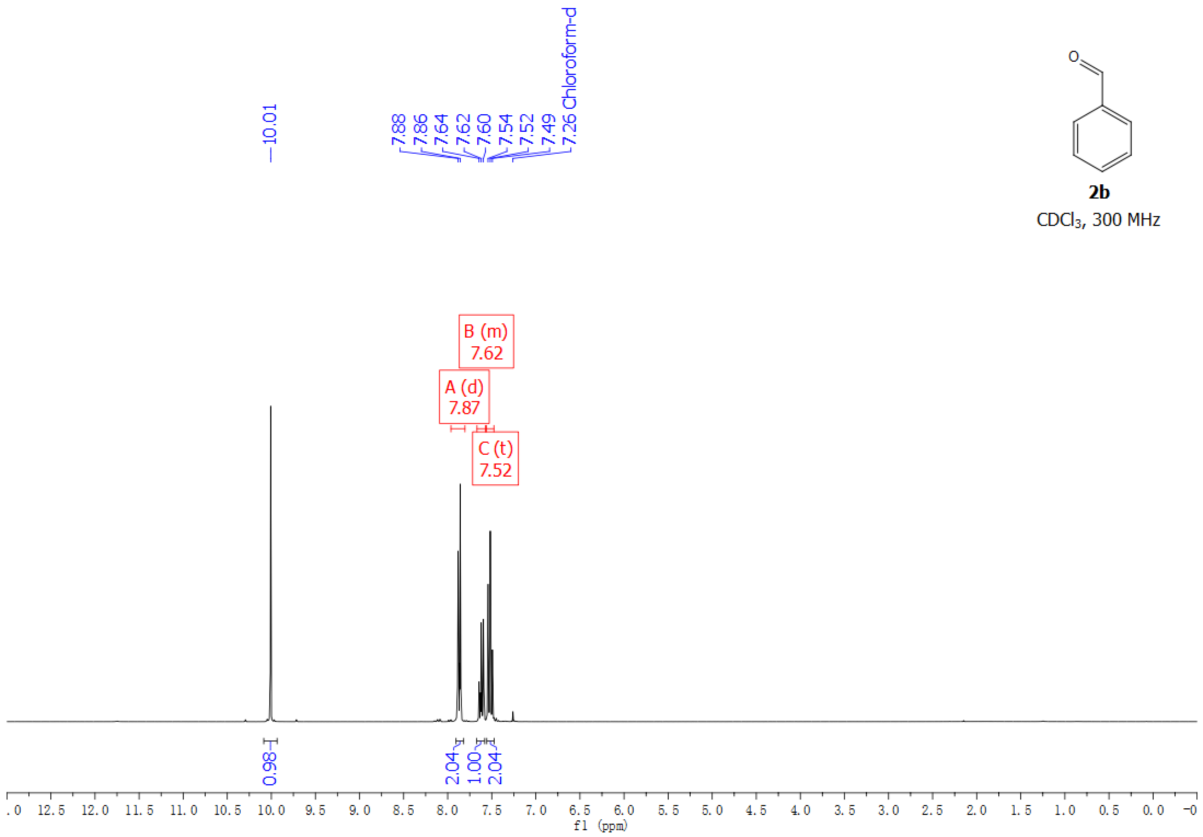
10.01
7.88
7.86
7.64
7.62
7.60
7.54
7.52
7.49
7.26 Chloroform-d
O
2b
CDCl3, 300 MHz
B (m)
7.62
A (d)
7.87
C (t)
7.52
0.98
2.04
1.00
2.04
f1 (ppm)

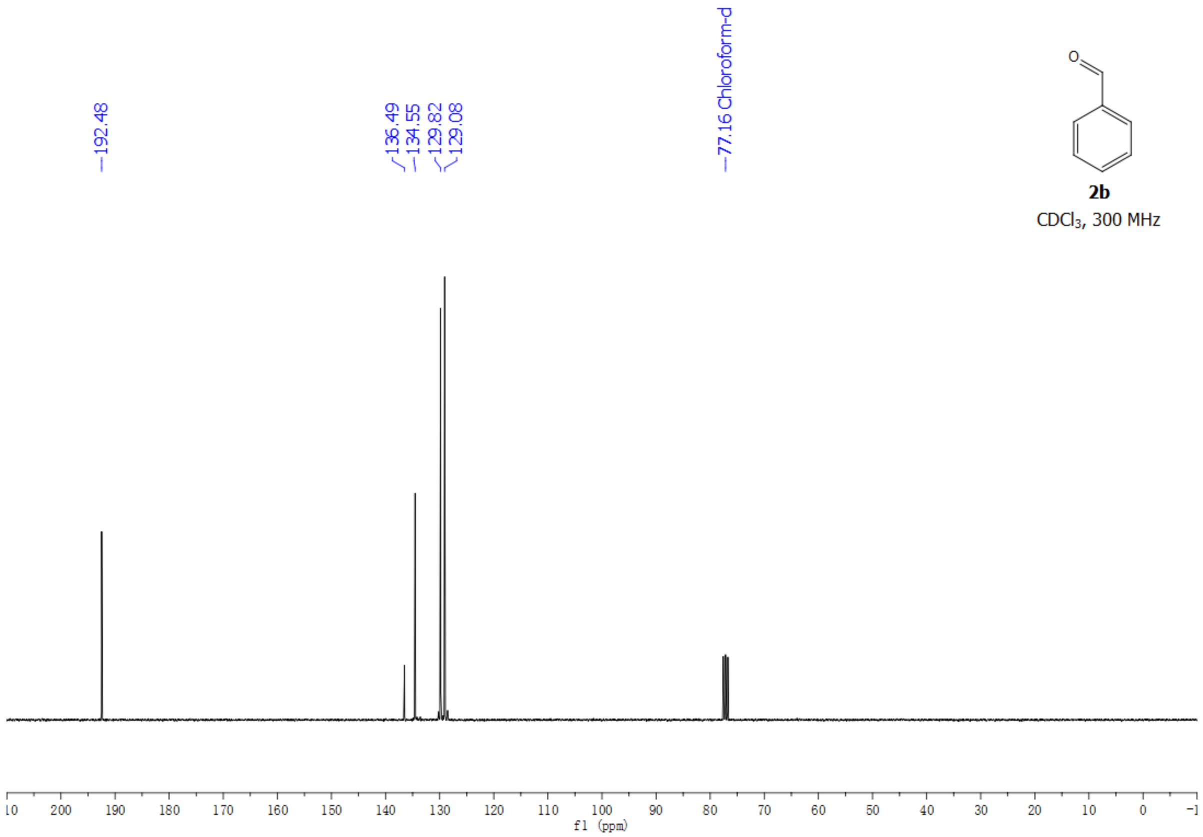
192.48
136.49
134.55
129.82
129.08
77.16 Chloroform-d
O
2b
CDCl3, 300 MHz
10 200 190 180 170 160 150 140 130 120 110 100 90 80 70 60 50 40 30 20 10 0 -1
f1 (ppm)

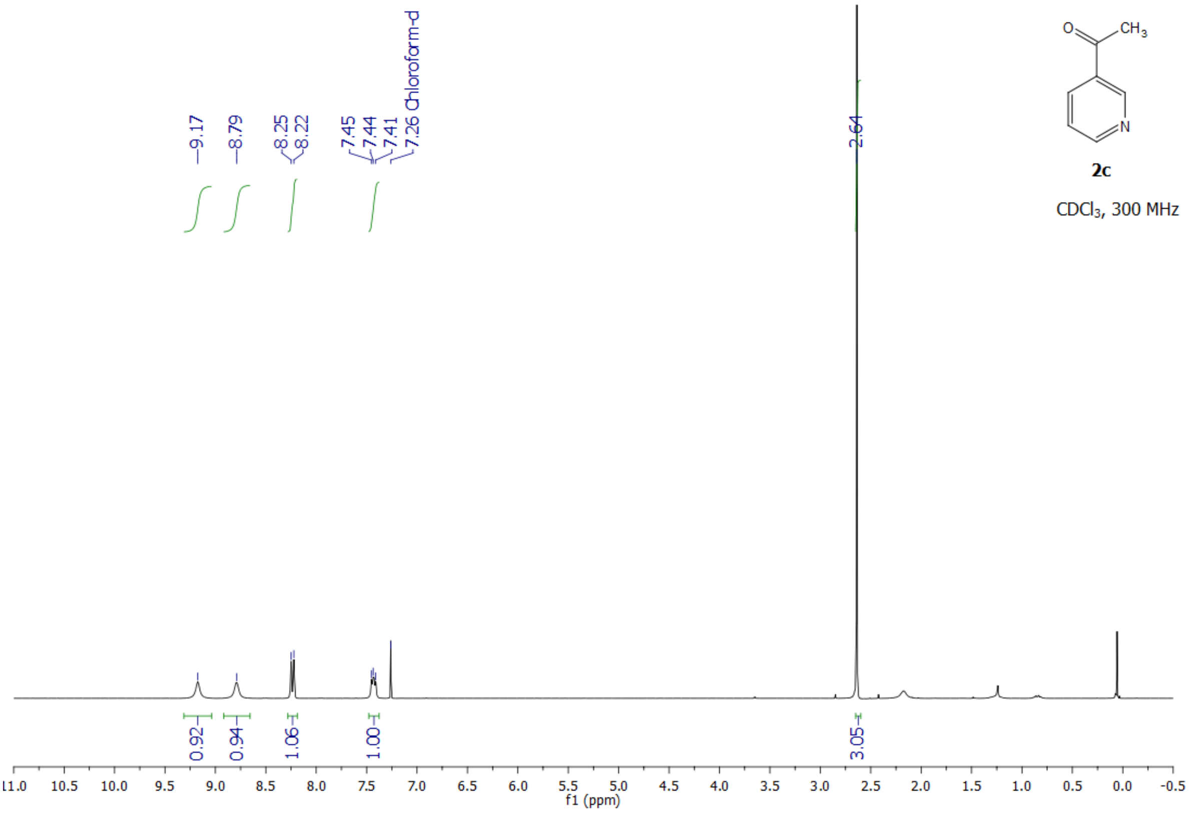
9.17
8.79
8.25
8.22
7.45
7.44
7.41
7.26 Chloroform-d
2.64
O
CH3
N
2c
CDCl3, 300 MHz
0.92
0.94
1.06
1.00
3.05
11.0 10.5 10.0 9.5 9.0 8.5 8.0 7.5 7.0 6.5 6.0 5.5 5.0 4.5 4.0 3.5 3.0 2.5 2.0 1.5 1.0 0.5 0.0 -0.5
f1 (ppm)

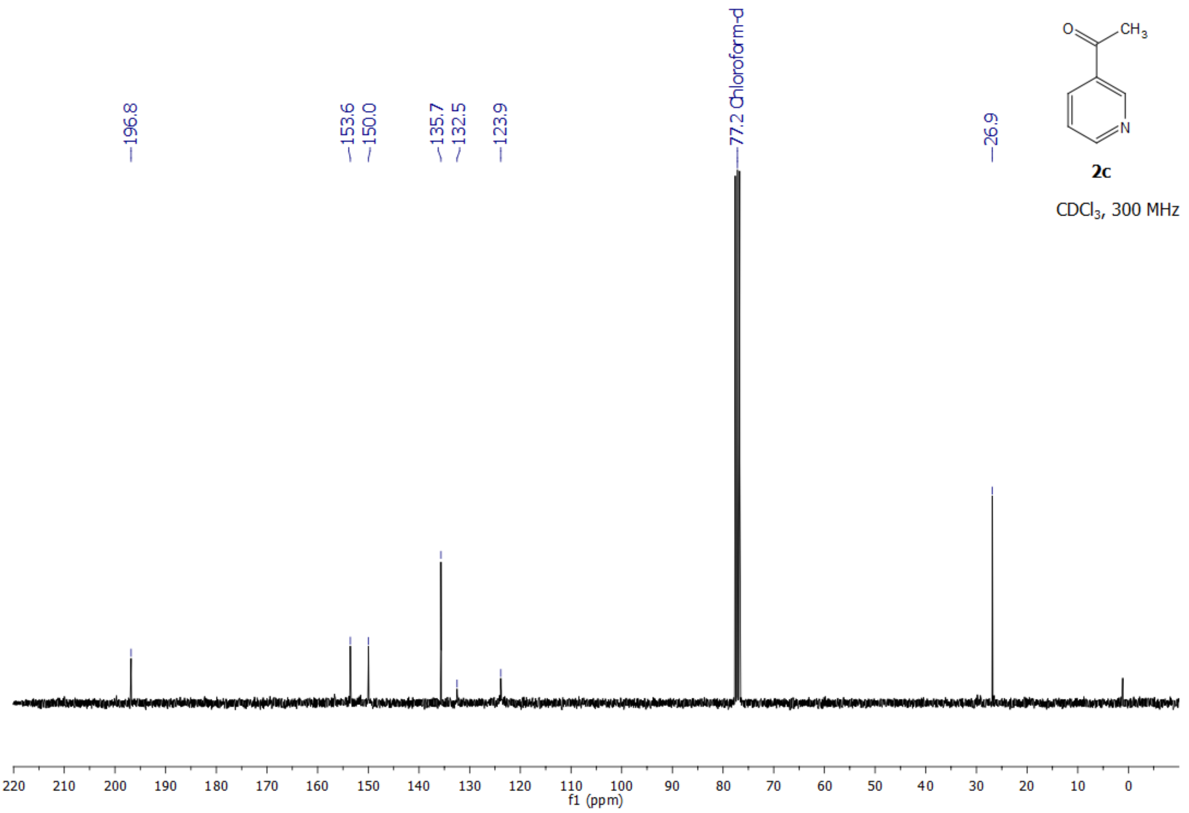
196.8
153.6
150.0
135.7
132.5
123.9
77.2 Chloroform-d
26.9
O
CH3
N
2c
CDCl3, 300 MHz
f1 (ppm)

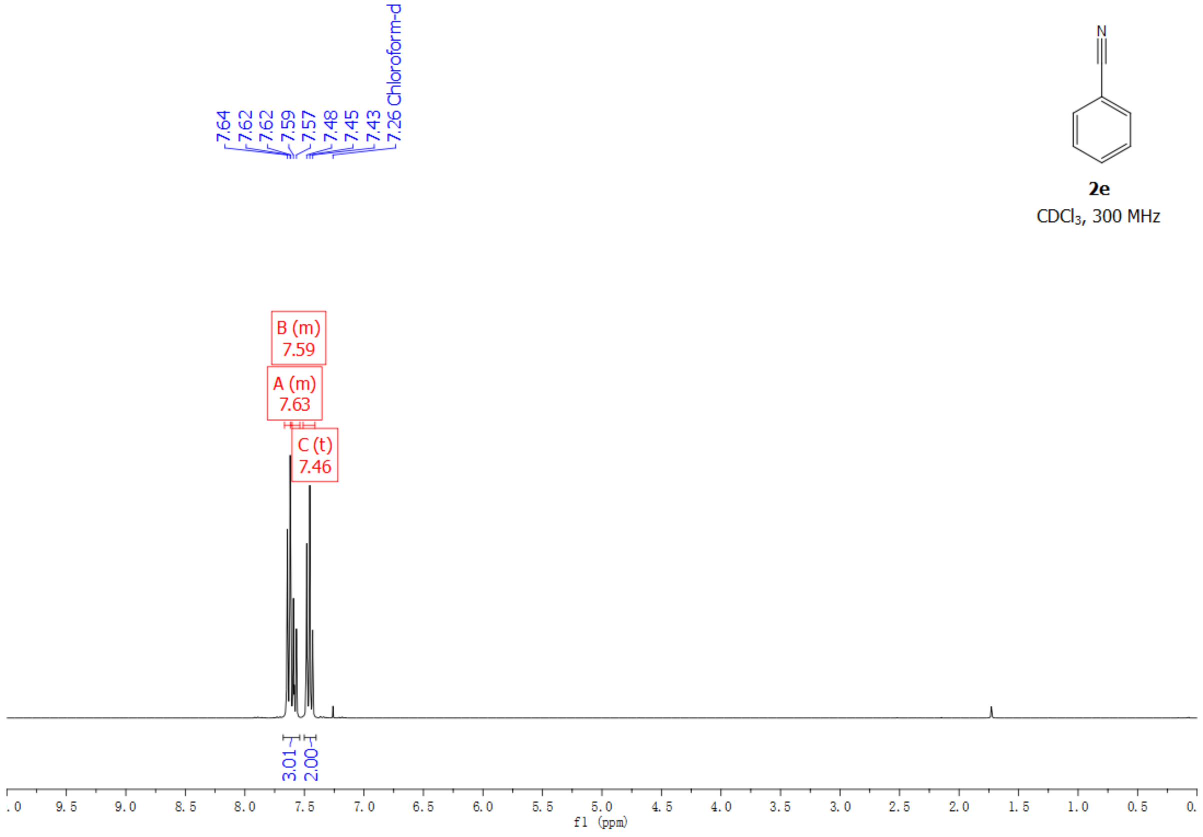
7.64
7.62
7.62
7.59
7.57
7.48
7.45
7.43
7.26 Chloroform-d
N
2e
CDCl3, 300 MHz
B (m)
7.59
A (m)
7.63
C (t)
7.46
3.01
2.00
f1 (ppm)

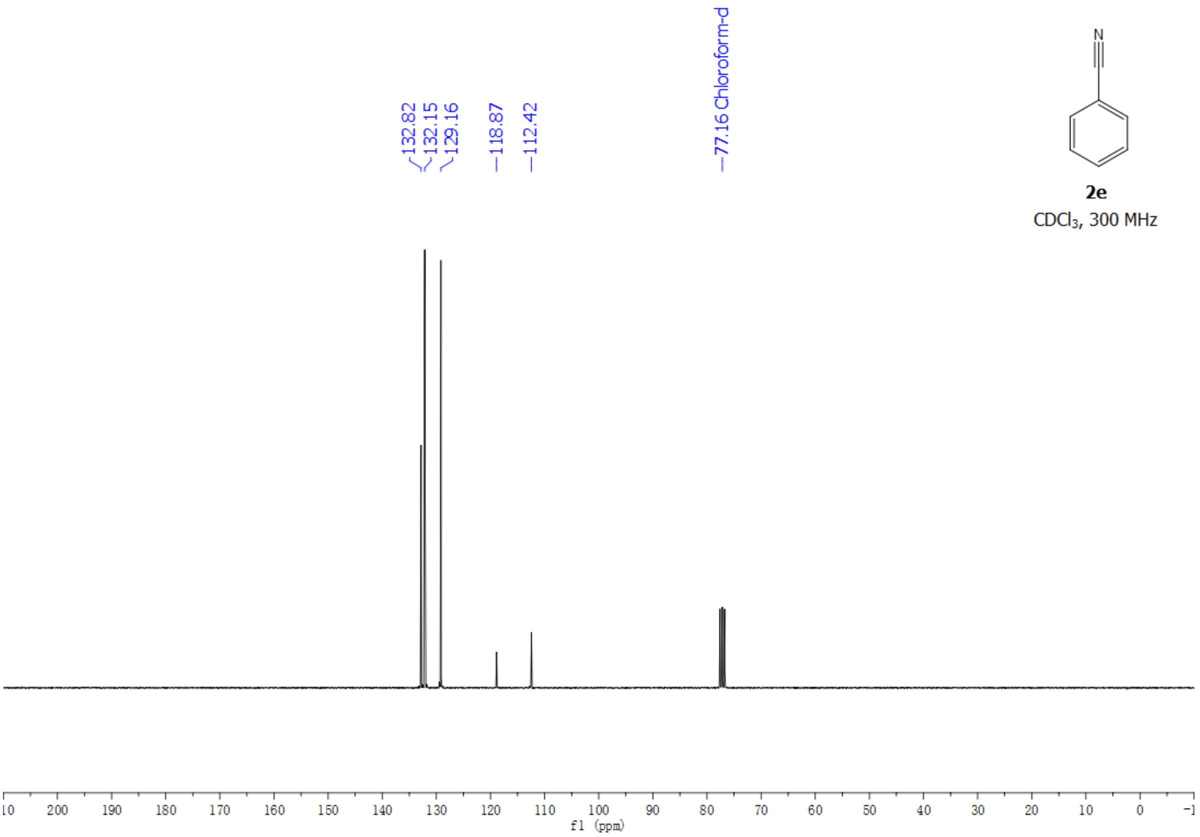

2e
$CDCl_3$, 300 MHz

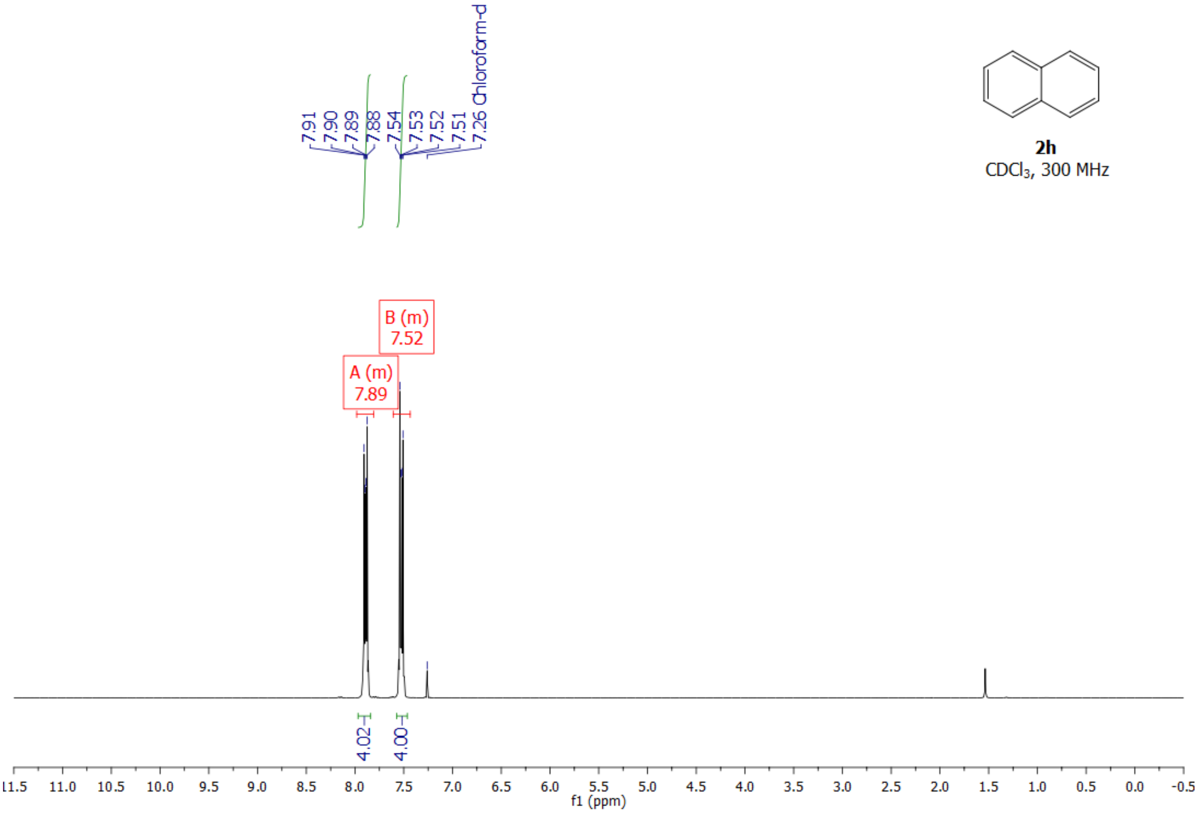

2h
$CDCl_3$, 300 MHz

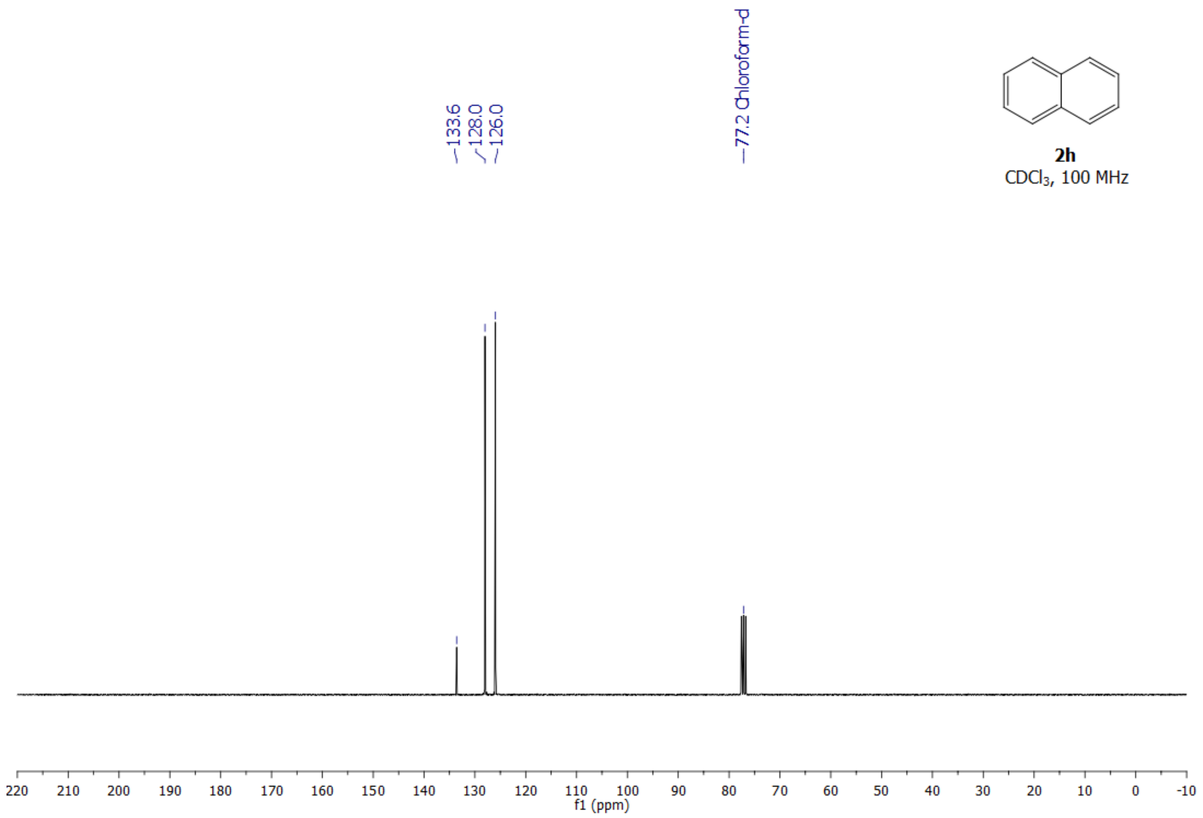

References

[1] M. Pinheiro, R. L. Martin, C. H. Rycroft, A. Jones, E. Iglesia, M. Haranczyk, *J. Mol. Graph. Model.* **2013**, *44*, 208–219.

[2] S. Ling, B. Slater, *J. Phys. Chem. C* **2015**, *119*, 16667–16677.

[3] S. Grimme, J. Antony, S. Ehrlich, H. Krieg, *J. Chem. Phys.* **2010**, *132*, 154104.

[4] R. Dovesi, A. Erba, R. Orlando, C. M. Zicovich-Wilson, B. Civalleri, L. Maschio, M. Rérat, S. Casassa, J. Baima, S. Salustro, B. Kirtman, *WIREs. Comput. Mol. Sci.* **2018**, *8*, 1–36.

[5] M. F. Peintinger, D. V. Oliveira, T. Bredow, *J. Comput. Chem.* **2013**, *34*, 451–459.

[6] J. D. Pack, H. J. Monkhorst, *Phys. Rev. B* **1977**, *16*, 1748–1749.

[7] J. Heyd, G. E. Scuseria, M. Ernzerhof, *J. Chem. Phys.* **2003**, *118*, 8207–8215.

[8] J. Heyd, G. E. Scuseria, M. Ernzerhof, *J. Chem. Phys.* **2006**, *124*, 219906.

[9] C. Serre, F. Millange, C. Thouvenot, M. Noguès, G. Marsolier, D. Louër, G. Férey, *J. Am. Chem. Soc.* **2002**, *124*, 13519–13526.

[10] A. C. Dippel, H. P. Liermann, J. T. Delitz, P. Walter, H. Schulte-Schrepping, O. H. Seeck, H. Franz, *J. Synchrotron Radiat.* **2015**, *22*, 675–687.

[11] J. Filik, A. W. Ashton, P. C. Y. Chang, P. A. Chater, S. J. Day, M. Drakopoulos, M. W. Gerring, M. L. Hart, O. V. Magdysyuk, S. Michalik, A. Smith, C. C. Tang, N. J. Terrill, M. T. Wharmby, H. Wilhelm, *J. Appl. Crystallogr.* **2017**, *50*, 959–966.

[12] P. Juhás, T. Davis, C. L. Farrow, S. J. L. Billinge, *J. Appl. Crystallogr.* **2013**, *46*, 560–566.

[13] T. C. Bowen, R. D. Noble, J. L. Falconer, *J. Memb. Sci.* **2004**, *245*, 1–33.

[14] S. H. Madani, A. Silvestre-Albero, M. J. Biggs, F. Rodríguez-Reinoso, P. Pendleton, *ChemPhysChem* **2015**, *16*, 3984–3991.

[15] M. Dan-Hardi, C. Serre, T. Frot, L. Rozes, G. Maurin, C. Sanchez, G. Férey, *J. Am. Chem. Soc.* **2009**, *131*, 10857–10859.

[16] Y. Horiuchi, T. Toyao, M. Saito, K. Mochizuki, M. Iwata, H. Higashimura, M. Anpo, M. Matsuoka, *J. Phys. Chem. C* **2012**, *116*, 20848–20853.
[17] N. M. Padial, J. Castells-Gil, N. Almora-Barrios, M. Romero-Angel, I. Da Silva, M. Barawi, A. García-Sánchez, V. A. De La Peña O'Shea, C. Martí-Gastaldo, *J. Am. Chem. Soc*. **2019**, *141*, 13124–13133.
[18] J. Gao, J. Miao, P. Z. Li, W. Y. Teng, L. Yang, Y. Zhao, B. Liu, Q. Zhang, *Chem. Commun*. **2014**, *50*, 3786–3788.
[19] C. Li, H. Xu, J. Gao, W. Du, L. Shangguan, X. Zhang, R. B. Lin, H. Wu, W. Zhou, X. Liu, J. Yao, B. Chen, *J. Mater. Chem. A* **2019**, *7*, 11928–11933.
[20] J. H. Cavka, S. Jakobsen, U. Olsbye, N. Guillou, C. Lamberti, S. Bordiga, K. P. Lillerud, *J. Am. Chem. Soc.* **2008**, *130*, 13850–13851.
[21] D. Sun, Y. Fu, W. Liu, L. Ye, D. Wang, L. Yang, X. Fu, Z. Li, *Chem. - A Eur. J.* **2013**, *19*, 14279–14285.
[22] L. Jiao, Y. Dong, X. Xin, R. Wang, H. Lv, *J. Mater. Chem. A* **2021**, *9*, 19725–19733.
[23] Y. Gao, S. Li, Y. Li, L. Yao, H. Zhang, *Appl. Catal. B Environ.* **2017**, *202*, 165–174.
[24] J. Gong, W. Zhang, T. Sen, Y. Yu, Y. Liu, J. Zhang, L. Wang, *ACS Appl. Nano Mater.* **2021**, *4*, 4513–4521.
[25] V. P. Viswanathan, S. V. Mathew, D. P. Dubal, N. N. Adarsh, S. Mathew, *ChemistrySelect* **2020**, *5*, 7534–7542.
[26] R. Long, Z. Yu, Q. Tan, X. Feng, X. Zhu, X. Li, P. Wang, *Appl. Surf. Sci.* **2021**, *570*, 151244.
[27] T. Tachikawa, J. R. Choi, M. Fujitsuka, T. Majima, *J. Phys. Chem. C* **2008**, *112*, 14090–14101.
[28] P. Li, J. Li, X. Feng, J. Li, Y. Hao, J. Zhang, H. Wang, A. Yin, J. Zhou, X. Ma, B. Wang, *Nat. Commun.* **2019**, *10*, 1–10.
[29] T. C. Narayan, T. Miyakai, S. Seki, M. Dincă, *J. Am. Chem. Soc.* **2012**, *134*, 12932–12935.
[30] R. Liang, R. Huang, X. Wang, S. Ying, G. Yan, L. Wu, *Appl. Surf. Sci.* **2019**, *464*, 396–403.
[31] T. Luo, Z. Wang, X. Han, Y. Chen, D. Iuga, D. Lee, B. An, S. Xu, X. Kang, F. Tuna, E. J. L. McInnes, L. Hughes, B. F. Spencer, M. Schröder, S. Yang, *CCS Chem.* **2022**, *4*, 2560–2569.
[32] A. Fateeva, P. A. Chater, C. P. Ireland, A. A. Tahir, Y. Z. Khimyak, P. V. Wiper, J. R. Darwent, M. J. Rosseinsky, *Angew. Chemie - Int. Ed.* **2012**, 124, 7558–7562.
[33] K. Guo, G. Jie, J. Liu, Y. Fu, R. Ma, X. Lu, F. Zhang, W. Zhu, M. Fan, *Sustain. Energy Fuels* **2022**, *113*, 5261.
[34] S. Nazari, E. Asgari, A. Sheikhmohammadi, S. A. Mokhtari, H. Alamgholiloo, *J. Environ. Chem. Eng.* **2023**, *11*, 110393.
[35] J. Castells-Gil, N. M. Padial, N. Almora-Barrios, J. Albero, A. R. Ruiz-Salvador, J. González-Platas, H. García, C. Martí-Gastaldo, *Angew. Chemie - Int. Ed.* **2018**, *57*, 8453–8457.
[36] Y. Guo, J. Zhang, L. Z. Dong, Y. Xu, W. Han, M. Fang, H. K. Liu, Y. Wu, Y. Q. Lan, *Chem. - A Eur. J.* **2017**, *23*, 15518–15528.
[37] J. Ling, A. Zhou, W. Wang, X. Jia, M. Ma, Y. Li, *ACS Omega* **2022**, *7*, 19920–19929.
[38] K. Song, S. Liang, X. Zhong, M. Wang, X. Mo, X. Lei, Z. Lin, *Appl. Catal. B Environ.* **2022**, *309*, 121232.
[39] F. Guo, S. Yang, Y. Liu, P. Wang, J. Huang, W. Y. Sun, *ACS Catal.* **2019**, *9*, 8464–8470.
[40] T. Luo, L. Li, Y. Chen, J. An, C. Liu, Z. Yan, J. H. Carter, X. Han, A. M. Sheveleva, F. Tuna, E. J. L. McInnes, C. C. Tang, M. Schröder, S. Yang, *Nat. Commun.* **2021**, *12*, 1–10.
[41] S. Oudi, A. R. Oveisi, S. Daliran, M. Khajeh, R. Luque, U. Sen, H. García, *Appl. Catal. A Gen.* **2021**, *611*, 117965.
[42] B. Chen, L. Liu, Y. Song, H. Liu, Z. Gong, Y. She, J. Liu, R. Niu, J. Gong, Mater. *Today Sustain.* **2023**, *24*, 100561.
[43] S. Hosseini, J. N. Janusz, M. Tanwar, A. D. Pendergast, M. Neurock, H. S. White, *J. Am. Chem. Soc.* **2022**, *144*, 21103–21115.
[44] Y. M. Yang, W. Yan, H. W. Hu, Y. Luo, Z. Y. Tang, Z. Luo, *J. Org. Chem.* **2021**, *86*, 12344–12353.
[45] A. Wang, H. Jiang, *J. Org. Chem.* **2010**, *75*, 2321–2326.
[46] J. H. An, K. D. Kim, J. H. Lee, *J. Org. Chem.* **2021**, *86*, 2876–2894.
[47] A. C. Bissember, M. G. Banwell, *J. Org. Chem.* **2009**, *74*, 4893–4895.

[48] P. Boehm, T. Martini, Y. H. Lee, B. Cacherat, B. Morandi, *Angew. Chemie - Int. Ed.* **2021**, *60*, 17211–17217.
[49] R. Ding, Y. Liu, M. Han, W. Jiao, J. Li, H. Tian, B. Sun, *J. Org. Chem.* **2018**, *83*, 12939–12944.
[50] H. J. Li, R. Guillot, V. Gandon, *J. Org. Chem.* **2010**, *75*, 8435–8449.
[51] W. Liu, X. Yang, Y. Gao, C. J. Li, *J. Am. Chem. Soc.* **2017**, *139*, 8621–8627.
[52] V. Ganesan, S. Moon, S. Yoon, *J. Org. Chem.* **2023**, *88*, 5127–5134.
[53] A. V. Iosub, S. S. Stahl, *J. Am. Chem. Soc.* **2015**, *137*, 3454–3457.
